# New 1,3-diphenyl-1*H*-pyrazol-5-ols as anti-methicillin resistant *Staphylococcus aureus* agents: Synthesis, antimicrobial evaluation and *in silico* studies

**DOI:** 10.1016/j.heliyon.2024.e33160

**Published:** 2024-06-25

**Authors:** Mohamed A.M. Abdel Reheim, Ibrahim S. Abdel Hafiz, Hala M. Reffat, Hend S. Abdel Rady, Ihsan A. Shehadi, Huda R.M. Rashdan, Abdelfattah Hassan, Aboubakr H. Abdelmonsef

**Affiliations:** a Department of Chemistry, Faculty of Science, Arish University, Arish, 45511, Egypt; b Chemistry Department, College of Sciences, University of Sharjah, Sharjah, 27272, United Arab Emirates; c Chemistry of Natural and Microbial Products Department, Pharmaceutical and Drug Industries Research Institute, National Research Centre, 33 El Buhouth St, Dokki, Giza, 12622, Egypt; d Department of Medicinal Chemistry, Faculty of Pharmacy, South Valley University, Qena, 83523, Egypt; e Chemistry Department, Faculty of Science, South Valley University, Qena, 83523, Egypt

**Keywords:** 4-Acetyl-1,3-diphenyl-1*H*-pyrazole-5(4*H*)-ole, Pyrazole, Antibiotic resistance, Antimicrobial activities, DNA gyrase, Molecular docking

## Abstract

In the present work, two hybrid series of pyrazole-clubbed pyrimidine and pyrazole-clubbed thiazole compounds **3–21** from 4-acetyl-1,3-diphenyl-1*H*-pyrazole-5(4*H*)-ole **1** were synthesized as novel antimicrobial agents. Their chemical structures were thoroughly elucidated in terms of spectral analyses such as IR, ^1^H NMR, ^13^C NMR and mass spectra. The compounds were *in vitro* evaluated for their antimicrobial efficiency against various standard pathogen strains, gram -ive bacteria (*Pseudomonas aeruginosa*, *Klebsiella pneumonia*), gram + ive bacteria (MRSA, *Bacillus subtilis*), and Unicellular fungi (*Candida albicans*) microorganisms. The ZOI results exhibited that most of the tested molecules exhibited inhibition potency from moderate to high. Where compounds **7**, **8**, **12**, **13** and **19** represented the highest inhibition potency against most of the tested pathogenic microbes comparing with the standard drugs. In addition, the MIC results showed that the most potent molecules **7**, **8**, **12**, **13** and **19** showed inhibition effect against most of the tested microbes at low concentration. Moreover, the docking approach of the newly synthesized compounds against DNA gyrase enzyme was performed to go deeper into their molecular mechanism of antimicrobial efficacy. Further, computational investigations to calculate the pharmacokinetics parameters of the compounds were performed. Among them **7**, **8**, **12**, **13** and **19** are the most potent compounds revealed the highest inhibition efficacy against most of the tested pathogenic microbes comparing with the standard drugs.

## 1 Introduction

Fleming was the first who admonish about the risk of the global antibiotic resistance [[1], [2], [3]]. The antibiotic resistance was first identified in *Shigella*, *Salmonella* and *E. Coli* [[4], [5], [6]]. Owing to the antibiotic misuse, where these antibiotics are available and used without prescriptions, the bacteria acquired antibiotic resistance against most of the commercially available antibiotics [4,5,7,8]. Consequently, resistance of the antibiotics is considered as a global public health concern by the world health organization (WHO) [9]. Methicillin-resistant *Staphylococcus aureus* (MRSA) infections pose a serious clinical and financial burden to people all over the world. MRSA is turning into a fatal sickness for humans because it is easily transmitted, causes severe skin infections, and resists most recognized antibiotics, including vancomycin. Consequently, identify potential antimicrobial drug candidates to overcome MRSA isolates is urgently required [10].

**Fig. 1:**
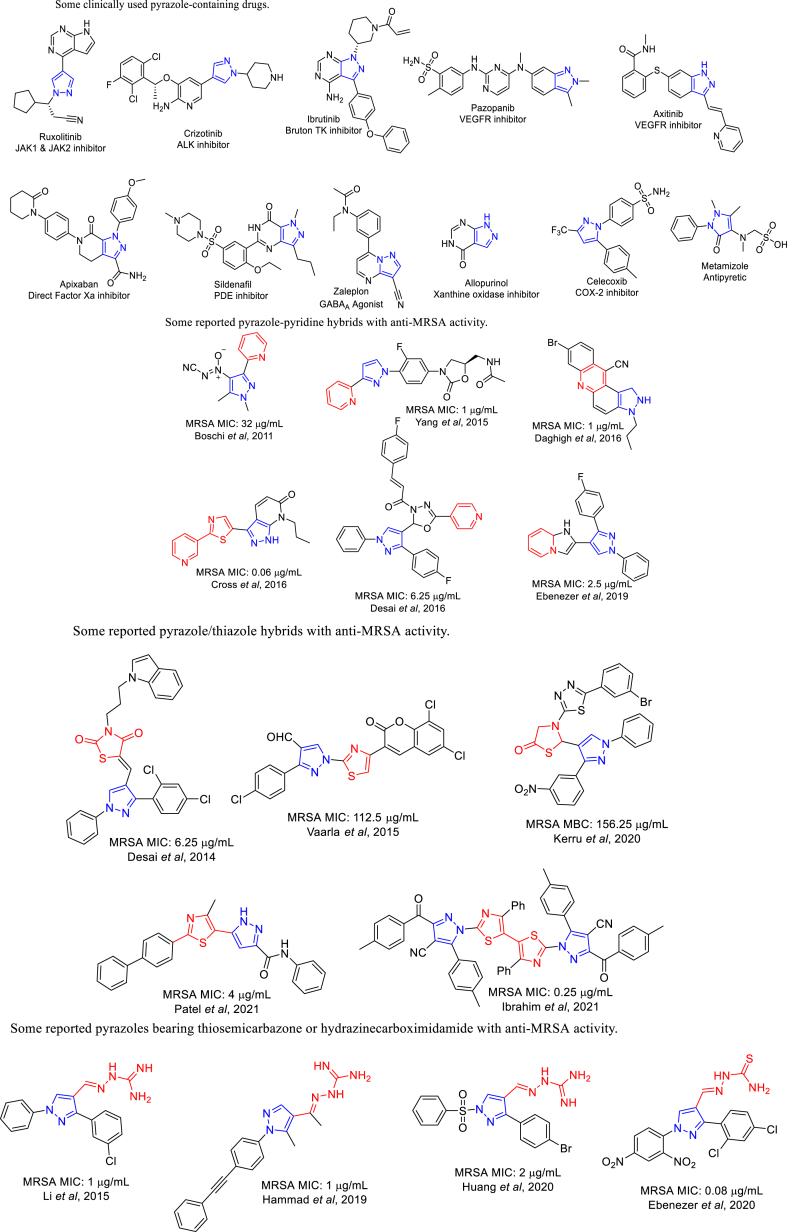
Some clinically used pyrazole-containing drugs, reported pyrazole-pyridine hybrids with anti-MRSA activity, reported pyrazole/thiazole hybrids with anti-MRSA activity and reported pyrazoles bearing thiosemicarbazone or hydrazinecarboximidamide with anti-MRSA activity.

Heterocyclic compounds incorporating pyrazole moiety constitute an essential class of biologically active compounds that are gaining an attention in medicinal bioorganic chemistry [[11], [12], [13], [14]]. Many clinically used drugs contain pyrazole moiety, as shown in Fig. 1. The most important class of pyrazole-containing drugs is tyrosine kinase inhibitors like ruxolitinib (Jakafi®) which is used in myelofibrosis, and polycythemia vera, crizotinib (Xalkori®) which is indicated in treatment of non-small cell lung cancer, ibrutinib (Imbruvica®) which is indicated in chronic lymphocytic leukemia and finally axitinib (Inlyta®) and pazopanib (Votrient®) which are indicated in advanced renal cell carcinoma [15]. Vicinal diaryl azoles were utilized as scaffolds for selective cyclooxygenase-2 (COX-2) inhibition [16]. One of these azoles is a pyrazole which was found in celecoxib (Celebrex®) which is still clinically used till now. Metamizole (Novalgin®) is a sulfonic acid-containing pyrazole derivative which is still used as antipyretic till now despite its several adverse effects. Sildenafil (Viagra®) is a pyrazolopyrimidine phosphodiesterase 5 (PDE5) inhibitor which is used in the treatment of erectile dysfunction. Other pyrazole-containing drugs like anticoagulant, apixaban (Eliquis®), allopurinol (Zyloprim®) and a non-benzodiazepine zaleplon (Sonata®) are used as sedatives, hypnotics and anxiolytics [10].

Pyridine was incorporated in plenty of reported antimicrobial derivatives [17]. Several pyrazole-pyridine hybrids were reported to have markable antimicrobial activity especially against MRSA [18], as declared in Fig. 1. Thiazolidine was fused with a β-lactam ring to form a penam ring which is the main pharmacophoric element of penicillin antibiotics as well as β-lactamase inhibitors like sulbactam and tazobactam [19]. Moreover, aminothiazole moiety was incorporated in 3rd and 4th generation cephalosporines like cefotaxime, ceftriaxone and cefepime to improve their activity against gram -ive bacteria. The 5th generation cephalosporine like ceftaroline with thiazole ring is characterized by its antibacterial activity against MRSA [19]. Additionally, molecular hybridization of pyrazole ring with thiazole afforded several hybrids with remarkable antibacterial and antifungal activity [20], as represented in Fig. 1. Thiosemicarbazone and its isostere hydrazinecarboximidamide were reported to be involved in several antimicrobial derivatives especially with pyrazole ring that have a remarkable activity against MRSA [21], as represented in Fig. 1.

DNA gyrase is considered as a crucial target for design and development of effective antibacterial inhibitors due to its ability to induce negative supercoiling of DNA or relieve positive supercoiling [20]. Gyrase is present in prokaryotes and specific eukaryotes, albeit with variations in structure and sequence, resulting in different affinities for diverse derivatives [[22], [23], [24]]. Furthermore, it is an extensively researched objective that is essential for bacterial DNA replication [25]. Consequently, the justification for utilizing DNA gyrase is mainly substantiated by reported pyrazole-containing compounds, which have strong affinity for this protein.

The molecular docking approach is a technique used to discover structures for the active sites of proteins, in addition to identify the potential mechanism of action [[26], [27], [28]].

Viewing the importance of heterocyclic compounds incorporating pyrazole ring in medicinal chemistry field and to highlight the scope of compounds containing pyrazole, pyridine and/or thiazole moiety, herein, synthesis of two hybrid series of pyrazole-clubbed pyrimidine and pyrazole-clubbed thiazole derivatives **3–21** from compound **1** is described. The antimicrobial potency of the newly prepared compounds was estimated against the various standard pathogen strains, gram -ive bacteria (*P. aeruginosa*, *K. pneumonia*), gram + ive bacteria (MRSA, *B. subtilis*), and fungi (*C. albicans*) microorganisms. Further, *in silico* docking approaches [25,[29], [30], [31]] of the molecules were performed to investigate their binding interactions with DNA gyrase enzyme.

## 2 Results and discussion

### 2.1 Chemistry

**Scheme 1:**
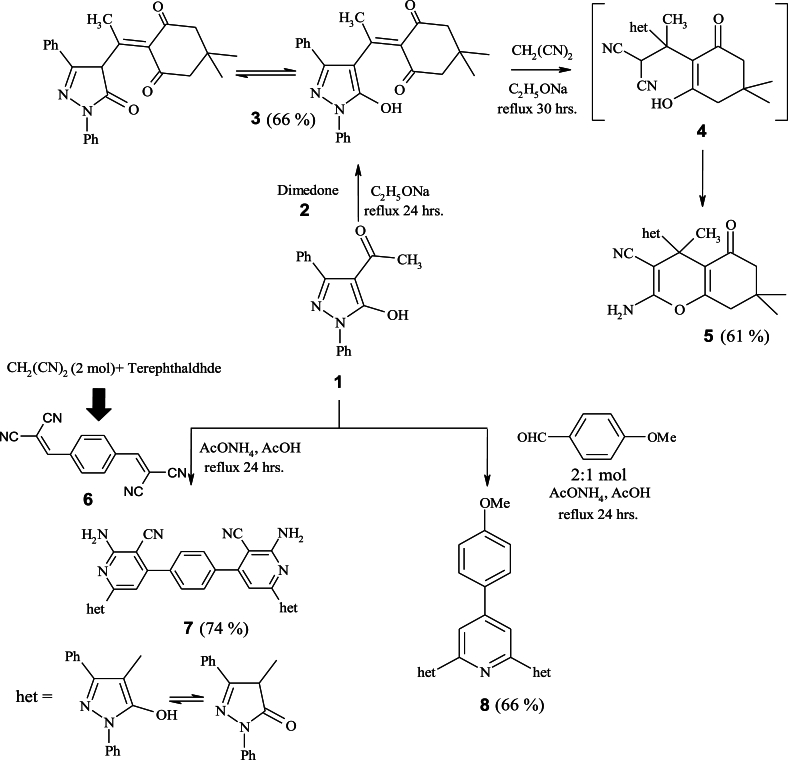
Synthesis of compounds **3**, **5**, **7** and **8**.

The starting compound **1** was prepared using a well-known process from the literature [32]. It was used as a precursor to prepare a series of novel heterocyclic compounds *via* one pot multi component reaction (MCR). Thus, condensation reaction of **1** with dimedone's active methylene **2** yielded the arylidene derivative **3**, which creates a cyclic intermediate **4** by adding malononitrile to the activated double bond, followed by cyclization to furnish 5,6,7, 8-tetrahydro-4*H*-chromene-3-carbonitrile **5** [33] (Scheme 1). Through elemental and spectral analyses, the structure of product **5** was verified. IR spectrum exhibited absorption bands at *ν* 3449, 2198, and 1710 cm^−1^ for –NH_2_, –CN and –CO, respectively. Moreover, ^1^H NMR analysis exhibited the following signals at *δ* 0.94 (s, 6H, 2CH_3_), 2.19 (s, 3H, CH_3_), 3.51 (s, 2H, CH_2_), 3.62 (s, 2H, CH_2_), 6.02 (s, 2H, NH_2_), 7.29–7.83 (m, 10H, Ar–H), 14.49 (s, 1H, OH). However, a parent peak at *m/z* (%) 466 (M^+^) is in agreement with the proposed structure.

**Scheme 2:**
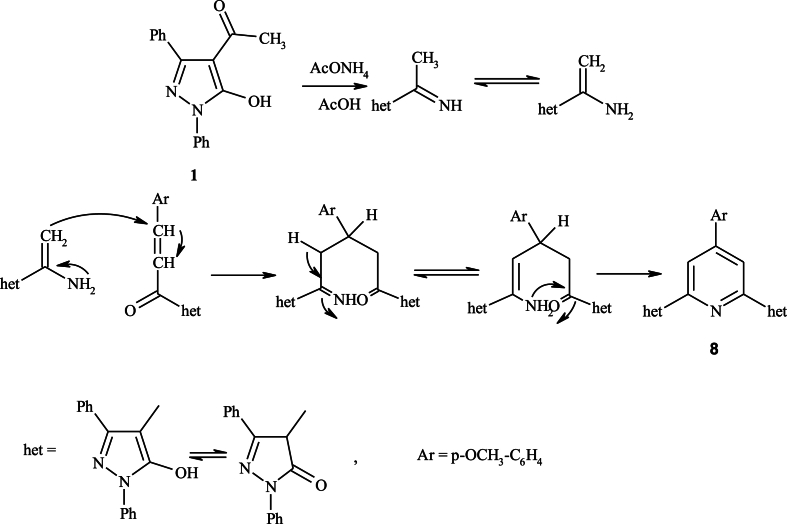
The suggested mechanism for preparation of product **8**.

Considerable attention has been devoted to pyrazole-pyridine hybrids as antimicrobial agents [34,35]. By using MCR, our research was expanded to create a novel bipyridine rings clubbed pyrazole moiety **7** *via* refluxing of **1** with terephthaldehyde, malononitrile, and ammonium acetate in glacial acetic acid. These hypotheses were confirmed by allowing derivative **1** to interact with arylidene malononitrile, which was made by condensing (terephthaldehyde with malononitrile). Spectral and elemental analyses were used to clarify the product's structure [36]. IR spectrum revealed absorption bands at *ν* 3461–3328, 3058, 2195 cm^−1^ assigned to (-OH/–NH_2_), CH-_aromatic_, and –CN groups, respectively. However, ^1^H NMR analysis showed one signal at ™ 6.10 ppm for –NH_2_, along with expected signals for the aryl and hydroxyl protons. Additionally, the molecular ion peak in the mass spectrum is compatible with the suggested structure. On the other hand, reaction of 2 mol of **1** with substituted aromatic aldehyde and ammonium acetate at 160 °C yielded 4,4'-(4-(4-methoxyphenyl)pyridine-2,6-diyl)bis(1,3-diphenyl-1H-pyrazol-5-ol **8** [37]. IR spectrum revealed the existence of OH-specific bands at 3449, CH-_aromatic_ at 3060, CH-_aliphatic_ at *ν* 2931 cm^−1^. Its ^1^H NMR analysis displayed a singlet peak at *δ* 3.71 ppm assigned to –OCH_3_ protons, a singlet peak at *δ* 5.23 ppm attributed to two protons of the pyridine ring, multiplet peaks at ™ 6.87–7.84 ppm corresponding to Ar–H. Further, the –OH group appeared at *δ* 14.32 ppm. The parent peak at the *m/z* 653 (M^+^) indicated by the appropriate molecular formula might be seen in the mass spectrum. The proposed mechanism for formation of compound **8** is presented in Scheme 2.

**Scheme 3:**
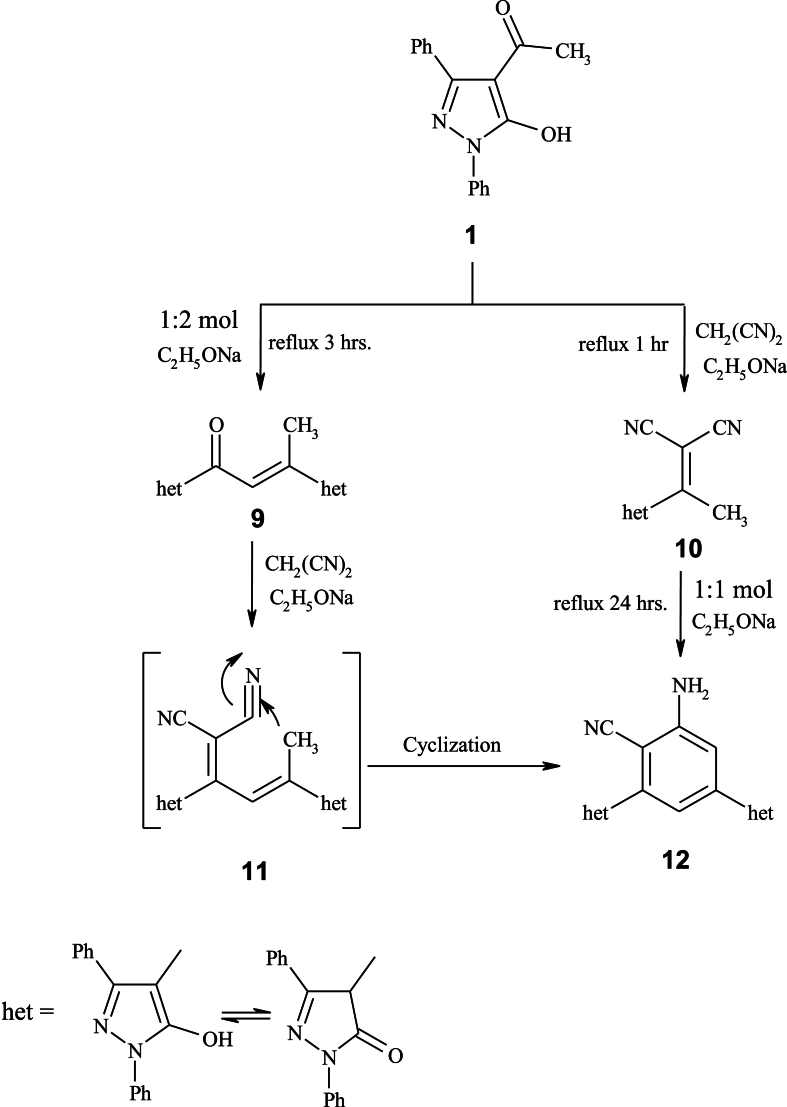
Synthesis of compound **12**.

Based on spectral data, product **9** that resulted from the condensation of 2 mol of 4-acetylpyrazole-5-ole **1**, was confirmed. Moreover, reaction of **9** with malononitrile furnished 2-amino-4,6-bis(5-hydroxy-1,3-diphenyl-1*H*-pyrazol-4-yl)benzonitrile **12**. The formation of **12** could be explained by condensation of **9** with malononitrile to yield an intermediate **11**, which undergoes intermolecular cyclization through the nucleophilic addition of methyl group to one of the carbonitrile function followed by aromatization (Scheme 3). On the other hand, using analytical and spectral data, condensation of **1** with malononitrile in sodium ethoxide (EtONa) produced 2-(1-(5-hydroxy-1,3-diphenyl-1*H*-pyrazol-4-yl)ethylidene)malononitrile **10**. Then, by cyclizing compound **10** with **1**, the benzonitrile derivative **12** was formed, confirming the chemical composition of **12** [38].

Synthesis of compounds incorporating pyrazole moiety attached to thiazole and/or thiazolidine cores **15**, **16**, **19** and **21** is of particular interest because of their antimicrobial activities [20]. In this study, we also described a very effective synthetic method for certain thiazole and/or thiazolidine scaffolds attached to pyrazole moiety. The crucial step, 2-(1-(5-hydroxy-1,3-diphenyl-1*H*-pyrazole-4-yl)ethylidene) hydrazinecarbothioamide **13**, was produced by reacting **1** with thiosemicarbazide in an acidic medium. The latter compound **13** was reacted with ethyl 2-chloro-2-(2-(4-methoxyphenyl)hydrazono)acetate **14** to yield thiazole derivative **15**. The IR analysis presented absorption bands at *ν* 3300, 3128, 3028, 2881 and 1685 cm^−1^ characteristic of (-OH/–NH–), CH-_aromatic_, CH-_aliphatic_ and –CO groups, respectively. However, ^1^H NMR spectrum declared characteristic singlet signals in the region ™ 6.02, 6.50 ppm referred to NH protons. The parent peak at 527 (M^+^+2) confirmed the hypothesized structure.

The thiazolidinone **16** was produced *via* reaction of **13** with ethyl-2-chloroacetate and/or chloroacetic. ^1^H NMR spectrum represented the existence of a singlet peak at ™ 1.25 ppm referred to –CH_3_ group, multiple peaks at ™ 7.28–7.86 ppm for Ar–H, along with a singlet peak at ™ 5.02 ppm referred to –CH_2_ group, a peak of NH proton at ™ 6.06 ppm, and hump one at ™ 11.80 ppm corresponding to –OH group. However, the MS of **16** showed the parent peak at 393 (M^+^+2), assignable to C_20_H_17_N_5_O_2_S.

**Scheme 4:**
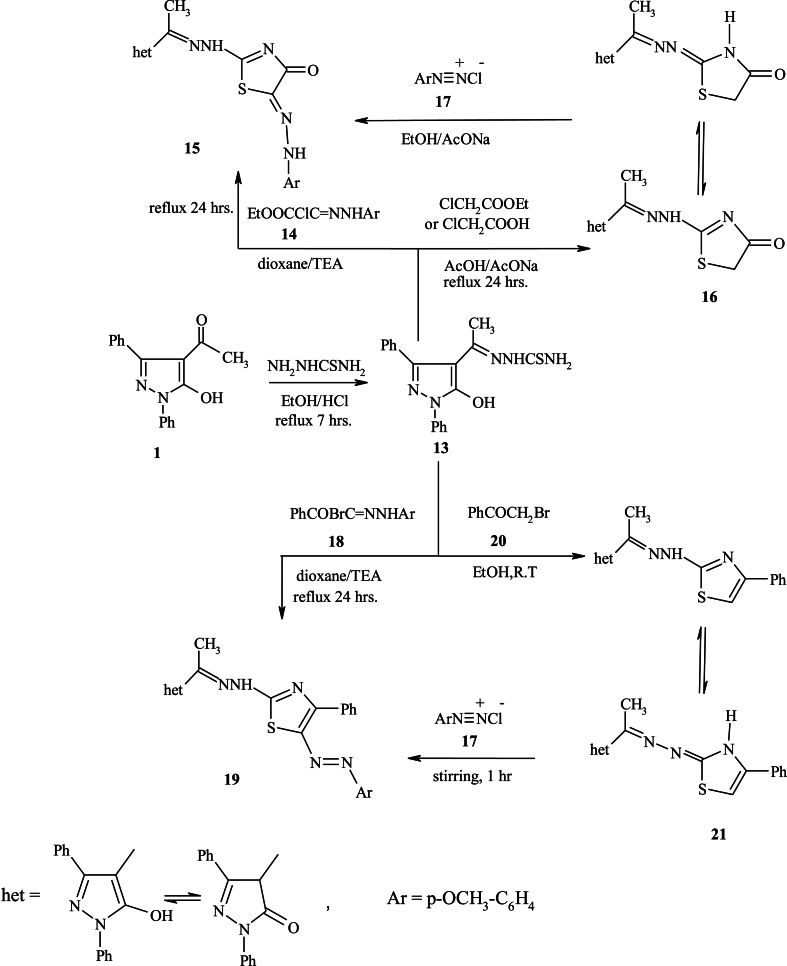
Synthetic methods for compounds **13**, **15**, **16**, **19** and **21**.

Coupling of **16** with aryldiazonium chloride **17** yielded **15** in all respects (m.p., mixed m.p., and spectral data). Similar to this, 4-(-1-(2-(5-(4-methoxyphenyl)diazenyl)-4-phenylthiazol-2-yl) hydrazono)ethyl)-1,3-diphenyl-1H-pyrazol-5-ol **19** was synthesized by reaction of **13** and hydrazonoyl halide **18**. Finally, the thiazole derivative **21** was produced in good yield by combining thiosemicarbazone derivative **13** with bromoacetophenone. Upon coupling thiazole derivative **21** with aryldiazonium chloride **17**, the compound **19** was obtained [24,39], as declared in Scheme 4. The spectral analyses in (Supplementary Material File) confirmed the chemical structures of the newly prepared molecules.

### 2.2 *In vitro* biological activity

**Fig. 2:**
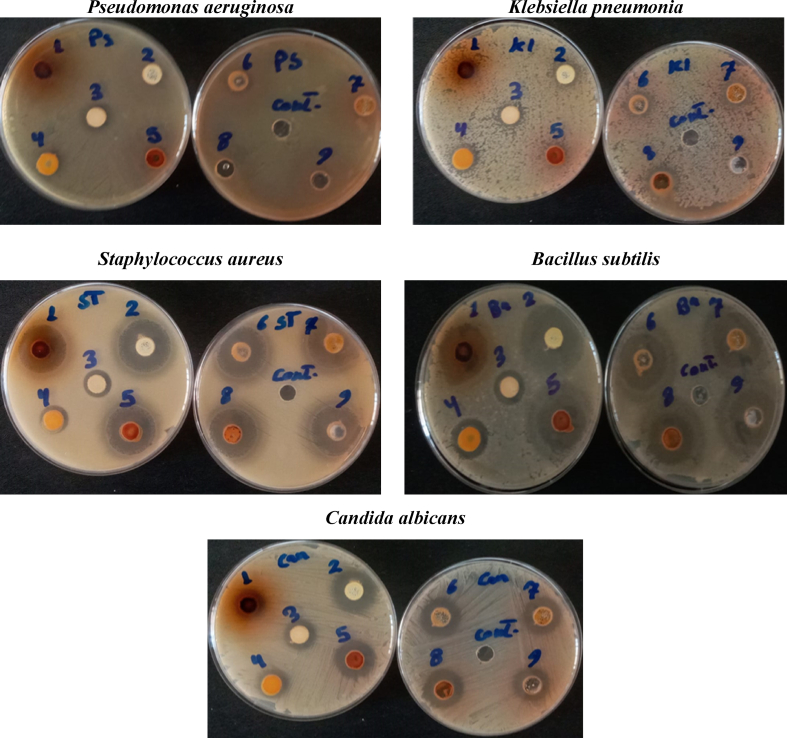
Antimicrobial activities of the prepared molecules using agar-well diffusion.

**Fig. 3:**
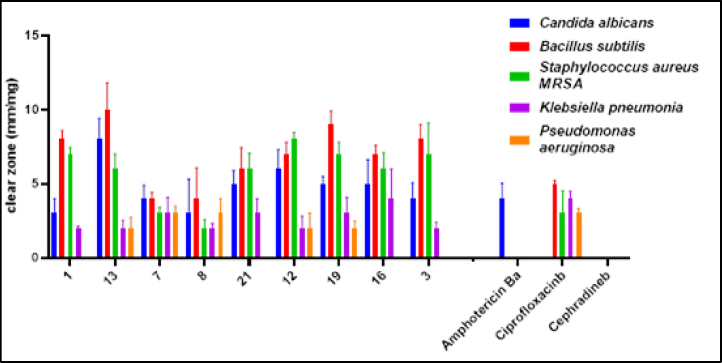
Two-way ANOVA showed a high significance between different samples and type of microbe used in the antimicrobial test. (p-value <0.0001).

The antimicrobial potency of the tested molecules was estimated against the various standard pathogen strains, gram -ive bacteria (*P. aeruginosa*, *K. pneumonia*), gram + ive bacteria (MRSA, *B. subtilis*), and fungi (*C. albicans*) microorganisms. The inhibition zone diameter was determined using agar-well diffusion and the results were stated below in (Fig. 2, Fig. 3). The results showed that most of the tested molecules exhibited moderate to high inhibition efficacy. Where **7**, **8**, **12**, **13** and **19** are the most potent compounds revealed the highest inhibition efficacy comparing to the standard drugs.

The MIC of the most potent molecules **7**, **8**, **12**, **13** and **19** was determined and mentioned in Table 1. The obtained results represented that the most potent molecules exhibited inhibition effect against most of the tested microbes at low concentration. In which compounds **12** and **13** showed strong effect against *C*. *albicans* at concentration 5 μg/mL. Compound **8** showed strong efficacy against *C*. *albicans* at concentration 10 μg/mL. Compound **8**, **12**, **13** and **19** exhibited a strong antimicrobial potency against *B*. *subtilis* at low concentration 5 μg/mL. Otherwise, compound **12** showed strong efficacy against MRSA at concentration 10 μg/mL.

**Table 1:** Minimum inhibitory concentration (MIC) of the potent prepared pyrazole derivatives.

Compound	Minimum Inhibitory Concentration (MIC, μM)				
	*C*. *albicans*	*B*. *subtilis*	*S*. *aureus MRSA*	*K*. *pneumonia*	*P*. *aeruginosa*
13	0.142	0.142	0.057	0.228	0.114
7	0.026	0.128	0.102	0.205	0.102
8	0.015	0.007	0.245	0.490	0.061
12	0.008	0.008	0.017	0.068	0.273
19	0.034	0.008	0.068	0.034	0.136
Amphotericin B^a^	40	–			
Ciprofloxacin^b^	–	20	80	40	40

**Fig. 4:**
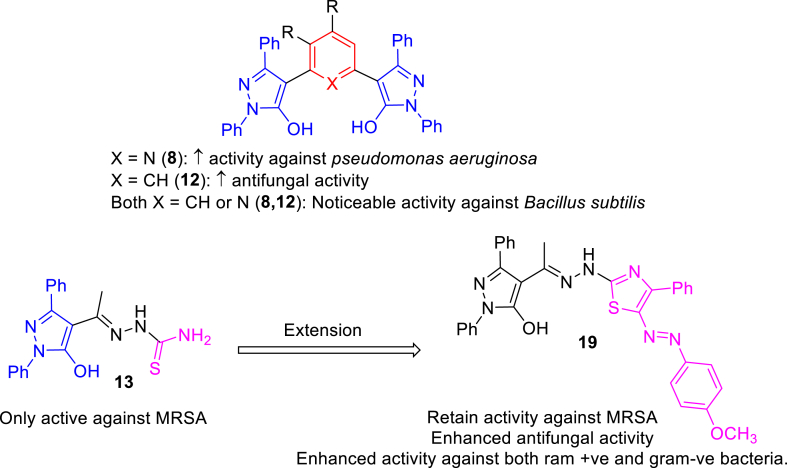
The structure activity relationship (SAR) of the molecules.

The structure activity relationship (SAR) is represented in Fig. 4. The dipyrazolylbenzene derivative **12** showed the highest antifungal activity (MIC = 8 μM). Compounds that showed the highest antibacterial activity against *B*. *subtilis* (MIC = 7–8 μM) were 2,6-dipyrazolylpyridine derivative **8**, dipyrazolylbenzene derivative **12**, and hydrazinylthiazole derivative **19**. Additionally, hydrazinylthiazole derivative **19** was the most potent against *K*. *pneumonia*. The dipyrazolylbenzene derivative **12** exhibited the highest antibacterial activity against MRSA. Finally, 2,6-dipyrazolylpyridine derivative **8** was the most potent against *P. aeruginosa*.

### 2.3 *In silico* approaches

#### 2.3.1 Molecular docking techniques

**Fig. 5:**
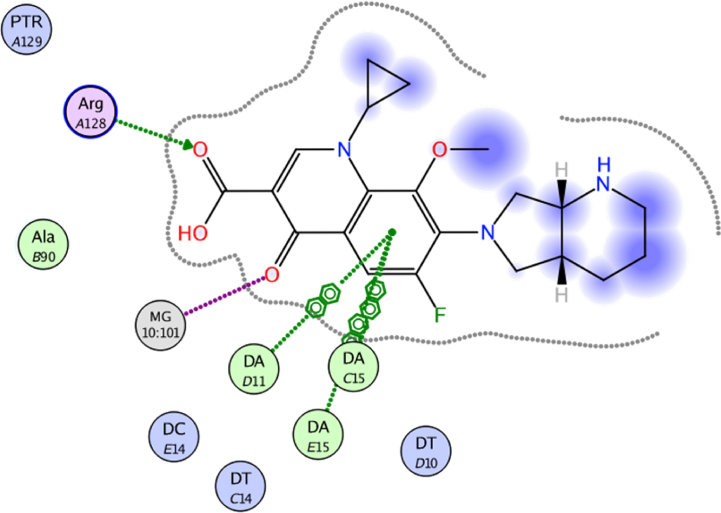
2D interaction of moxifloxacin against DNA gyrase.

**Fig. 6:**
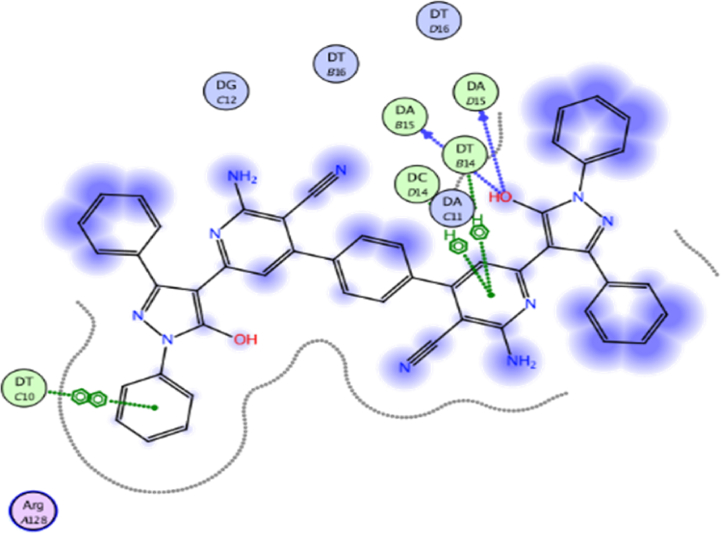
2D interaction of compound **7** against DNA gyrase.

**Fig. 7:**
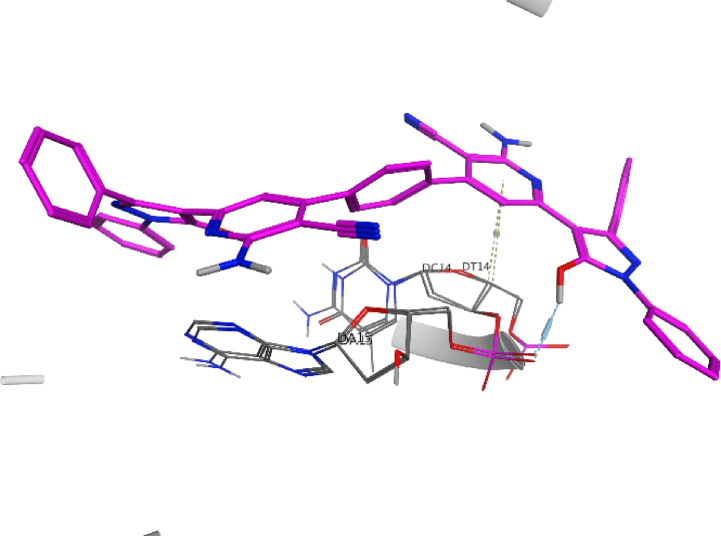
3D interaction of compound **7** against DNA gyrase.

**Fig. 8:**
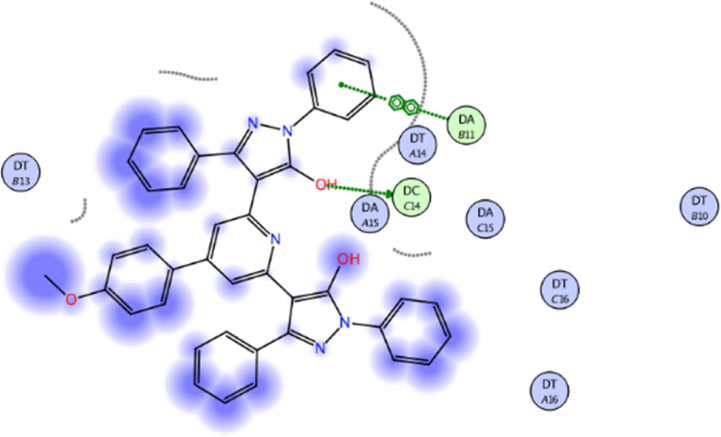
2D interaction of compound **8** against DNA gyrase.

**Fig. 9:**
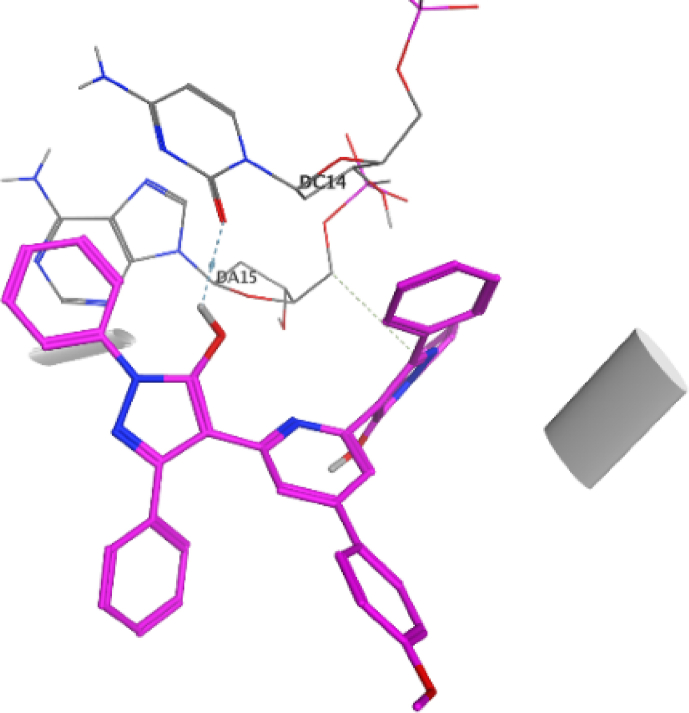
3D interaction of compound **8** against DNA gyrase.

**Fig. 10:**
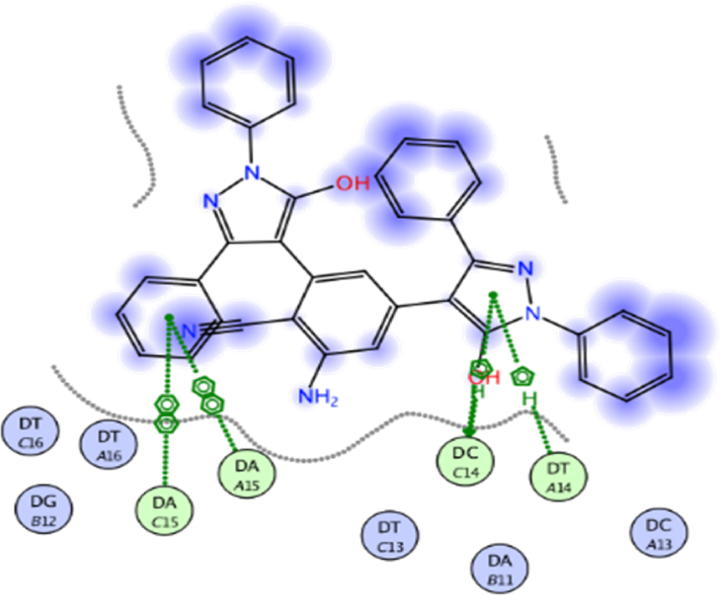
2D interaction of compound **12** against DNA gyrase.

**Fig. 11:**
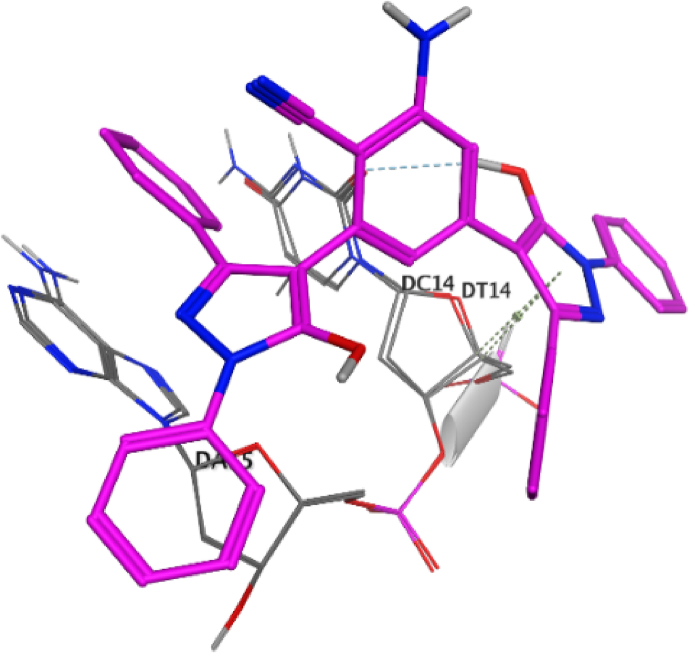
3D interaction of compound **12** against DNA gyrase.

**Fig. 12:**
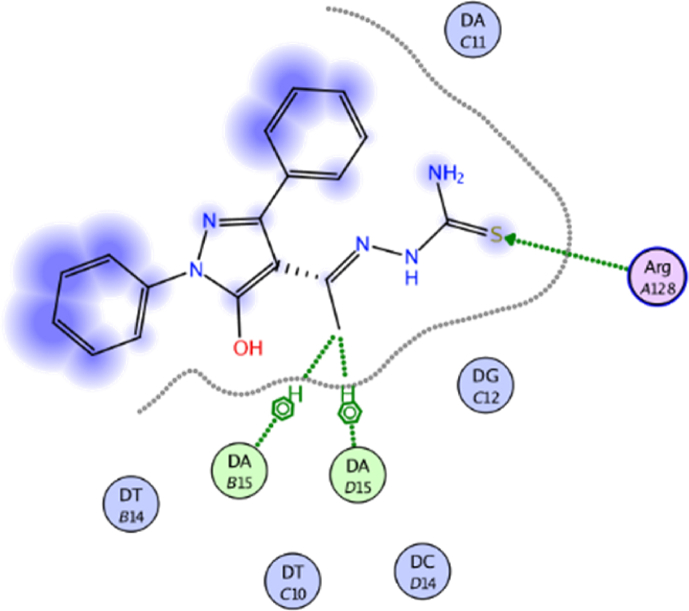
2D interaction of compound **13** against DNA gyrase.

**Fig. 13:**
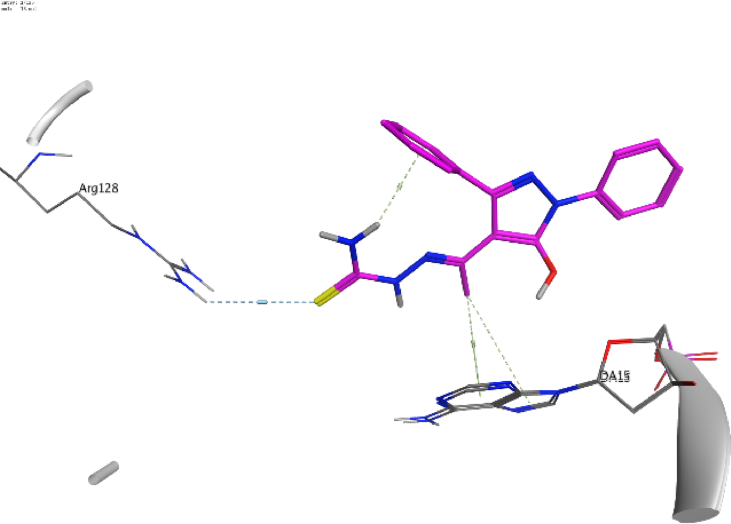
3D interaction of compound **13** against DNA gyrase.

**Fig. 14:**
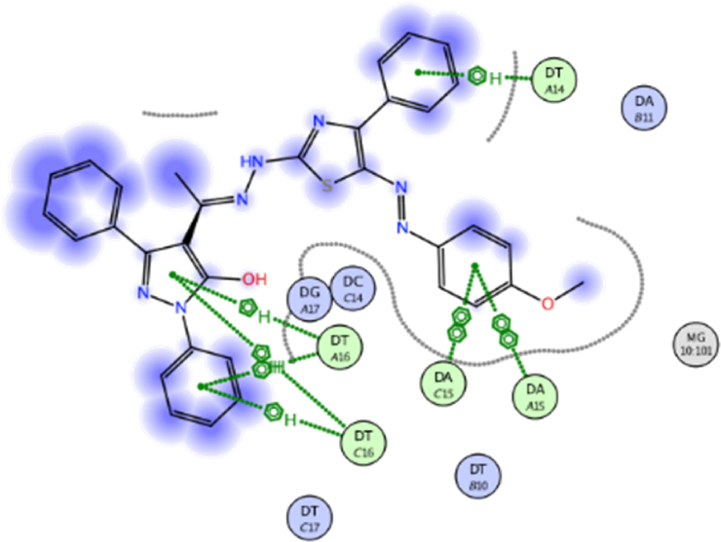
2D interaction of compound **19** against DNA gyrase.

**Fig. 15:**
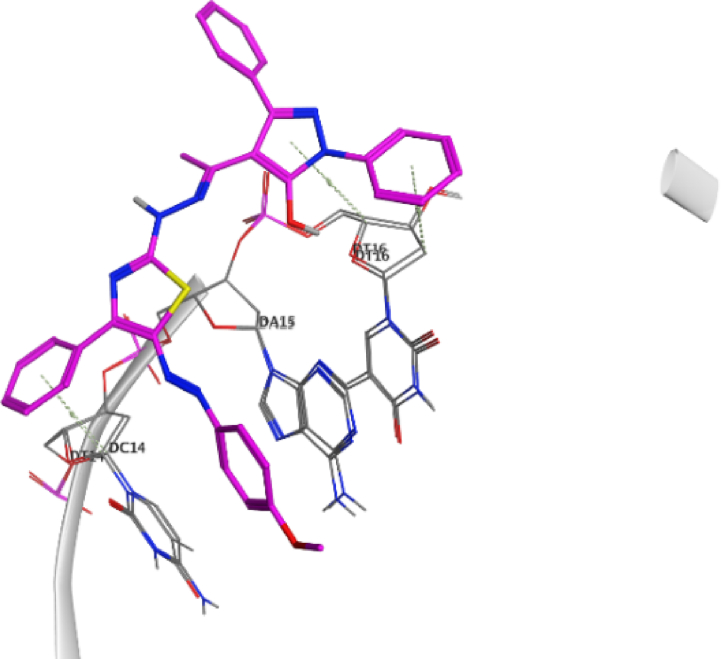
3D interaction of compound **19** against DNA gyrase.

The docking approach is utilized discover structures for the active sites of proteins, in addition to identify the potential mechanism of action [40,41]. In the present work, to go deeper into the suitable binding pose and molecular mechanism of antimicrobial activity of compounds, molecular docking studies [42] were conducted using MOE software. Molecular docking studies revealed good interactions of compounds with DNA gyrase enzyme [43], as shown in Fig. 5, Fig. 6, Fig. 7, Fig. 8, Fig. 9, Fig. 10, Fig. 11, Fig. 12, Fig. 13, Fig. 14, Fig. 15. Compared to moxifloxacin (score = −9.54 kcal/mol), Compound **7** (score = −8.29 kcal/mol) formed dual hydrogen bonds (HBs) (2.83 and 3.05 Å) with DA15 *via* hydroxyl group of pyrazole ring. The pyridine ring of compound **7** formed π-H interactions with DT14 as well as DC14 (4.16 and 4.13 Å, respectively). Additionally, phenyl ring at N1 of distal pyrazole of compound **7** formed π- π (3.89 Å) interaction with DT10 (Fig. 8).

Hydroxyl group of pyrazole ring of compound **8** (score = −10.07 kcal/mol) acted as hydrogen bond donor (HBD) with DC14 (3.07 Å) (Fig. 9). In addition, N1-phenyl of pyrazole of compound **8** formed π- π interaction (3.59 Å) with DA11. Compound **12** (score = −8.06 kcal/mol) formed HB (3.42 Å) with DC14 (Fig. 10). The good affinity of compound **8** toward DNA gyrase enzyme (score = −10.07 kcal/mol) is reflected in its good binding strength against *B*. *subtilis* (MIC = 0.007 μg/mL) and *P*. *aeruginosa* (MIC = 0.061 μg/mL).

Moreover, pyrazole ring of compound **12** formed two π-H interactions (3.81 and 3.76 Å) with DT14 and DC14, respectively. In addition, the phenyl ring at N1 of distal pyrazole of compound **12** formed two π- π interactions (3.76 and 3.71 Å) with DA15.

Compound **13** (score = −7.46 kcal/mol) acted as hydrogen bond acceptor (HBA) (4.49 Å) *via* hydrazine carbothioamide moiety (Fig. 11) as well as forming H-π interactions (3.99 and 4.06 Å) with DA15.

Finally, *N*-phenylpyrazole moiety of compound **19** (score = −12.01 kcal/mol) formed four π-H interactions (4.33, 3.82, 4.37 and 3.85 Å) with DT16 (Fig. 12). Phenyl ring at thiazole C4 of compound **19** formed π-H interactions (3.89 Å) with DT14. Additionally, phenyldiazenyl mioety at thaizole C5 formed two π- π interactions (3.84 and 3.83 Å) with DA15. Out of all synthesized derivatives, compound **19** showed the highest binding score which aligns with its good binding strength against MRSA (MIC = 0.068 μg/mL), *B*. *subtilis* (MIC = 0.008 μg/mL) and *K*. *pneumonia* (MIC = 0.034 μg/mL).

#### 2.3.2 Pharmacokinetics prediction

Aside from efficacy and safety profiles, several drug candidates have failed to reach clinic due to their poor physicochemical characters and pharmacokinetics [26,27]. SwissADME website was utilized to estimate several physicochemical and pharmacokinetic properties of molecules (Supplementary Material). None of the compounds was predicted to be P-gp substrate. Compounds were predicted to have a little inhibitory activity on different CYP450 isozymes like 1A2, 2C19, 2C9, 2D6 and 3A4. Consequently, compounds were predicted to have few drug-drug interactions. Compounds **1**, **3**, **5**, **10**, **13**, **15**, **16** and **19** showed high GI absorption while other compounds **7**, **8**, **9**, **11**, **12** and **21** showed low GI absorption.

The BOILED-Egg is a sturdy model that precisely predicts both GI absorption and blood brain barrier (BBB) permeability by calculating both the WLOGP (lipophilicity) and TPSA (polarity) (Supplementary Material). Compounds **1, 3, 5, 10, 13** and **16** were predicted to have a high GI absorption while compounds **9, 11, 12**, **15** and **21** were predicted to have a low GI absorption. Except compound **1**, all compounds were predicted not to pass BBB, indicating their good CNS safety profile.

There are 6 physicochemical characters that were considered in “bioavailability radar” viz, solubility, polarity, lipophilicity, saturation, flexibility, and size, which construct together a hexagon shape (Supplementary Material). The inner pink colored hexagon indicates the optimal values for acceptable bioavailability. The molecular weights of compounds **1**, **3**, **5**, **10**, **13**, **16** and **21** are below 500 g/mol while compounds **7**, **8**, **9**, **11, 12**, **15** and **19** have molecular weights above 500 g/mol. Consequently, compounds **1**, **3**, **5**, **10**, **13**, **15**, **16**, **19** and **21** have satisfied Lipinski rule without any violation. Compounds **1**, **3**, **4**, **10**, **13**, **15**, **16** and **21** have acceptable Fraction Csp3 (>0.03) that improves instauration parameter. All compounds have rotatable bonds within the allowed range (<8 bond) that enhance their flexibility. To conclude, we can say that compounds **1**, **3**, **4**, **10**, **13**, **15**, **16** and **21** are predicted to have acceptable bioavailability.

## 3 Materials and methods

### 3.1 Chemistry

Kofler Block instrument was utilized to determine the melting points of the prepared compounds. The FTIR 5300 spectrometer (*ν*, cm^−1^) was used to record IR spectra. In addition, ^1^H and ^13^C NMR spectra were performed by a Varian Gemini spectrometer (400 and 100 MHz, respectively) in DMSO‑*d*_6_ as solvents. Tetramethylsilane (TMS), was used as an internal reference, is used to express the chemical changes in parts per million (ppm). 1000 EX mass spectrometer at 70 eV. Using n-hexane and EtOAc, thin layer chromatography (TLC) on aluminum sheets was used to determine the purity of the produced compounds. The elemental analyses were carried out at Microanalytical Research Center, Faculty of Science, Cairo University, Egypt.

#### 3.1.1 2-(1-(5-hydroxy-1,3-diphenyl-1H-pyrazol-4-yl)ethylidene)-5,5-dimethylcyclohexane-1,3-dione (3)

Dimedone (0.01 mol) and **1** (0.01 mol) were combined and refluxed for 24 h in EtONa (30 mL), then washed with ice/water, let to cool, and acidified with HCl. The formed solid was collected by filtration, and recrystallized from ethanol to yield (**3**; 66 %) as yellowish brown crystals, m.p.140–142 °C; IR (KBr) *ν* cm^−1^ = 3447 (OH), 3063 (CH-_aromatic_), 2925–2807 (CH-_aliphatic_), 1712 (C=O); ^1^H NMR (400 MHz, DMSO‑*d*_6_) *δ* = 1.30 (s, 3H, CH_3_), 1.56 (s, 6H, 2CH_3_), 3.04 (s, 2H, CH_2_), 3.34 (s, 2H, CH_2_), 7.21–7.92 (m, 10H, Ar–H), 11.39 (hump, 1H, OH); ^13^C NMR (100 MHz, DMSO‑*d*_6_) *δ* = 23.20, 28.25, 33.50, 42.82, 85.65, 121.58, 125.56, 126.13, 126.13, 128.33, 129.02, 129.37, 131.02, 133.82, 139.32, 150.08, 154.32, 171.53, 194.40; MS = *m/z (%)* 401 (M^+^+1). Anal. calcd. for C_25_H_24_N_2_O_3_ (400): C, 74.98; H, 6.04; N, 7.00; Found: C, 74.12; H, 6.08; N, 7.03 %.

#### 3.1.2 2-Amino-4-(5-hydroxy-1,3-diphenyl-1H-pyrazol-4-yl)-4,7,7-trimethyl-5-oxo-5,6,7,8-tetrahydro-4H-chromene-3-carbonitrile (5)

In the presence of EtONa (30 mL), malononitrile (0.01 mol) was added to compound **3** (0.01 mol). For 30 h, the reaction mixture was heated under reflux, washed with ice-cold water, then acidified with HCl. The formed solid was collected by filtration, dried, and recrystallized from ethanol to produce (**5**; 61 %) as yellowish-brown crystals, m.p.110–112 °C; IR = 3449 (OH), 3400 (NH_2_), 3062 (CH-_aromatic_), 2956–2926 (CH-_aliphatic_), 2198 (CN), 1710 (C=O); ^1^H NMR = 0.94 (s, 6H, 2CH_3_), 2.19 (s, 3H, CH_3_), 3.51 (s, 2H, CH_2_), 3.62 (s, 2H, CH_2_), 6.02 (s, 2H, NH_2_), 7.29–7.83 (m, 10H, Ar–H), 14.49 (s, 1H, OH); ^13^C NMR = 19.60, 23.02, 28.25, 28.25, 33.50, 42.62, 54.66, 116.86, 118.83, 121.60, 125.55, 126.16, 128.35, 129.03, 129.38, 129.46, 131.02, 133.76, 139.27, 150.09, 154.44, 162.01, 199.9; MS = *m/z (%)* 466 (M^+^). Anal. calcd. for C_28_H_26_N_4_O_3_ (466): C, 72.09; H, 5.62; N, 12.01; Found: C, 72.14; H, 5.67; N, 12.05 %.

#### 3.1.3 4,4'-(1,4-phenylene)bis(2-amino-6-(5-hydroxy-1,3-diphenyl-1H-pyrazol-4-yl)nicotinonitrile) (7)

**Method A:** A solution of **1** (0.02 mol), malononitrile (0.02 mol), and terephthaldehyde (0.01 mol) in glacial AcOH (15 mL) and ammonium acetate (2 g). The solution was heated for 24 h at reflux while being stirred. The finished solution was cooled before being poured over crushed ice. The solid formed was filtered off, then recrystallized from dioxane to give (**7**; 74 %**)**. **Method B:** A combined solution of **1** (0.02 mol) and 2,2'-(1,4-phenylenebis(methan-1-yl-1-ylidene))dimalononitrilein **6** (0.01 mol). The solution was heated for 24 h while being stirred under reflux. The product obtained was treated with ice-cold water, filtered, dried and recrystallized from dioxane to furnish (**7**; 74 %) as yellow crystals; m.p. 268–270 °C; IR = 3461 (OH), 3328 (NH_2_), 3058 (CH-_aromatic_), 2195 (CN); ^1^H NMR = 5.24 (s, 2H, 5H-Pyridine), 6.10 (s, 4H, 2NH_2_), 7.12–7.90 (m, 24, Ar–H), 14.49 (hump, 2H, 2OH); ^13^C NMR = 98.06, 106.02, 111.01, 121.28, 121.87, 125.55, 126.93, 127.33, 128.67, 129.42, 129.88, 131.33, 132.40, 137.80, 145.61, 146.80, 154.40, 159.16, 160.01; MS: *m/z (%)* 780 (M^+^). Anal. calcd. for C_48_H_32_N_10_O_2_ (780): C, 73.83; H, 4.13; N, 17.94; Found: C, 73.88; H, 4.17; N, 17.99 %.

#### 3.1.4 4,4'-(4-(4-methoxyphenyl)pyridine-2,6-diyl)bis(1,3-diphenyl-1H-pyrazol-5-ol) (8)

A solution of glacial AcOH (15 mL) containing ammonium acetate (2 g) was mixed with a suspension of **1** (0.02 mol) and *p*-methoxybenzaldhyde (0.01 mol). The mixture was heated under reflux at 160 °C for 24 h while being stirred, then treated with ice-cold water. The solid product was collected by filtration, and dried then recrystallized from ethanol to furnish (**8**; 66 %) as yellow powder; m.p.128–130 °C. IR = 3449 (OH), 3060 (CH-_aromatic_), 2931 (CH-_aliphatic_); ^1^H NMR = 3.71 (s, 3H, OCH_3_), 5.23 (s, 2H, 3-H and 5-H of pyridine ring), 6.87–7.84 (m, 24H, Ar–H), 14.32 (s, 2H, 2OH); ^13^C NMR = 55.44, 106.96, 114.32, 115.32, 121.79, 126.72, 128.40, 128.58, 129.18, 129.18, 129.46, 133.86, 137.66, 145.80, 149.49, 152.03, 158.17, 165.01; MS: *m/z %* 653 (M^+^). Anal. calcd for C_42_H_31_N_5_O_3_ (653): C, 77.17; H, 4.78; N, 10.71; Found: C, 77.24; H, 5.82; N, 10.73 %.

#### 3.1.5 1,3-bis(5-hydroxy-1,3-diphenyl-1H-pyrazol-4-yl)but-2-en-1-one (9)

A molar equivalent of **1** (0.02 mol) in 30 mL of EtONa. For 3 h, the mixture was refluxed, then left to cool. The mixture was poured into ice-cold water, and acidified with HCl. The solid formed was collected by filtration, dried and recrystallized from ethanol to give the desired product (**9**; 49 %) as yellow powder; m.p.130–132 °C; IR = 3450 (OH), 3059 (CH-_aromatic_), 2955 (CH-_aliphatic_), 1710 (C=O); ^1^H NMR = 1.90 (s, 3H, CH_3_), 5.28 (s, 1H, CH-olefinic), 6.89–7.87 (m, 20, Ar–H), 14.38 (hump, 2H, 2 OH); MS = *m/z (%)* 538 (M^+^). Anal. calcd. for C_34_H_26_N_4_O_3_ (538): C, 75.82; H, 4.87; N, 10.40; Found: C, 75.89; H, 4.92; N, 10.42 %.

#### 3.1.6 2-(1-(5-hydroxy-1,3-diphenyl-1H-pyrazol-4-yl)ethylidene)malononitrile (10)

In the presence of EtONa (30 mL), malononitrile (0.01 mol) was added to **1** (0.01 mol). For 1 h, the mixture was refluxed, then left to cool before being treated with crushed ice and acidified with HCl. The solid formed was collected by filtration, and recrystallized from ethanol to yield (**10**; 66 %) as yellow crystals; m.p.164–166 °C; IR = 3450 (OH), 3063 (CH-_aromatic_), 2955 (CH-_aliphatic_), 2192 (CN); ^1^H NMR = 2.40 (s, 3H, CH_3_), 7.27–7.84 (m, 10H, Ar–H), 10.50 (s, 1H, OH); MS = *m/z (%)* 326 (M^+^). Anal. calcd. For C_20_H_14_N_4_O (326): C, 73.61; H, 4.32; N, 17.17; Found: C, 73.64; H, 4.36; N, 17.23 %.

#### 3.1.7 2-Amino-4,6-bis(5-hydroxy-1,3-diphenyl-1H-pyrazol-4-yl)benzonitrile (12)

**Method A:** In the presence of EtONa (30 mL), malononitrile (0.01 mol) was added to **9** (0.01 mol). For 24 h, the solution was refluxed. Once it had stopped, it was treated with ice-cold water then acidified with HCl. The solid formed was collected and recrystallized from ethanol to furnish (**12**; 66 %). **Method B:** compounds **1** (0.01 mol) and **10** (0.01 mol) were mixed together in (30 mL) of EtONa. For 24 h, the solution was heated under reflux. After completion the reaction, a cold diluted HCl was added to the reaction mixture, and the solid precipitated was filtered off, and recrystallized from ethanol to produce (**12**; 68 %) as yellow crystals; m.p.202–204 °C; IR = 3387 (OH), 3302 (NH_2_), 3059 (CH-_aromatic_), 2206 (CN); ^1^H NMR = 6.03 (s, 2H, NH_2_), 7.27–7.84 (m, 22H, Ar–H), 11.79 (s, 2H, 2OH); ^13^C NMR = 85.27, 114.90, 116.10, 118.37, 122.51, 125.16, 127.85, 128.54, 128.54, 128.83, 128.89, 133.35, 138.36, 142,00 143.80, 145.63, 149.63, 153.97; MS = *m/z (%)* 586 (M^+^). Anal. calcd. for C_37_H_26_N_6_O_2_ (586): C, 75.75; H, 4.47; N, 14.33; Found: C, 75.78; H, 4.50; N, 14.36 %.

#### 3.1.8 2-(1-(5-hydroxy-1,3-diphenyl-1H-pyrazol-4-yl)ethylidene)hydrazinecarbothioamide (13)

Thiosemicarbazide (0.01 mol) and **1** (0.01 mol) were refluxed for 7 h in (20 mL) absolute ethanol with 2 drops of HCl. After completion the reaction, the solid precipitated was collected by filtration and recrystallized from ethanol to yield (**13**; 75 %) as yellowish-white crystals; m.p.170–172 °C. IR = 3449 (OH), 3300 (NH_2_), 3062 (CH-_aromatic_), 2833 (CH-_aliphatic_); ^1^H NMR = 1.84 (s, 3H, CH_3_), 6.04 (s, 2H, NH_2_) 7.22–7.85 (m, 10H, Ar–H), 8.66 (s, 1H, NH), 11.60 (hump, 1H, OH); MS = *m/z (%)* 353 (M^+^+2). Anal. calcd. for C_18_H_17_N_5_OS (351): C, 61.52; H, 4.88; N, 19.93; Found**:** C, 61.59; H, 4.92; N, 19.97 %**.**

#### 3.1.9 2-(1-(5-hydroxy-1,3-diphenyl-1H-pyrazol-4-yl)ethylidene)hydrazinyl)-5-(2-(4-methoxyphenyl) hydrazono)thiazol-4(5H)-one (15)

**Method A:** In dioxane (30 mL) containing 3 drops of TEA, a suspension of **13** (0.01 mol) and ethyl 2-chloro-2-(2-(4-methoxyphenyl) hydrazono)acetate **14** (0.01 mol) was refluxed for 24 h. After allowing the solution to stop, a cold diluted HCl was added to the mixture. The solid formed was filtered off, dried, then recrystallized from ethanol to afford (**15**; 75 %). **Method B:** Aryldiazonium chloride **17** was added dropwise to a cold solution of **16** (0.01 mol) in ethanol (20 mL) containing excess of sodium acetate (2 gm). After completion the addition, the reaction mixture was allowed to stirrer for further 1 h in ice bath, then left overnight. The solid formed was collected by filtration, and recrystallized from ethanol to yield (**15**; 82 %) as yellowish brown crystals, m.p.120–122 °C. IR = 3300 (OH), 3128 (NH), 3028 (CH-_aromatic_), 2881 (CH-_aliphatic_), 1685 (C=O); ^1^H NMR = 1.24 (s, 3H, CH_3_), 3.80 (s, 3H, OCH_3_). 6.02 (s, 1H, NH), 6.50 (s, 1H, NH), 7.25–8.05 (m, 14H, Ar–H), 14.90 (s, 1H, OH); MS = *m/z (%)* 527 (M^+^+2). Anal. calcd. for C_27_H_23_N_7_O_3_S (525): C, 61.70; H, 4.41; N, 18.65; Found**:** C, 61.75; H, 4.46; N, 18.70 %.

#### 3.1.10 2-(2-(1-(5-hydroxy-1,3-diphenyl-1H-pyrazol-4-yl)ethylidene)hydrazinyl)thiazol-4(5H)-one (16)

In a round flask containing glacial AcOH (10 mL) and sodium acetate (1 g), a suspension of **13** (0.01 mol) and chloroacetic acid and/or ethyl chloroacetate (0.01 mol) was added, and the reaction mixture was refluxed for 24 h. After completion the reaction (monitored by TLC), a cold diluted HCl was added to the mixture. The solid precipitated was collected by filtration, washed with water and crystallized from ethanol (**16**; 75 %) as yellow crystals, m.p.120–122 °C; IR = 3450 (OH), 3052 (CH-_aromatic_), 2955 (CH-_aliphatic_), 1709 (C=O); ^1^H NMR = 1.25 (s, 3H, CH_3_), 5.02 (s, 2H, CH_2_), 6.06 (s, 1H, NH), 7.28–7.86 (m, 10H, Ar–H), 11.80 (hump, 1H, OH); ^13^C NMR = 14.01, 60.21, 85.05, 121.7, 126.12, 127.83, 127.83, 128.91, 129.01, 129.01, 129.07, 129.07, 133.36, 138.87, 153.95, 154.39, 163.02, 167.70, 170.01; MS = *m/z (%)* 393(M^+^+2). Anal. calcd. for C_20_H_17_N_5_O_2_S (391): C, 61.37; H, 4.38; N, 17.89; Found**:** C, 61.40; H, 4.41; N, 17.92 %.

#### 3.1.11 4-(-1-(5-(4-methoxyphenyl)diazenyl)-4-phenylthiazol-2(3H)-ylidene)hydrazono)ethyl)-1,3-diphenyl-1H-pyrazol-5-ol (19)

**Method A:** A mixture of **13** (0.01 mol) and N'-(4-methoxyphenyl)-2-oxo-2-phenylaceto hydrazonoyl bromide **18** (0.01 mol) in dioxane (20 mL) and 3 drops of TEA was heated under reflux for 24 h. After completion the reaction (monitored by TLC), a cold diluted HCl was added to the mixture. The solid formed was collected by filtration, then recrystallized from ethanol to give (**19**; 75 %). **Method B:** A portion of the aryldiazonium chloride **17** solution was added dropwise to a cold solution of **21** (0.01 mol) in ethanol (20 mL) and excess of sodium acetate (2 g). The mixture was allowed to stirrer for 1 h in an ice bath. After completion the addition, the solid precipitated was filtered off, and recrystallized from ethanol to yield (**19**; 82 %) as yellow crystals, m.p. 240–242 °C. IR = 3392 (OH), 3300 (NH), 3056 (CH-_aromatic_), 2955 (CH-_aliphatic_); ^1^H NMR = 1.20 (s, 3H, CH_3_), 3.79 (s, 3H, OCH_3_), 6.20 (s, 1H, NH), 7.06–8.19 (m, 19H, Ar–H), 13.86 (hump, 1H, OH); ^13^C NMR = 20.47, 55.89, 85.34, 93.98, 118.40, 118.40, 121.14, 122.25, 122.25, 126.14, 127.47, 127.47, 127.87, 128.33, 128.33, 128.33, 128.56, 128.56, 128.81, 128.81, 128.9, 128.9, 129.41, 129.41, 132.69, 133.4, 137.67, 144.96, 149.69, 149.98, 154.05, 166.63, 172.70; MS = *m/z (%)* 587 (M^+^+2). Anal. calcd. for C_33_H_27_N_7_O_2_S (585): C, 67.67; H, 4.65; N, 16.74; Found**:** C, 67.70; H, 4.68; N, 16.77 %.

#### 3.1.12 1,3-Diphenyl-4-(1-(2-(4-phenylthiazol-2-yl)hydrazono)ethyl)-1H-pyrazol-5-ol (21)

A mixture of **13** (0.01 mol) and phenacyl bromide **20** (0.01 mol) was dissolved in absolute ethanol (20 mL). The reaction mixture was stirred at RT for 2 h before being refluxed for 20 h. After completion the reaction, ice-cold water was added to the mixture. The solid formed was collected by filtration, and recrystallized from ethanol to afford the desired product (**21**; 75 %) as brown crystals, m.p.100–102 °C. IR = 3450 (OH), 3400 (NH), 3058 (CH-_aromatic_), 2955 (CH-_aliphatic_); ^1^H NMR = 1.10 (s, 3H, CH_3_), 6.04 (s, 1H, NH), 7.27–7.84 (m, 16H, Ar–H), 14.57 (s, 1H, OH); MS = *m/z (%)* 453 (M^+^+2). Anal. calcd. for C_26_H_21_N_5_OS (451): C, 68.85; H, 5.11; N, 15.44; Found: C, 68.89; H, 5.15; N, 15.48 %.

### 3.2 Antimicrobial efficacy of the tested compounds

The ability of the molecules to prevent the microbial growth was investigated against the standard pathogen strains, gram -ive bacteria (*P*. *aeruginosa*, *K*. *pneumonia*), gram + ive bacteria (*S*. *aureus*, *B*. *subtilis*), and Unicellular fungi (*C*. *albicans*). Pre-activation of pathogens were performed by inoculating in the Nutrient broth medium for 24 h at 37 °C for bacterial strains, while fungal pathogen was inoculating in Potato Dextrose Broth (PDB) medium for 48 h at 28 °C under shaking condition. Screening of the tested material with different concentrations was preliminary occur at a constant concentration of the tested compounds using turbidometry method for each microbial pathogen except for multicellular fungi, which was evaluated by the Colony Forming Unite (CFU). In addition, the evaluation of tested compounds to inhibition the microbial proliferation was assessed using agar well diffusion method in terms of the inhibition zone diameter (mm) [44,45].

#### 3.2.1 Determination of minimum inhibition concentration (MIC)

The MIC of highly potent molecules were performed to estimate the lowest concentration that inhibit the visible microbial growth after 24 h applying microdilution assay technique according to CLSI [46,47]. In the experiment, different concentrations of the tested molecules were investigated in comparison to the classical antimicrobial agents. Briefly, the tested pathogens were cultivated in Mueller Hinton Broth (MHB) at 37 °C for 24 h, then the growth was diluted with sterilized bi-distilled water corresponding to 2 × 10^7^ Colony Forming Unit (CFU)/mL, and the prepared cultures were diluted 10-folds in MHB for used as inoculum. The selected molecules were added into 100 μL aliquots into MHB, which were sequentially dispensed into the microdilution (96-well microtiter plate) in order to determine a known concentrations ranging from 5 to 200 μg/ml. A known inoculum of the bacterial cells was then incorporated with 100 μL to each well in triplicate trails. The positive control using three antibacterial agents was adjusted with the same concentrations of the tested samples [24,48]. The MHB mixed with DMSO without and with compounds were also served as the control samples.

### 3.3 Molecular docking assessment

Modeling simulations that included docking study of the synthesized compounds were achieved. At first, the native ligand, moxifloxacin, was redocked with the active region of target protein (PDB ID: 5BS8) to validate our docking methodology (RMSD = 0.9812) [49]. After that a library of 2D structures of the prepared derivatives and moxifloxacin were sketched using ChemDraw Professional 16.0, then converted to mol format. The energies of compounds and moxifloxacin were minimized and organized. The 3D structure of the target protein was prepared by removing solvent molecules and bound substances (ligands and cofactors). Docking process was conducted by default setting of MOE software [40]. SwissADME website [50] was utilized to estimate several physicochemical and pharmacokinetic properties of molecules.

## 4 Conclusion

Two hybrid series of pyrazole-clubbed pyrimidine and pyrazole-clubbed thiazole compounds were prepared and *in vitro* screened for their antimicrobial activities against various standard pathogen strains. Moreover, the docking approach was achieved for understanding the antimicrobial efficacy in terms of binding affinity and intermolecular interactions with DNA gyrase enzyme. The findings revealed that compounds **7**, **8**, **12**, **13** and **19** are considered as hopeful antimicrobial agents and could be the lead compounds for potential drug candidates.

## Data availability

All data generated or analysed during this study are included in the Supplementary Material File.

## CRediT authorship contribution statement

**Mohamed A.M. Abdel Reheim:** Writing – review & editing, Writing – original draft, Supervision. **Ibrahim S. Abdel Hafiz:** Writing – original draft, Supervision. **Hala M. Reffat:** Writing – original draft, Supervision. **Hend S. Abdel Rady:** Writing – original draft, Methodology, Formal analysis. **Ihsan A. Shehadi:** Formal analysis, Data curation. **Huda R.M. Rashdan:** Writing – original draft, Formal analysis. **Abdelfattah Hassan:** Writing – original draft, Software. **Aboubakr H. Abdelmonsef:** Writing – review & editing, Writing – original draft, Supervision, Formal analysis.

## Declaration of competing interest

The authors declare that they have no known competing financial interests or personal relationships that could have appeared to influence the work reported in this paper.
