# Supplementary material for: New 1,3-diphenyl-1H-pyrazol-5-ols as anti-methicillin resistant Staphylococcus aureus agents: Synthesis, antimicrobial evaluation and in silico studies
Source: Heliyon. 2024 Jun 25;10(13):e33160. doi: 10.1016/j.heliyon.2024.e33160 (PMC11259802; doi:10.1016/j.heliyon.2024.e33160)
Supplement: Multimedia component 1 [file mmc1.docx]

**New 1,3-Diphenyl-1*H*-pyrazol-5-ols as Anti-Methicillin Resistant *Staphylococcus aureus* Agents: Synthesis, Antimicrobial Evaluation and *In Silico* Studies**

Mohamed A. M. Abdel Reheim^1^, Ibrahim S. Abdel Hafiz^1^, Hala M. Reffat^1^, Hend S. Abdel Rady^1^, Ihsan A. Shehadi^2^, Huda R. M. Rashdan^3^, Abdelfattah Hassan^4^, Aboubakr H. Abdelmonsef^5,*^

^1^Department of Chemistry, Faculty of Science, Arish University, Arish 45511, Egypt

^2^Chemistry Department, College of Sciences, University of Sharjah, Sharjah 27272, United Arab Emirates

^3^Chemistry of Natural and Microbial Products Department, Pharmaceutical and Drug Industries Research Institute, National Research Centre, 33 El Buhouth St, Dokki, Giza 12622, Egypt

^4^Department of Medicinal Chemistry, Faculty of Pharmacy, South Valley University, Qena 83523, Egypt

^5,^*Department of Chemistry, Faculty of Science, South Valley University, Qena 83523, Egypt


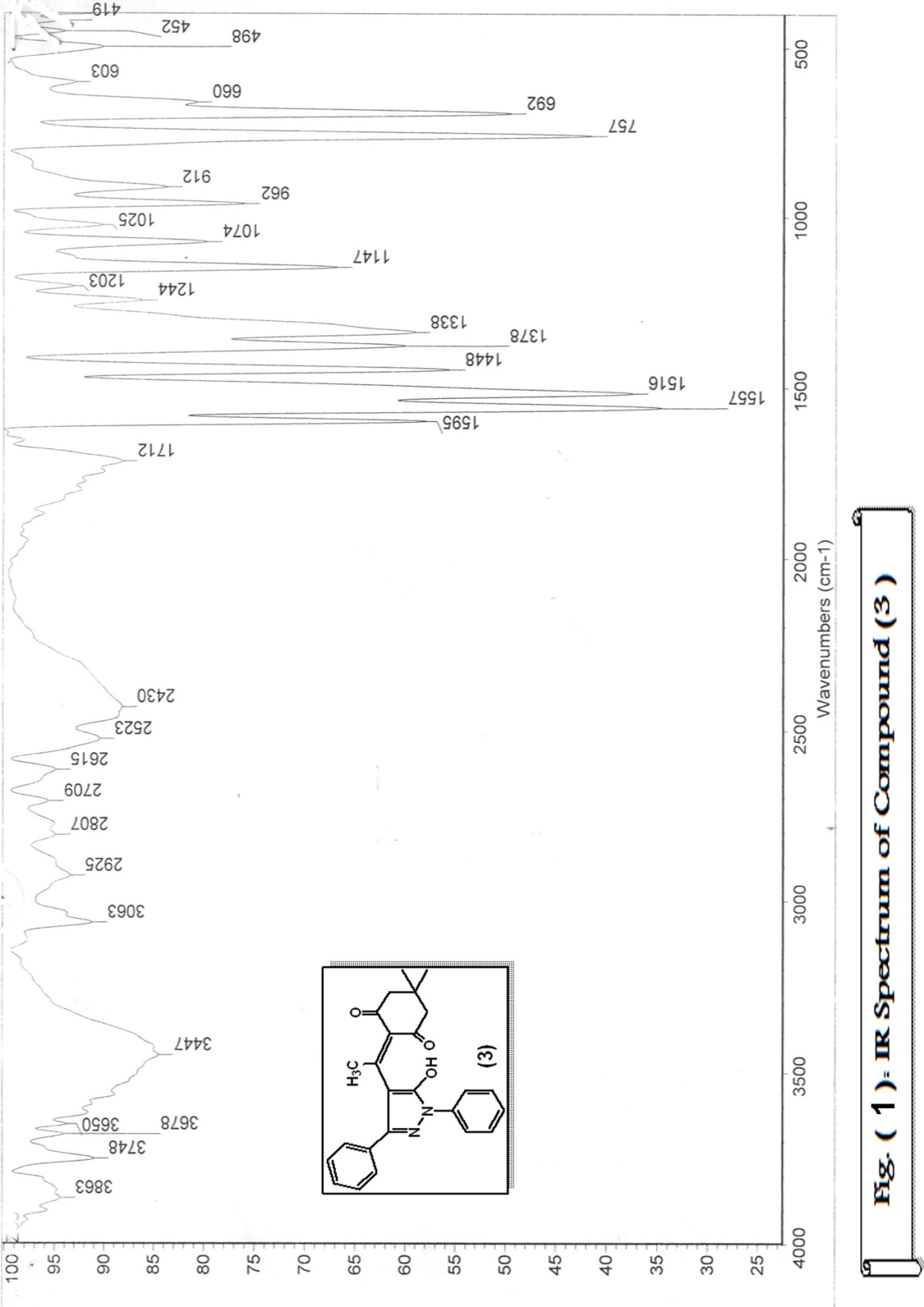


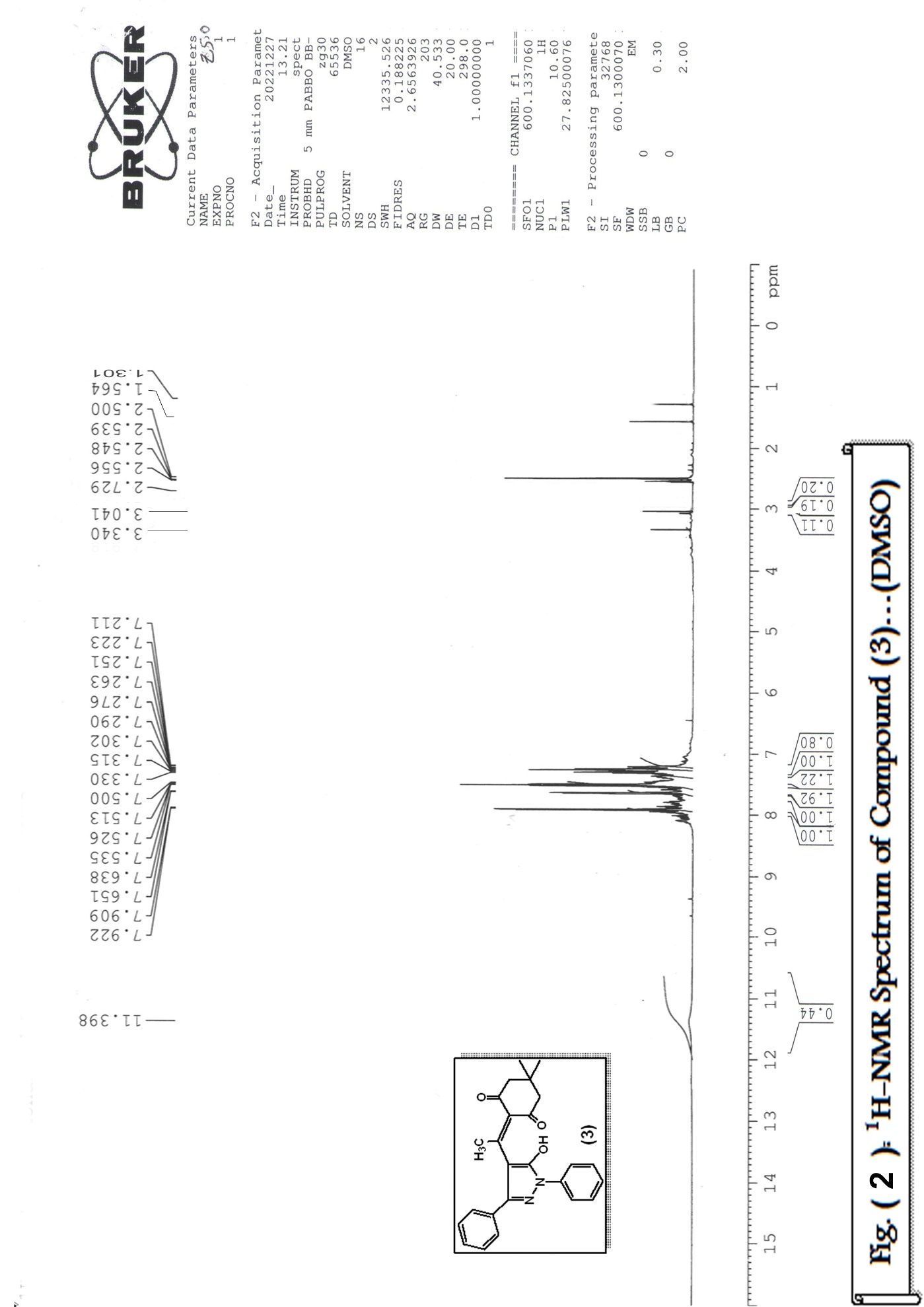


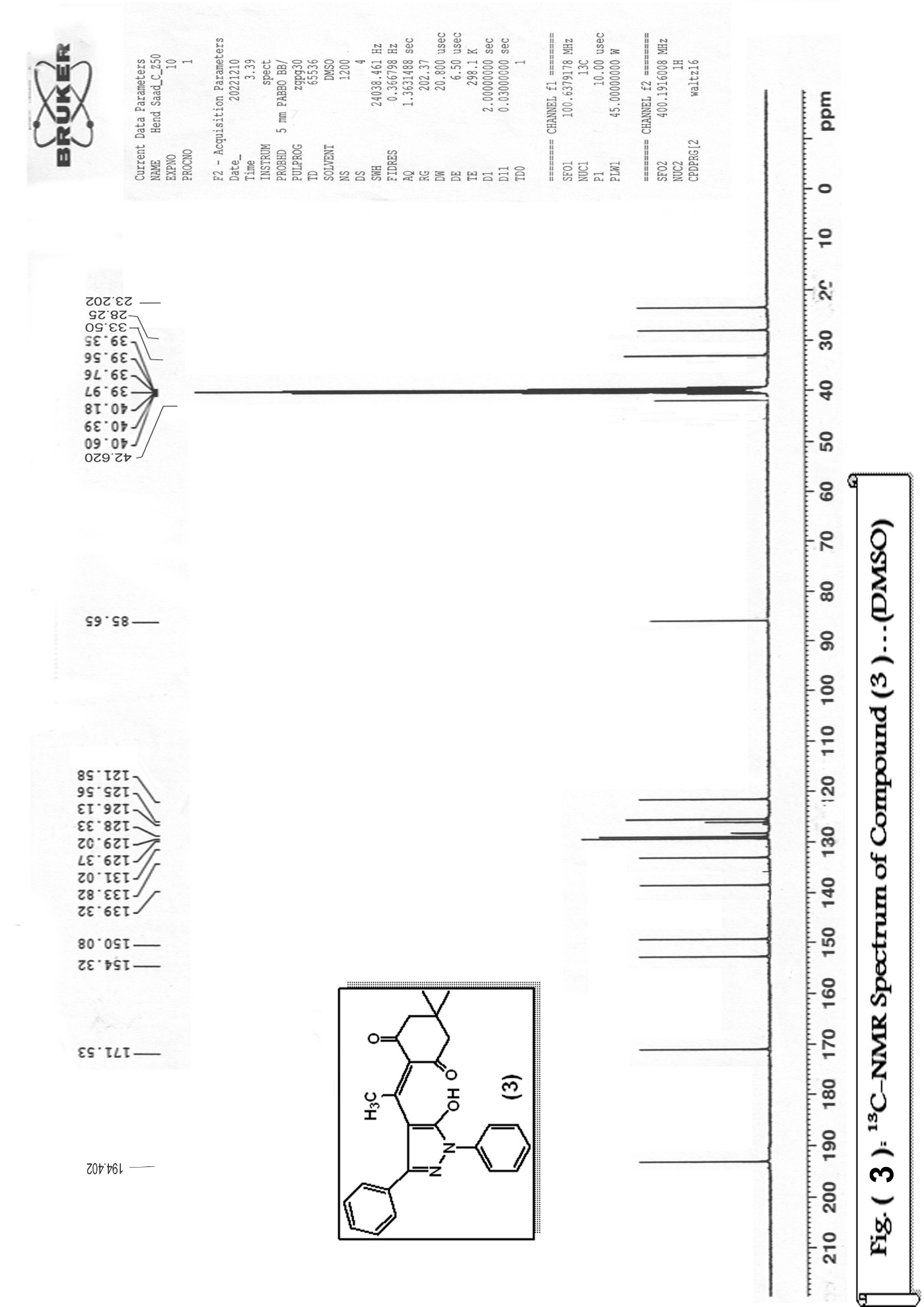


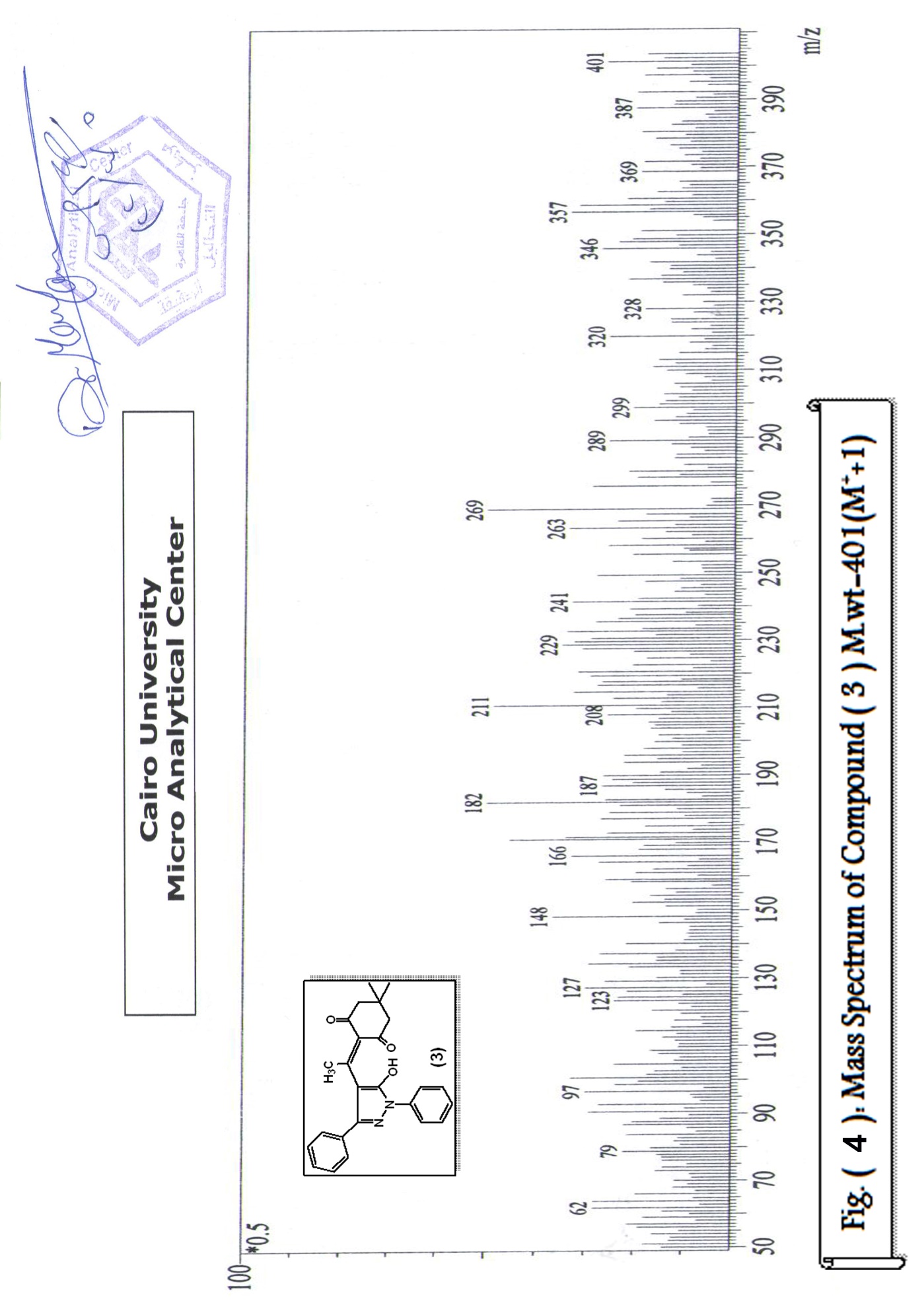


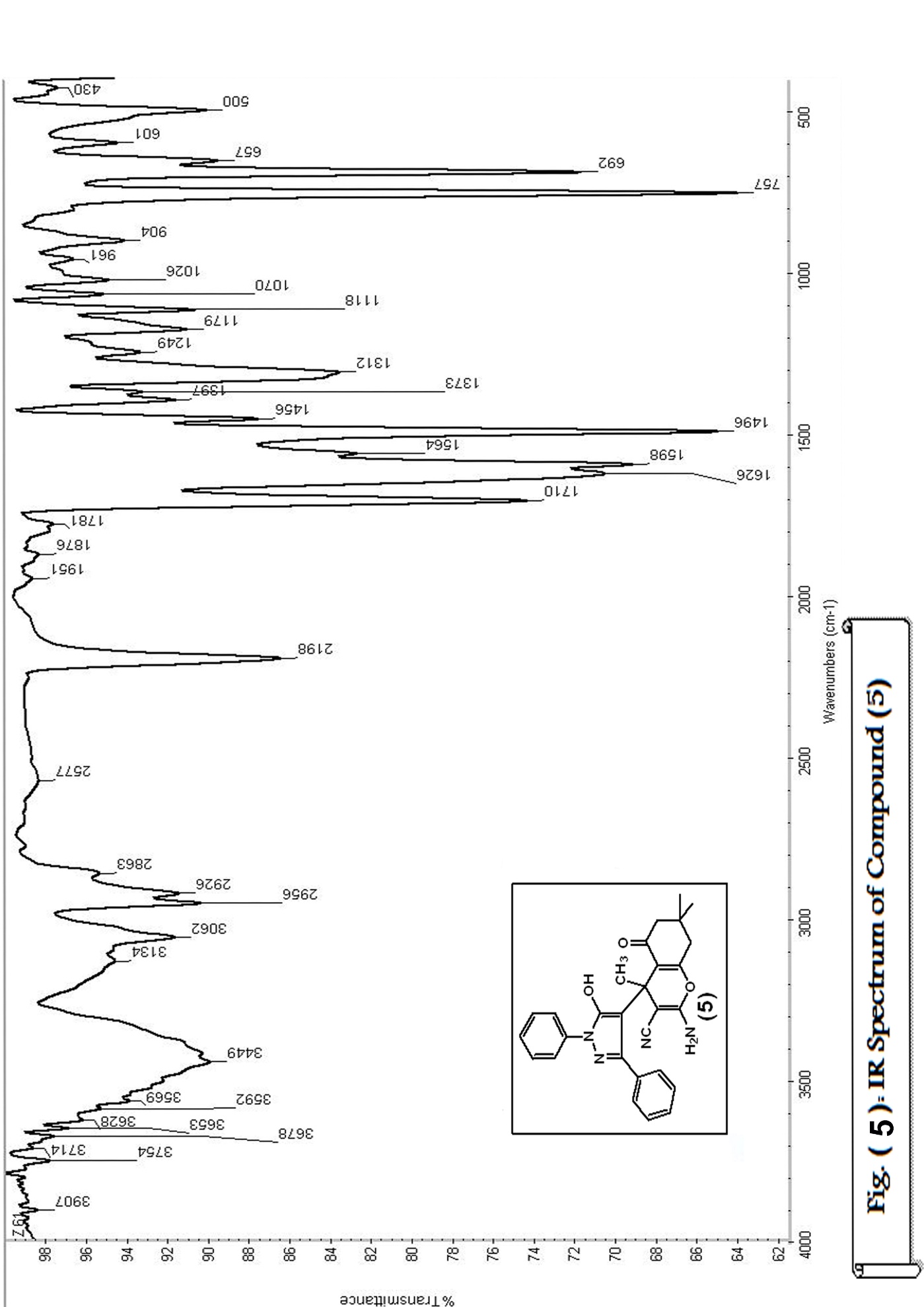


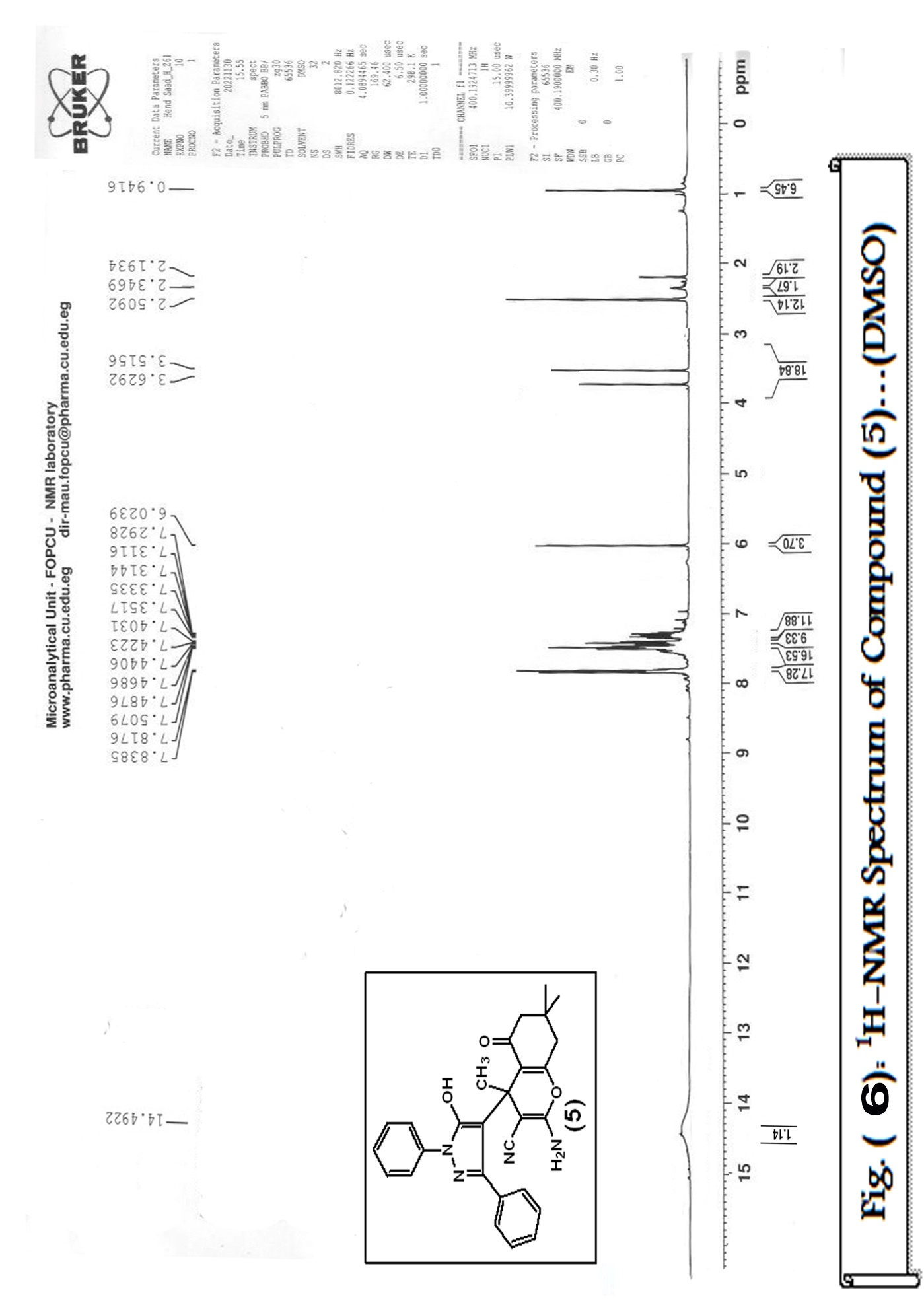


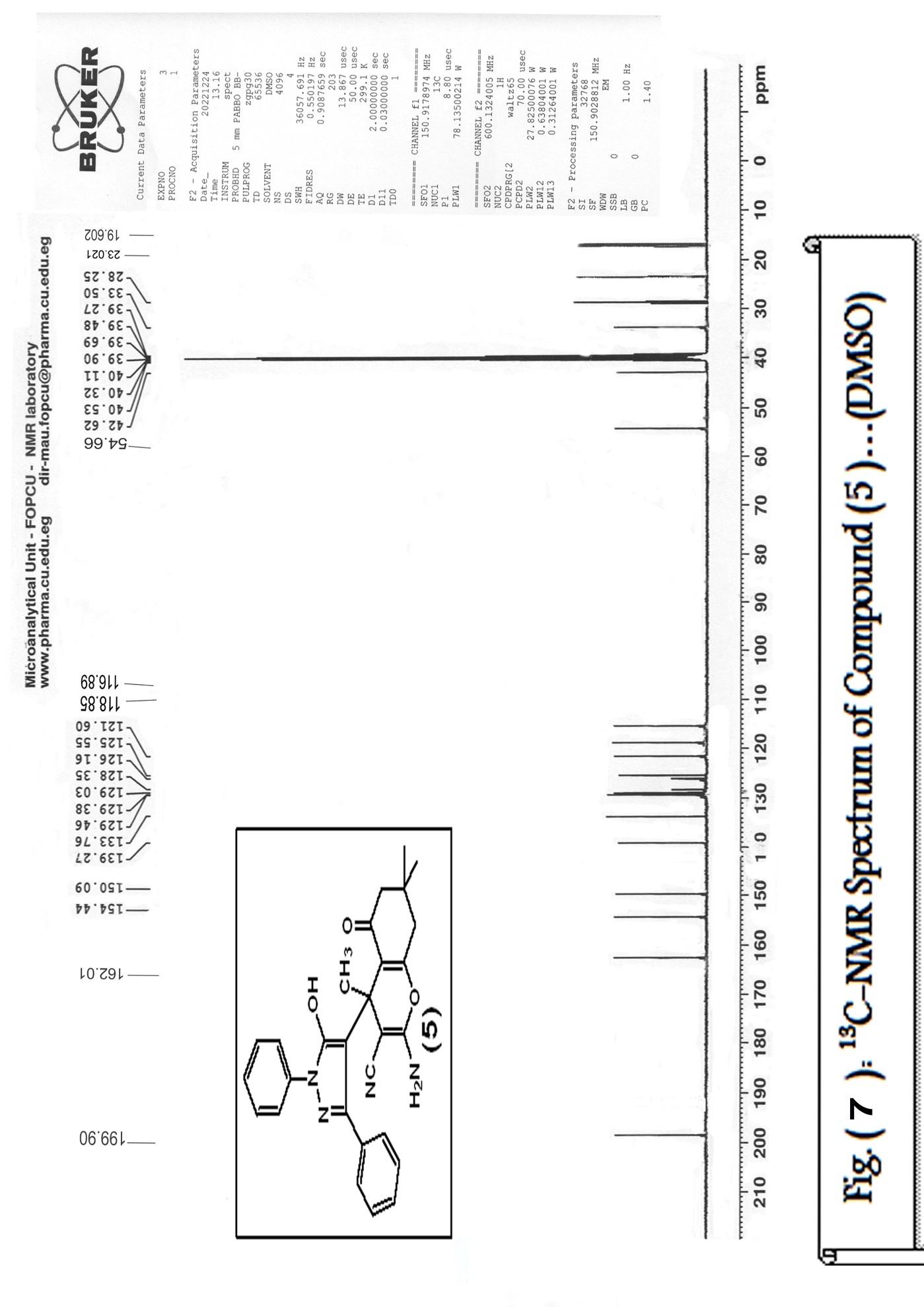


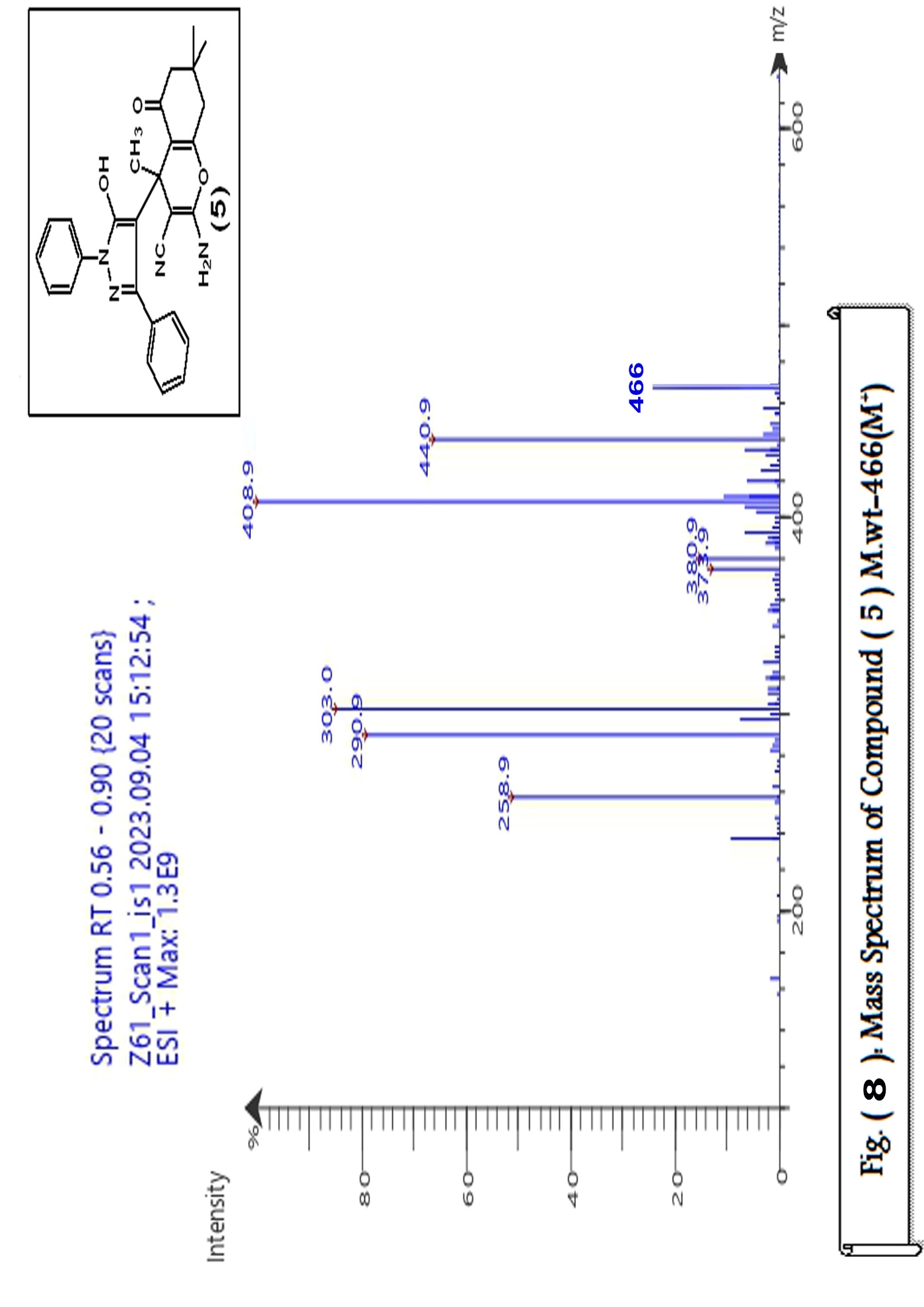


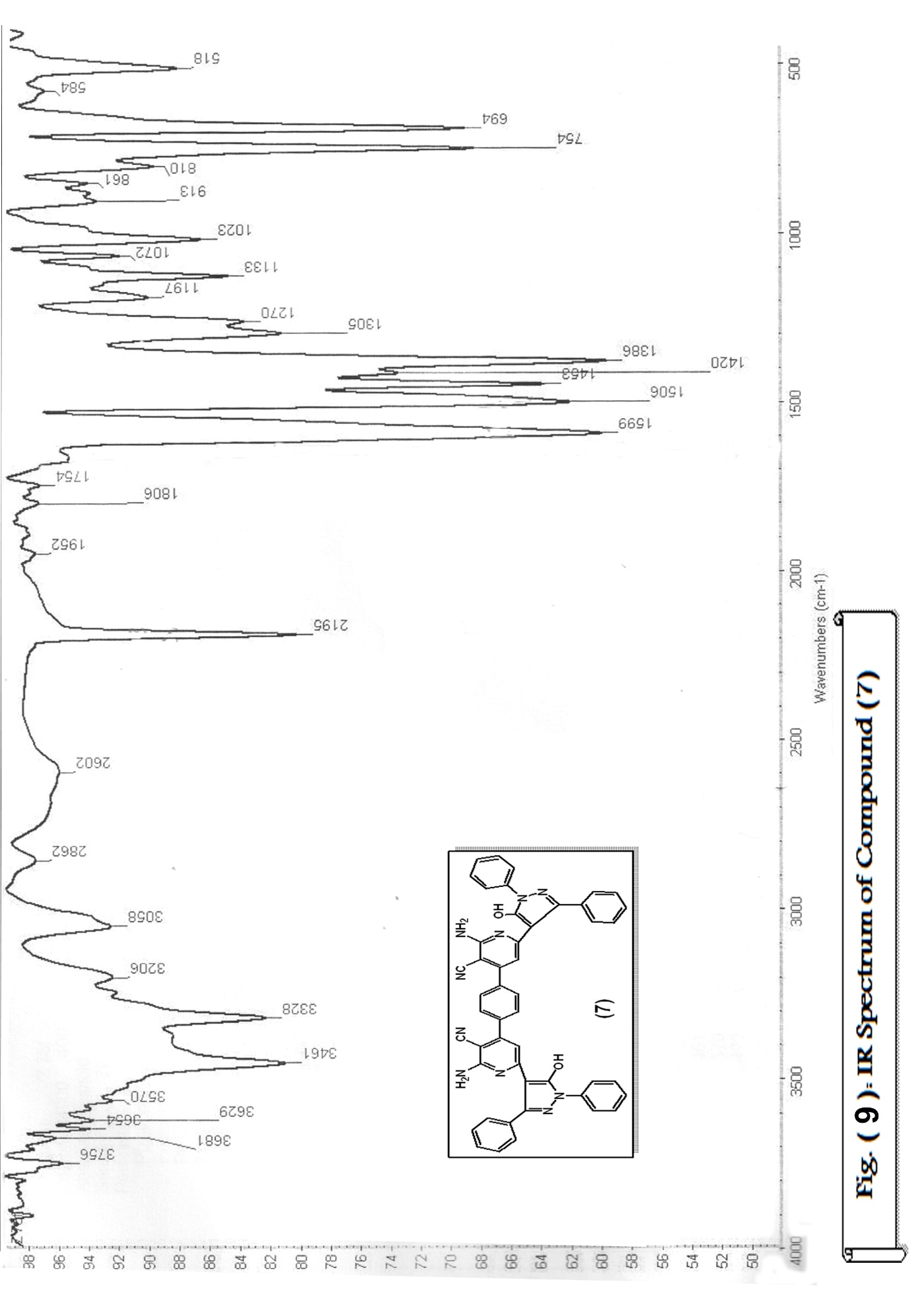


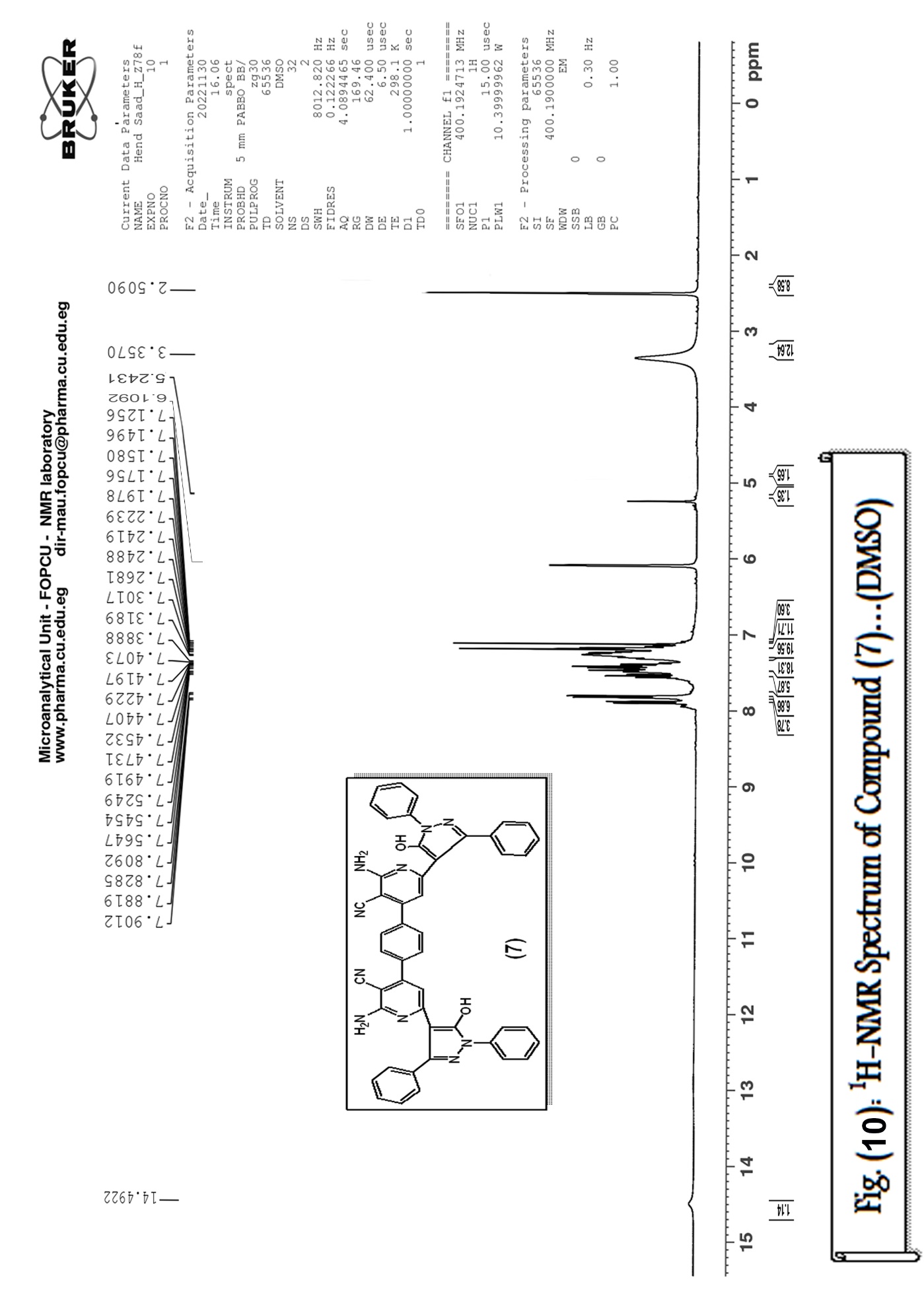


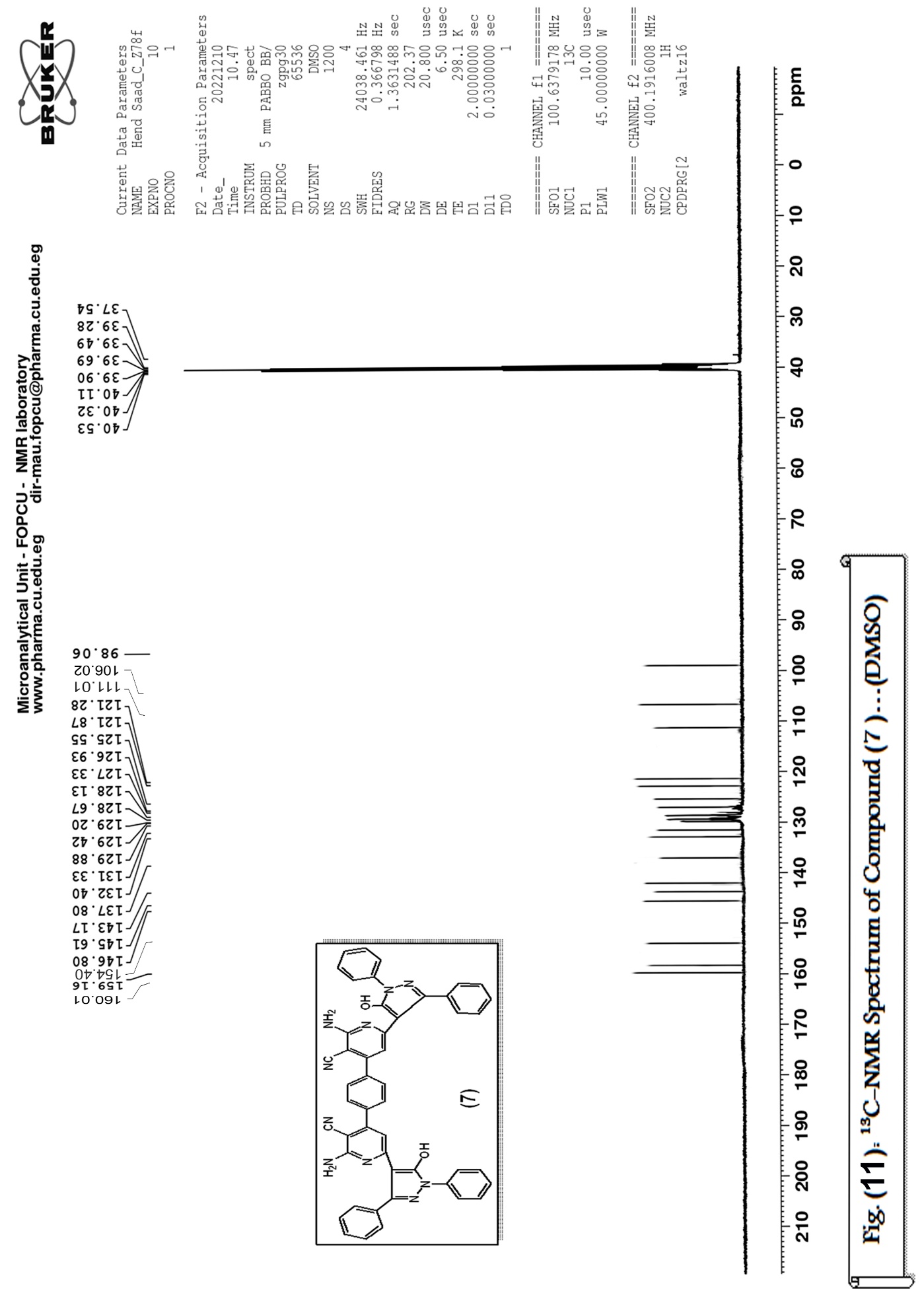


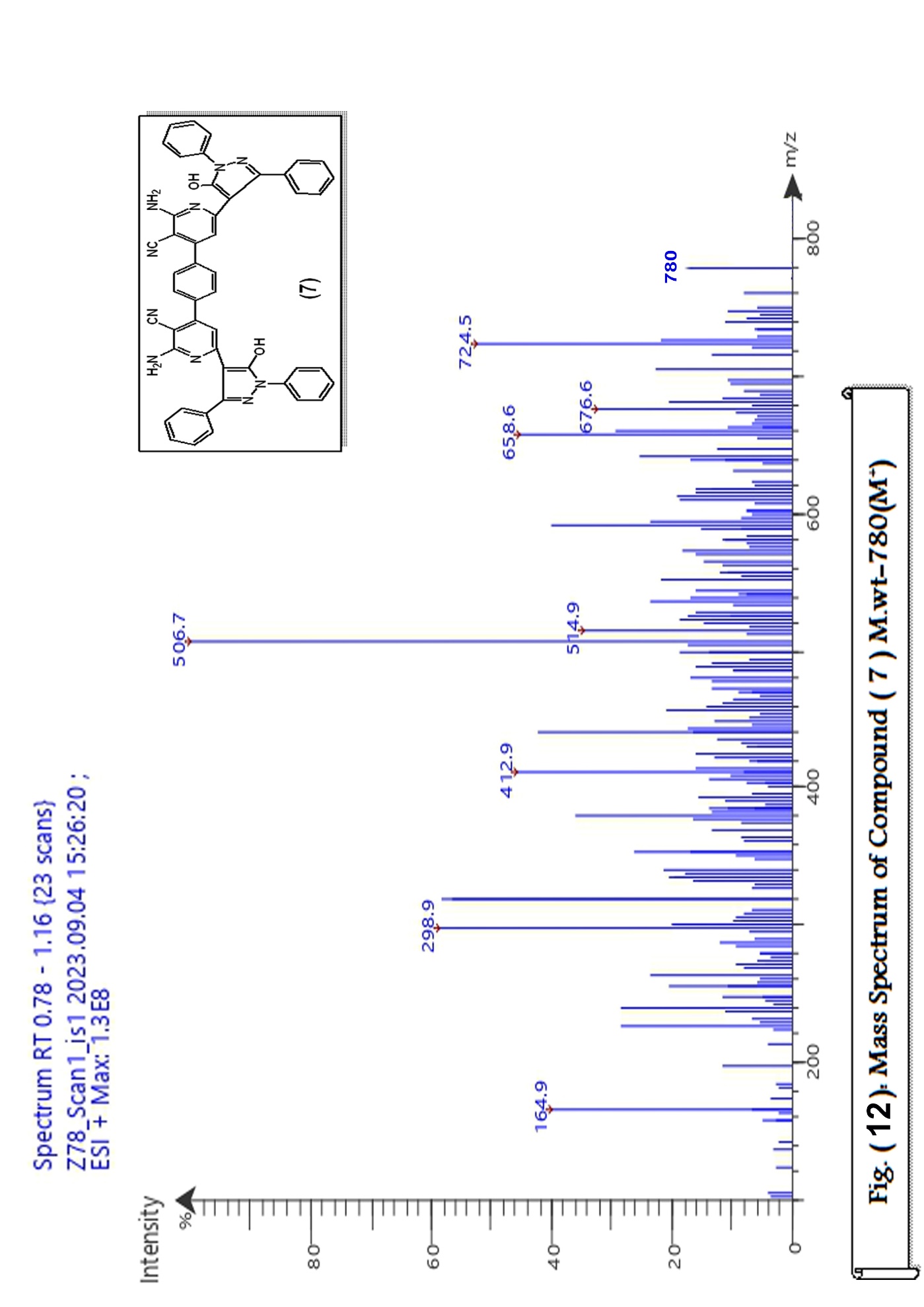


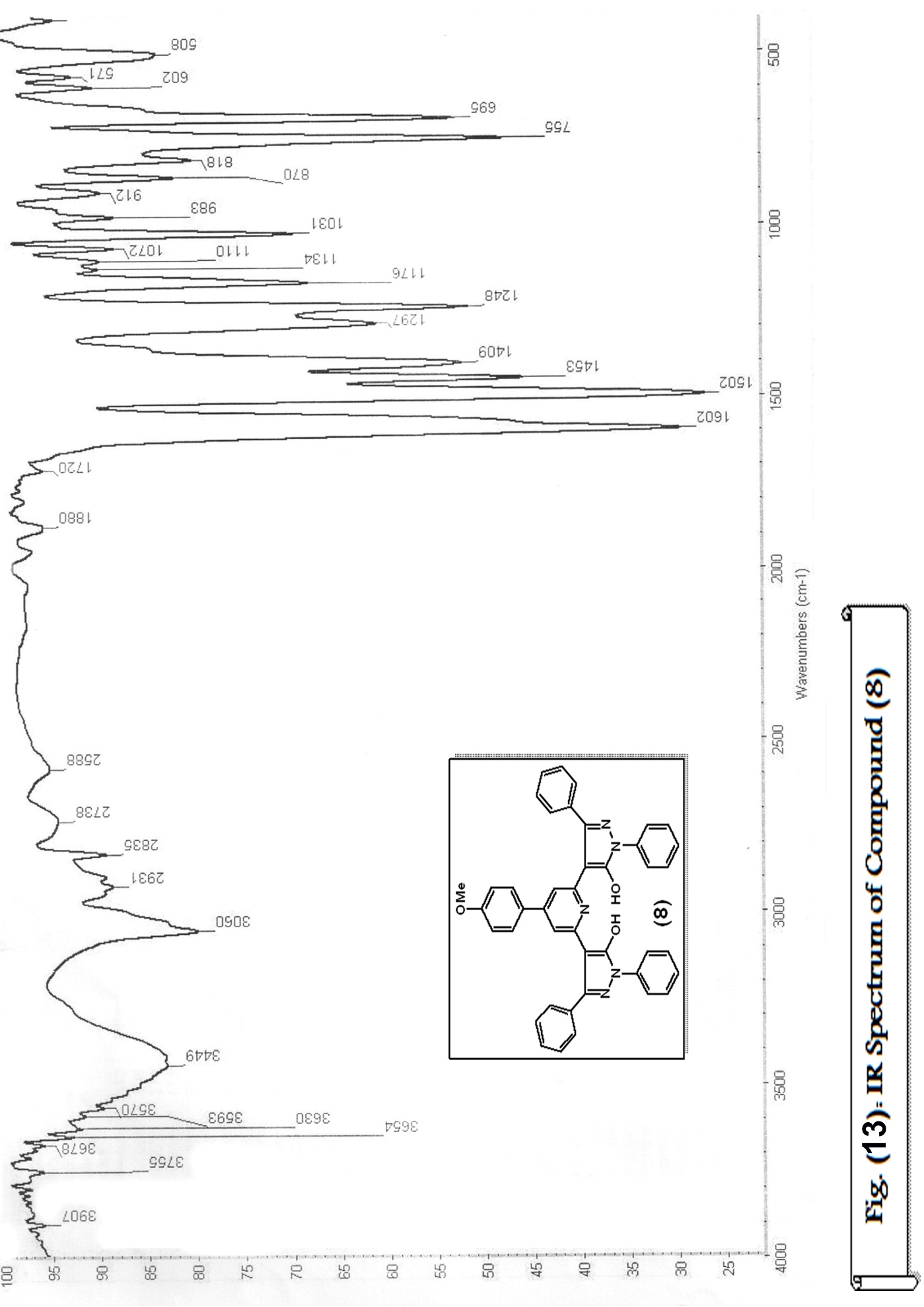


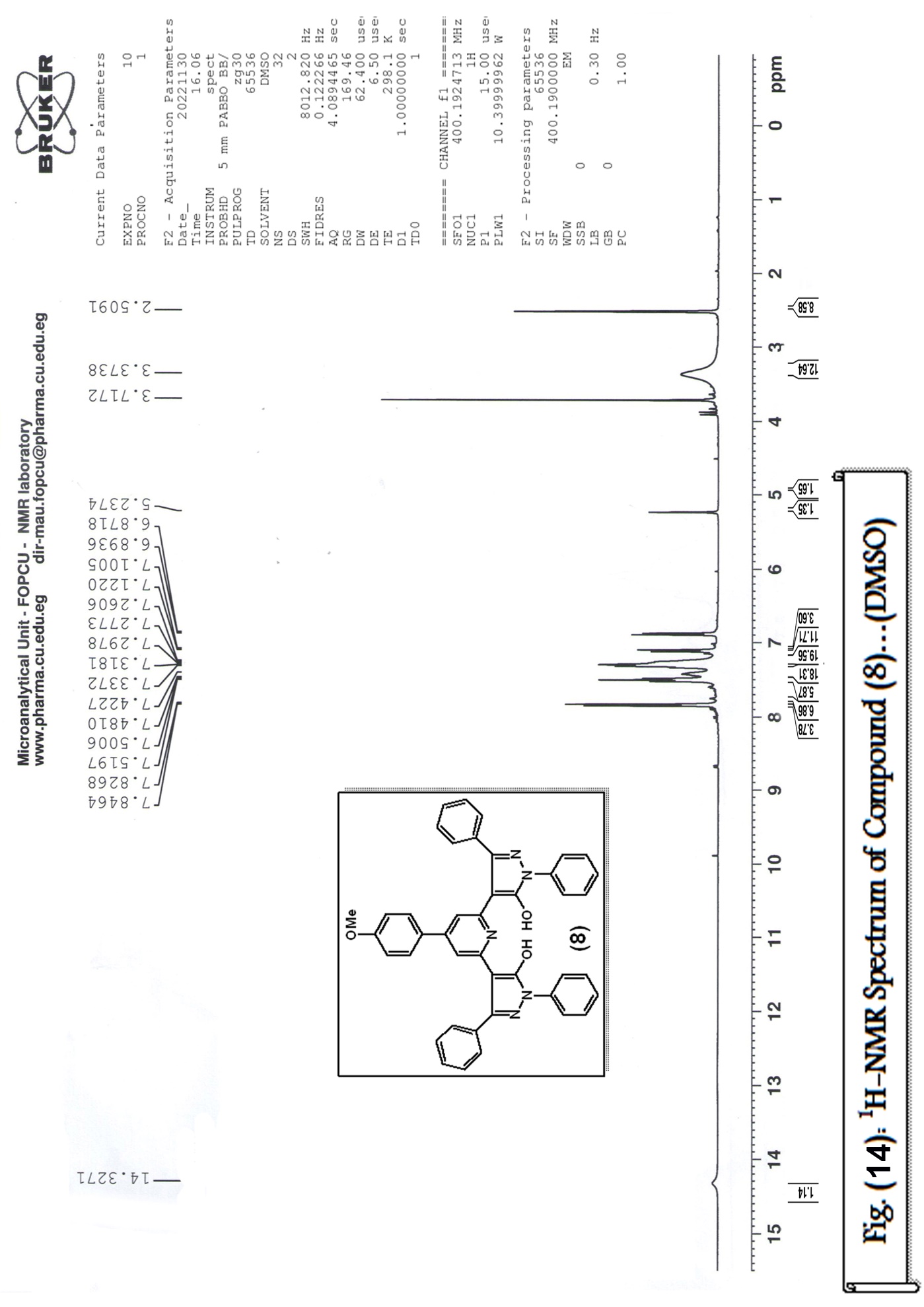


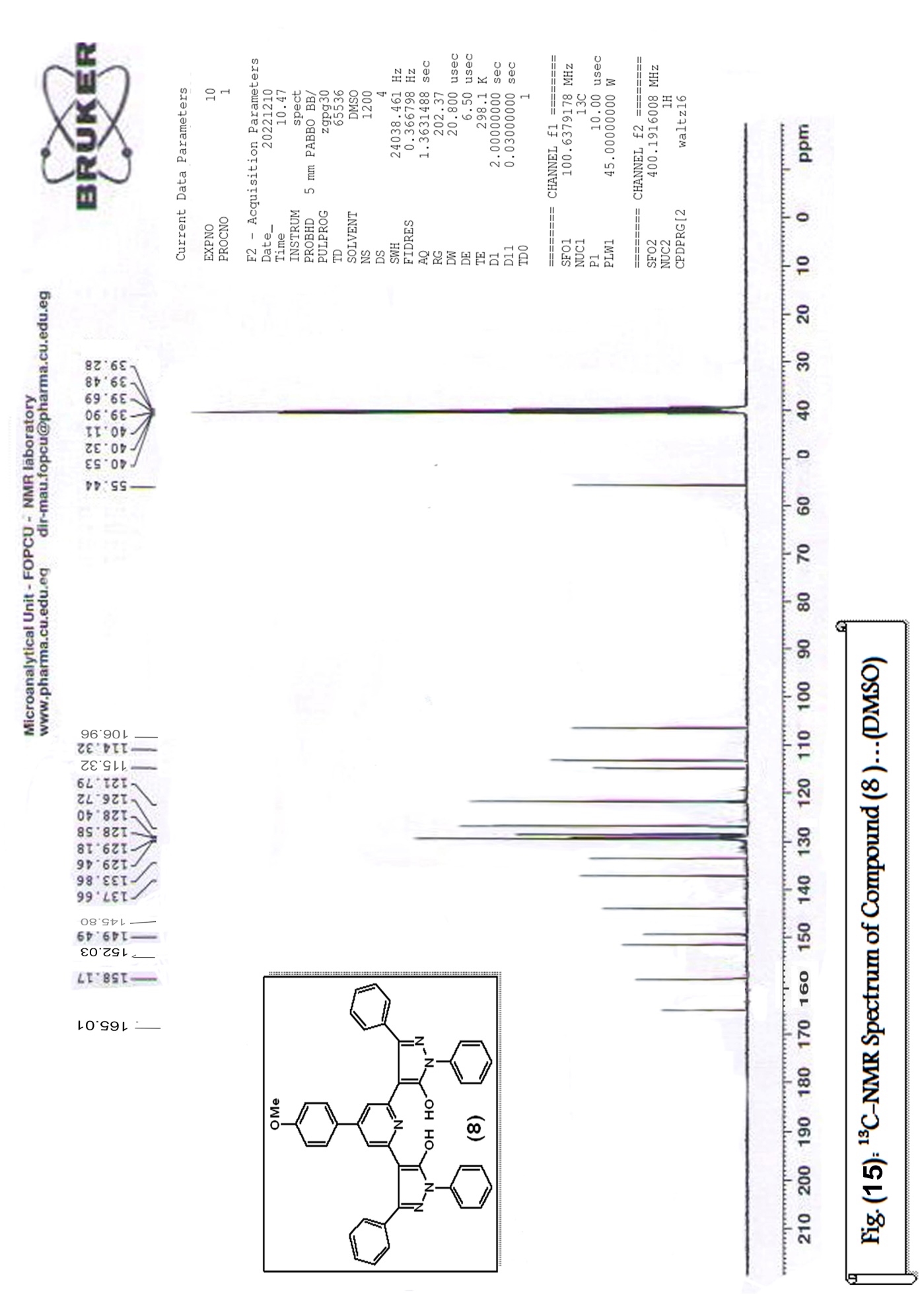


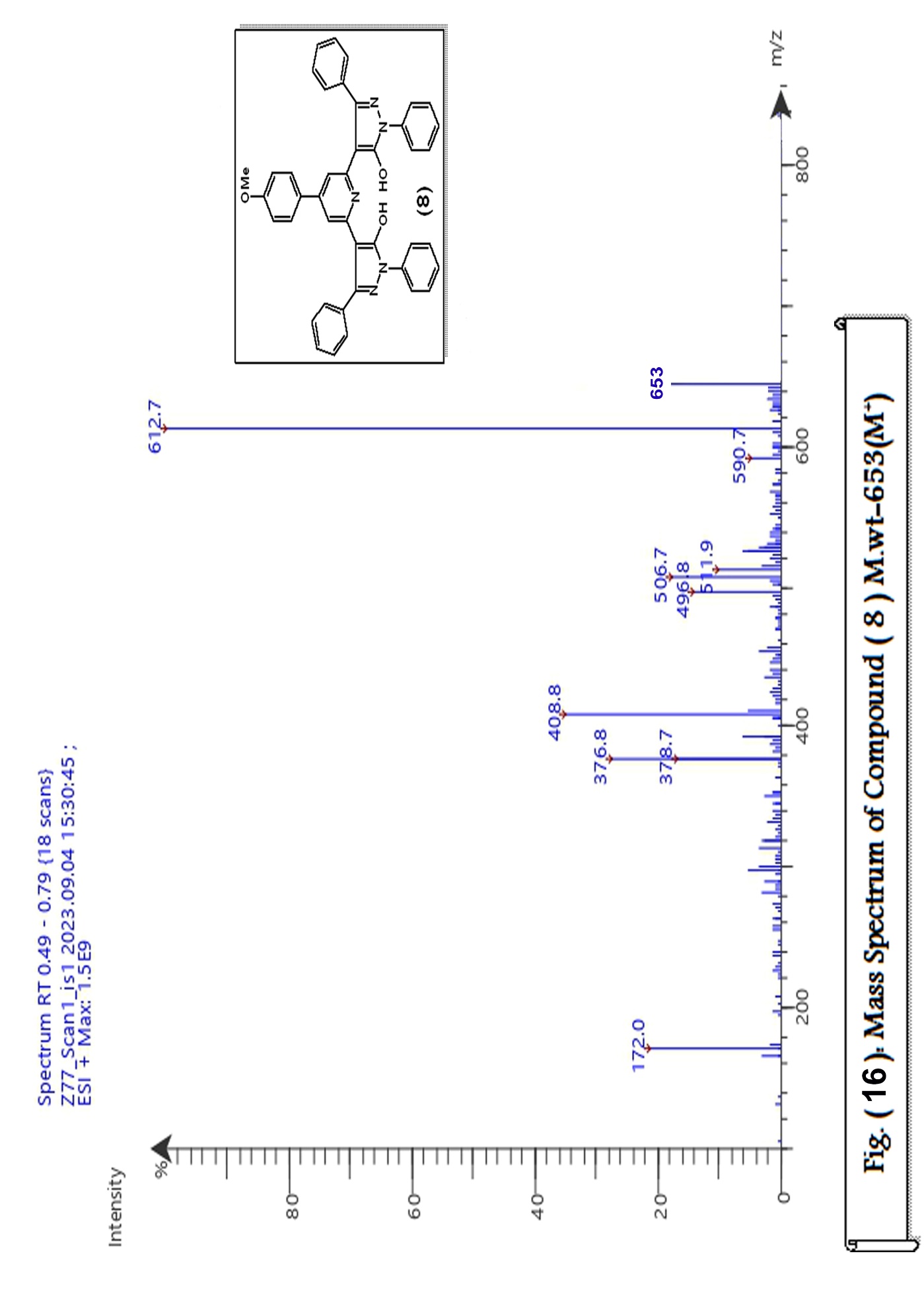


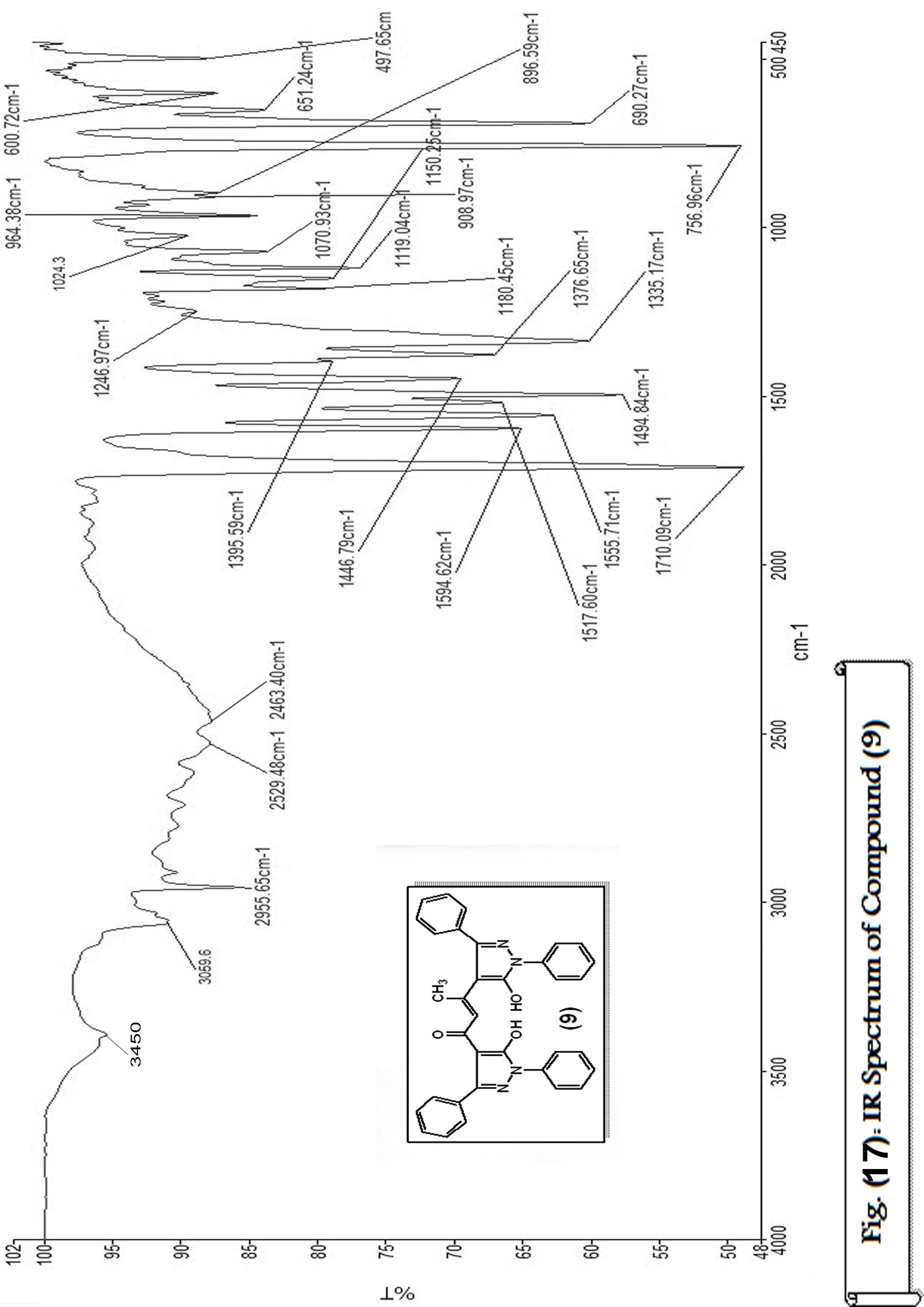


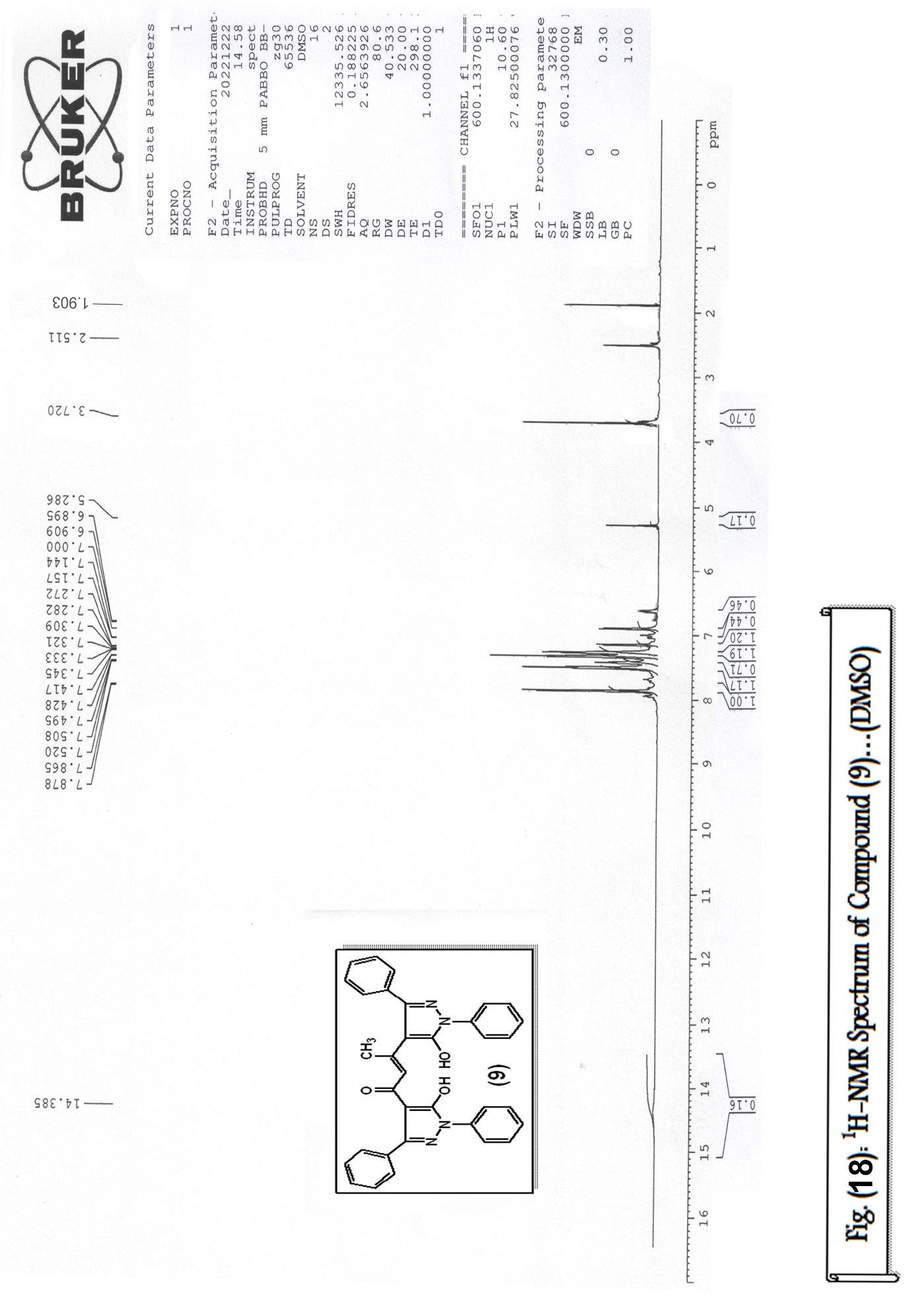


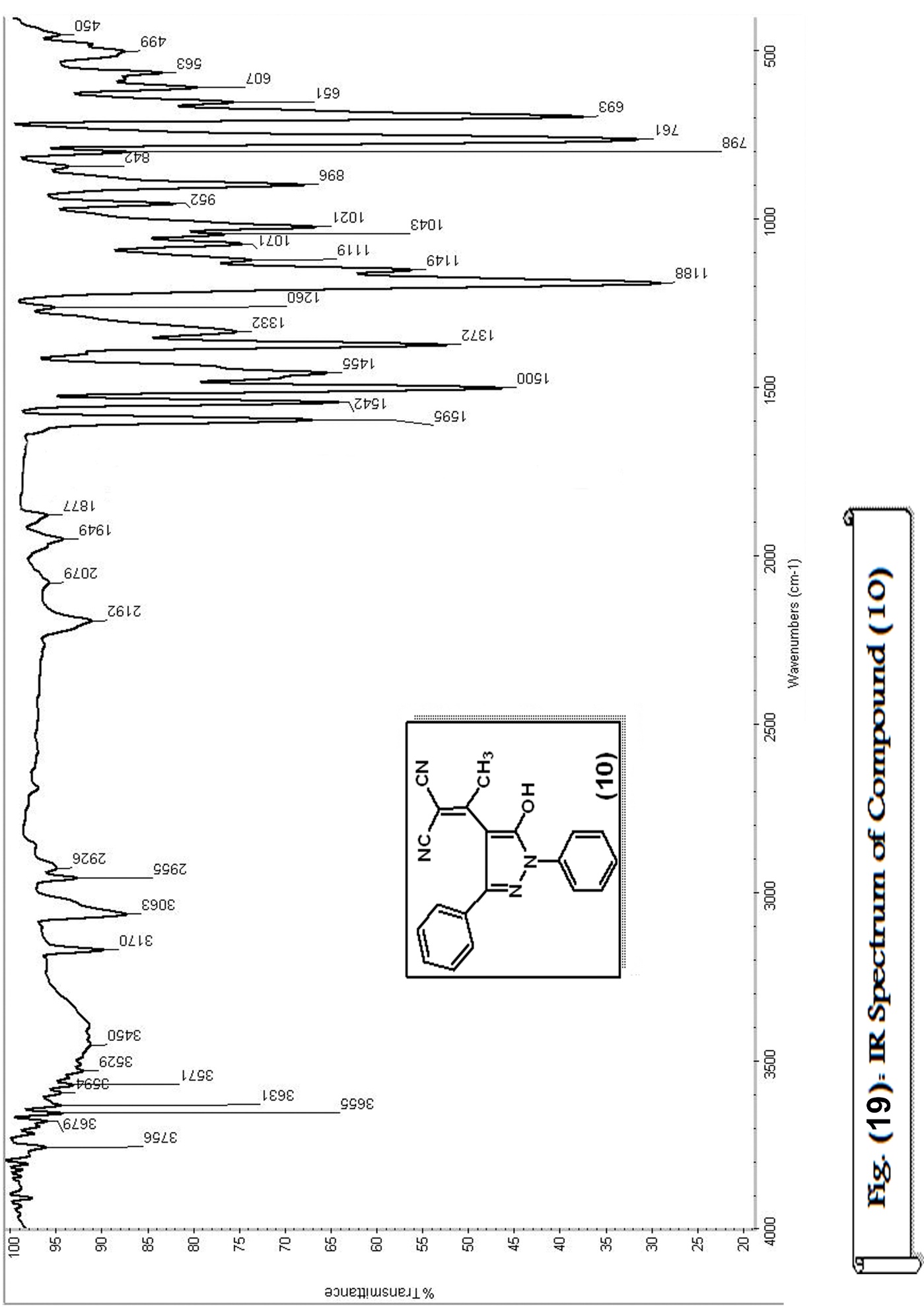


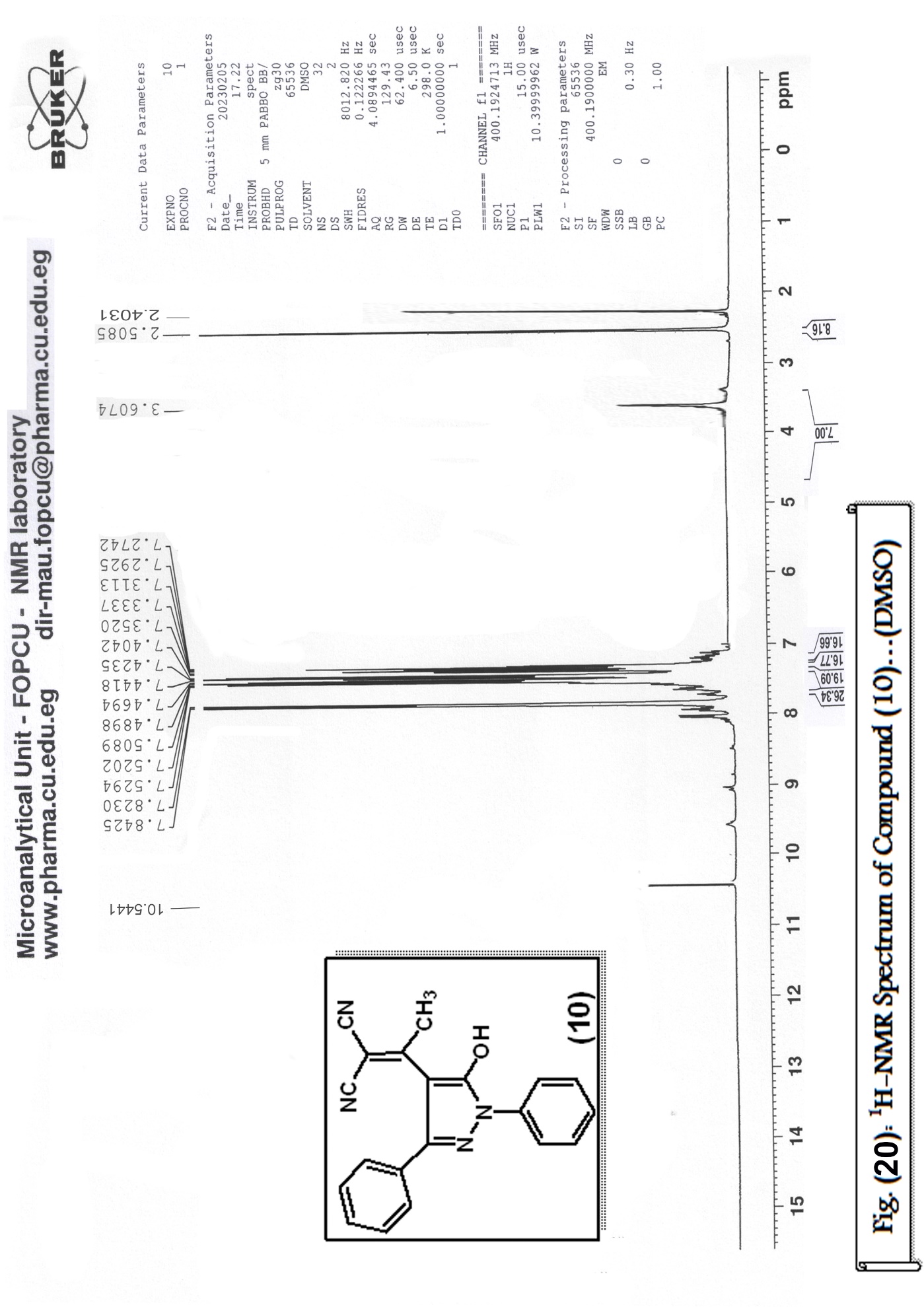


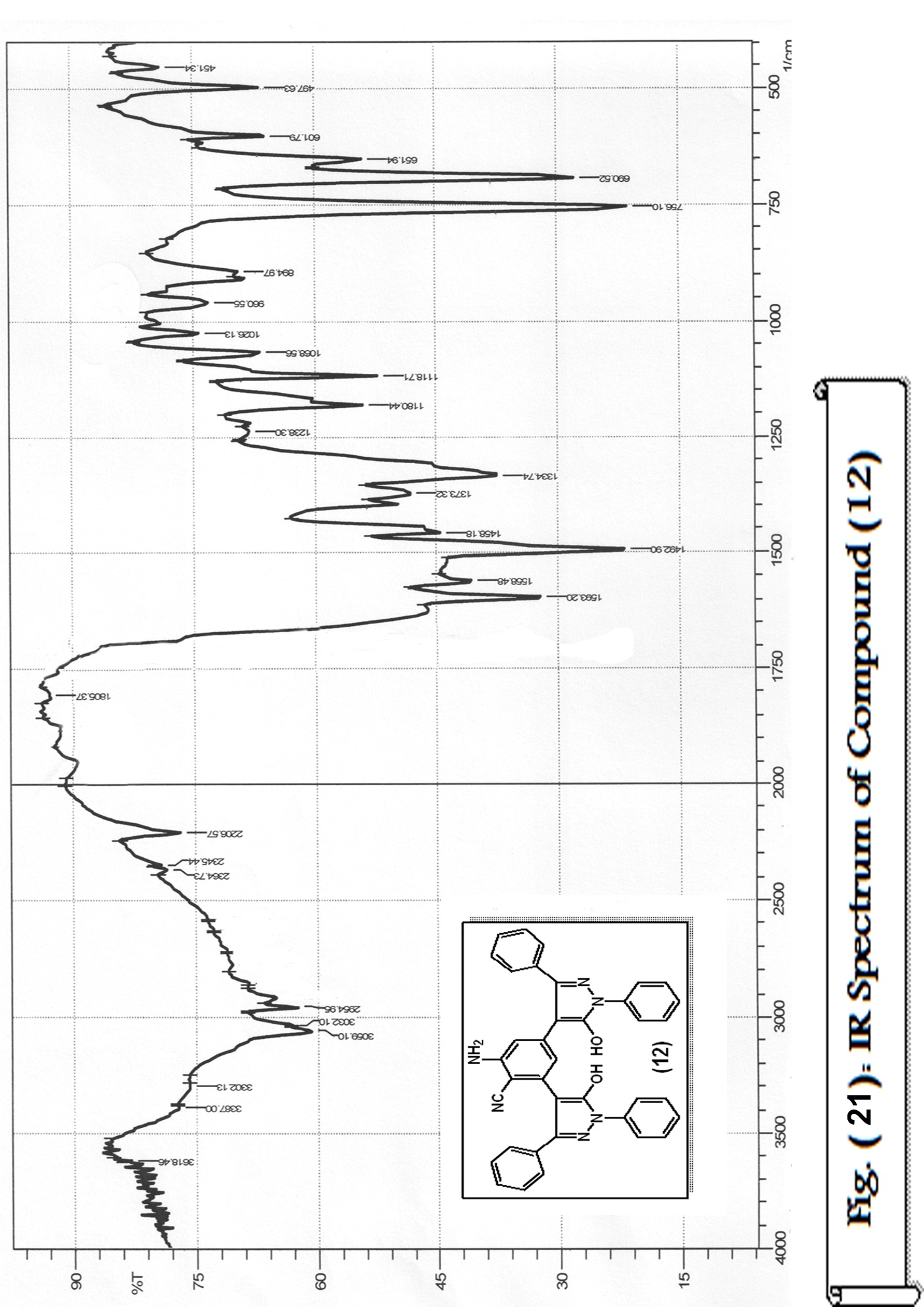


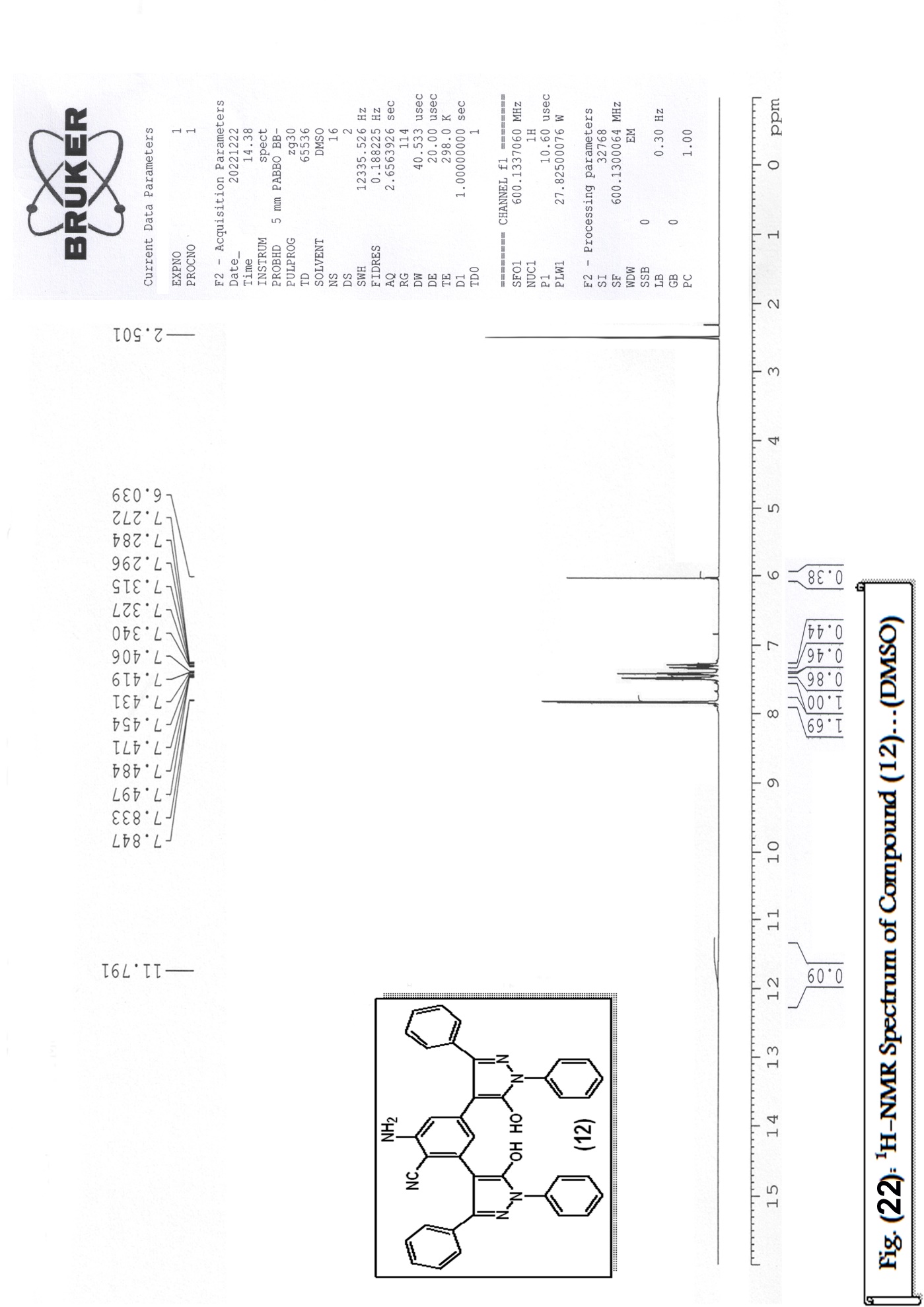


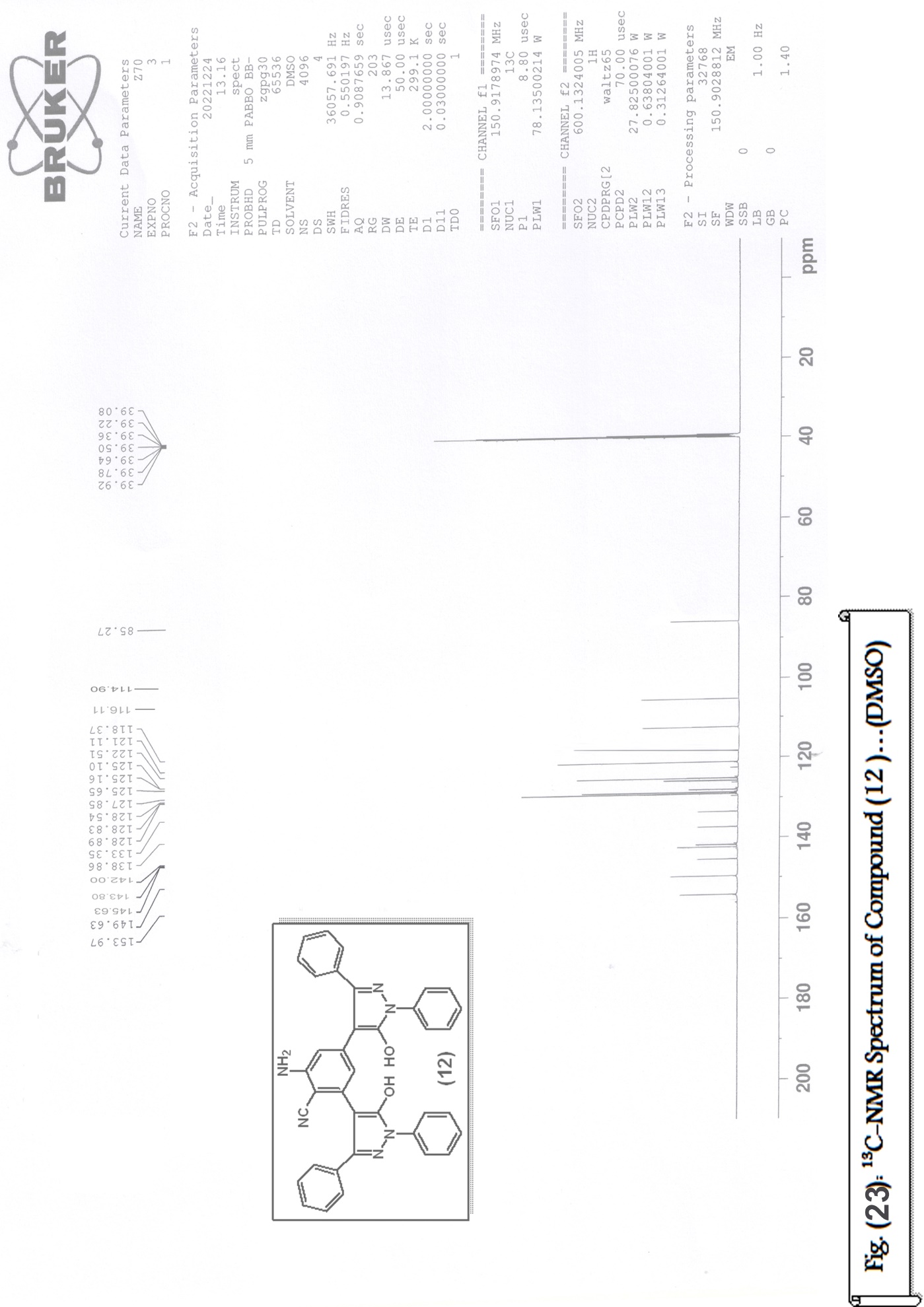


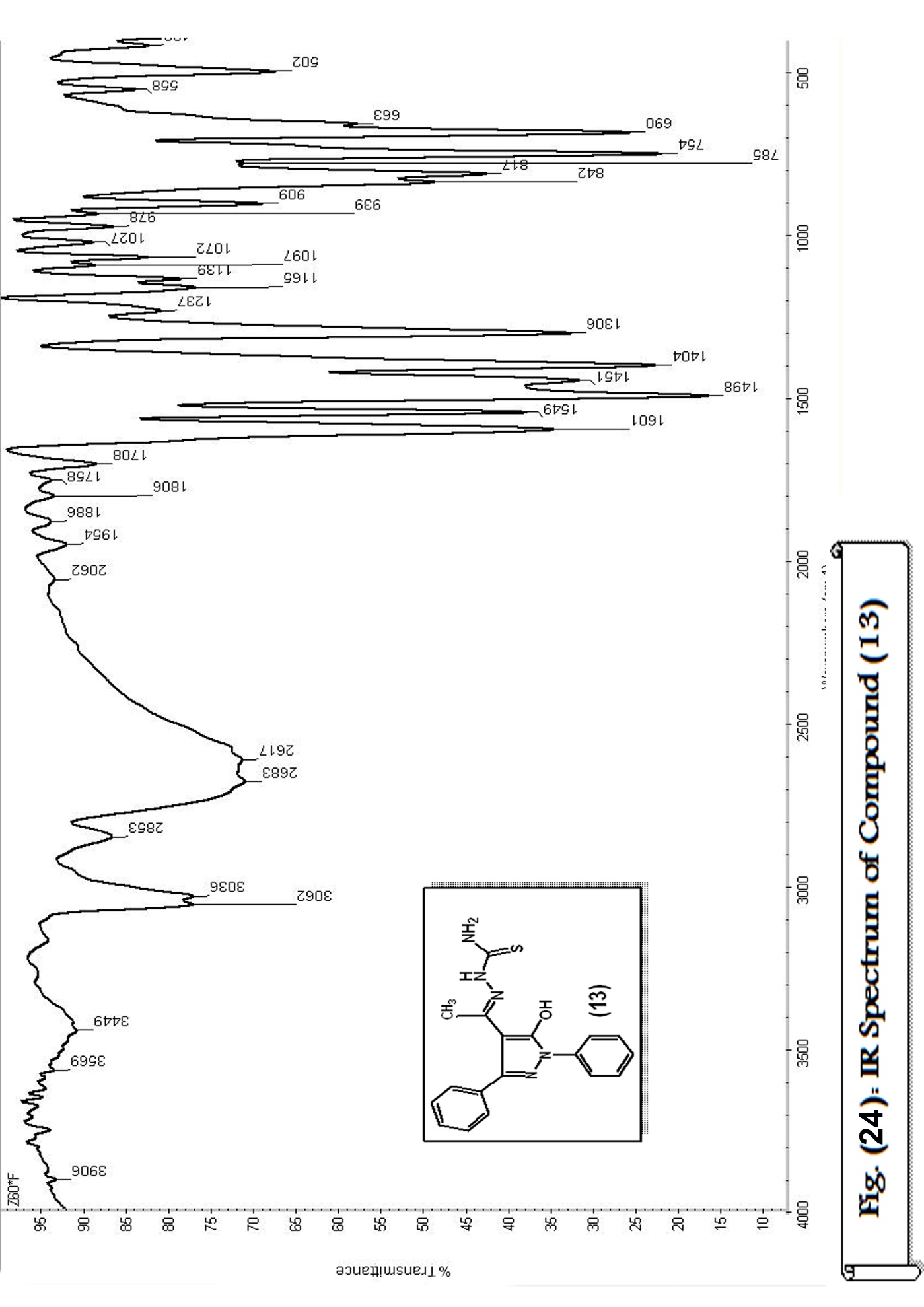


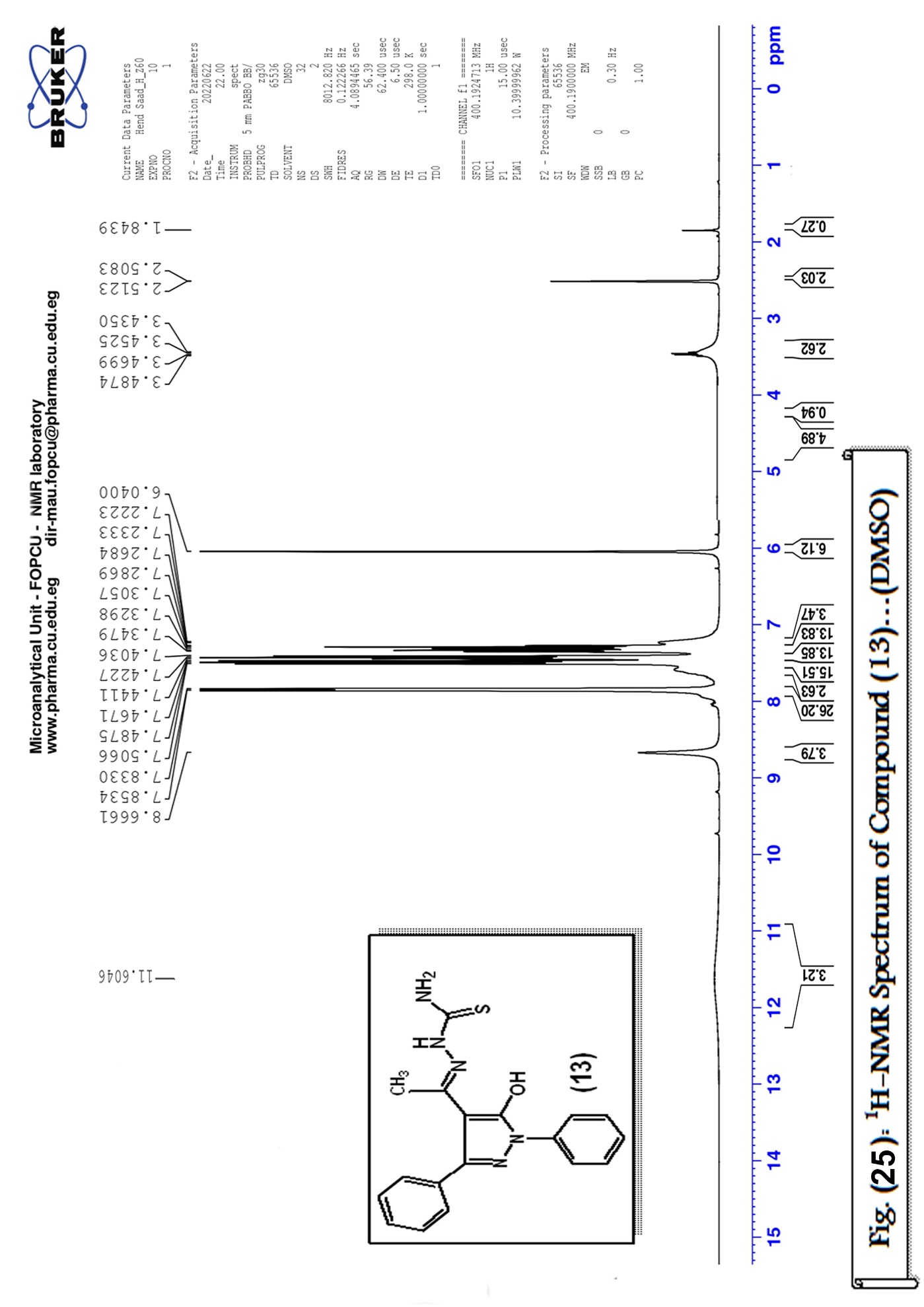


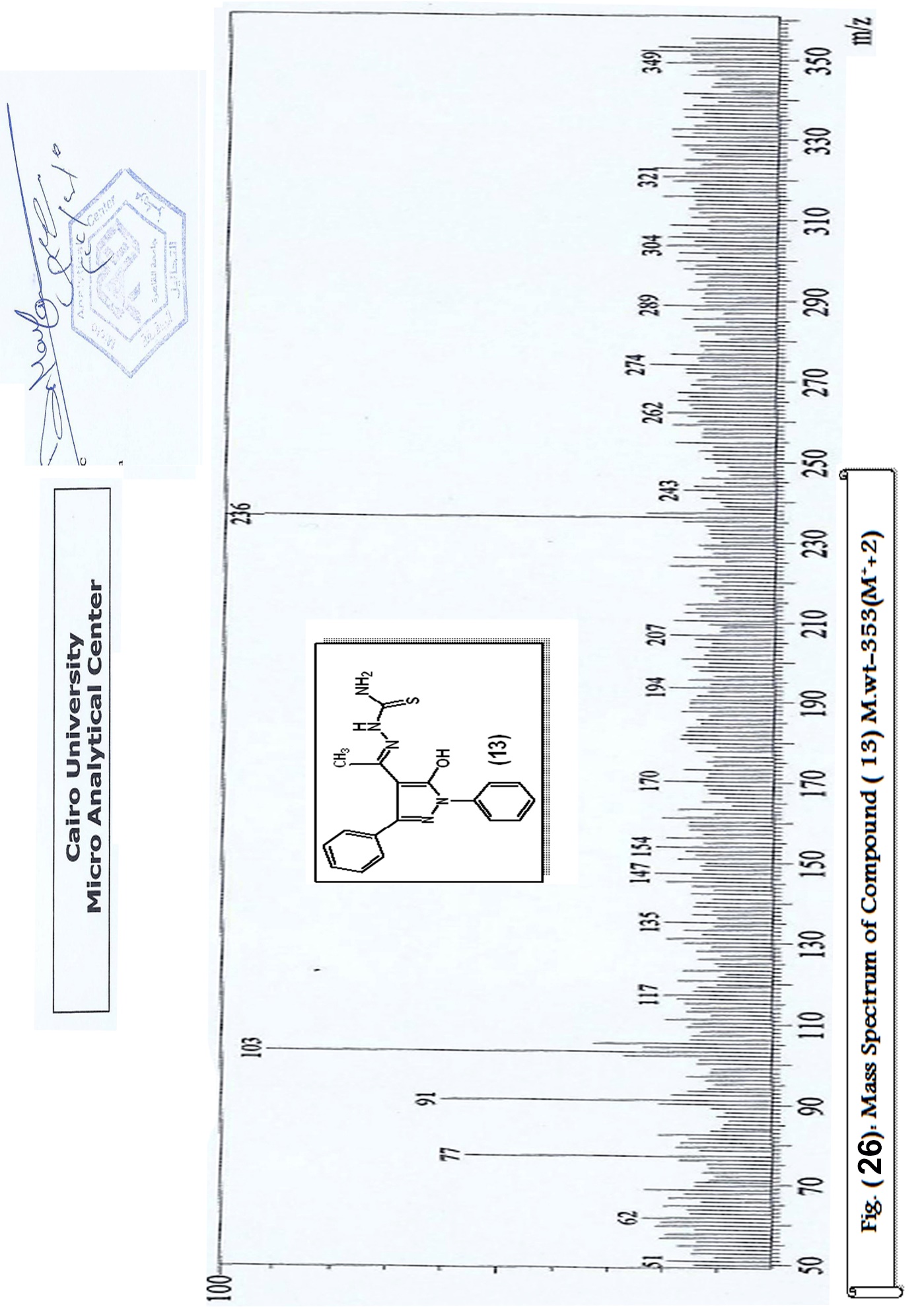


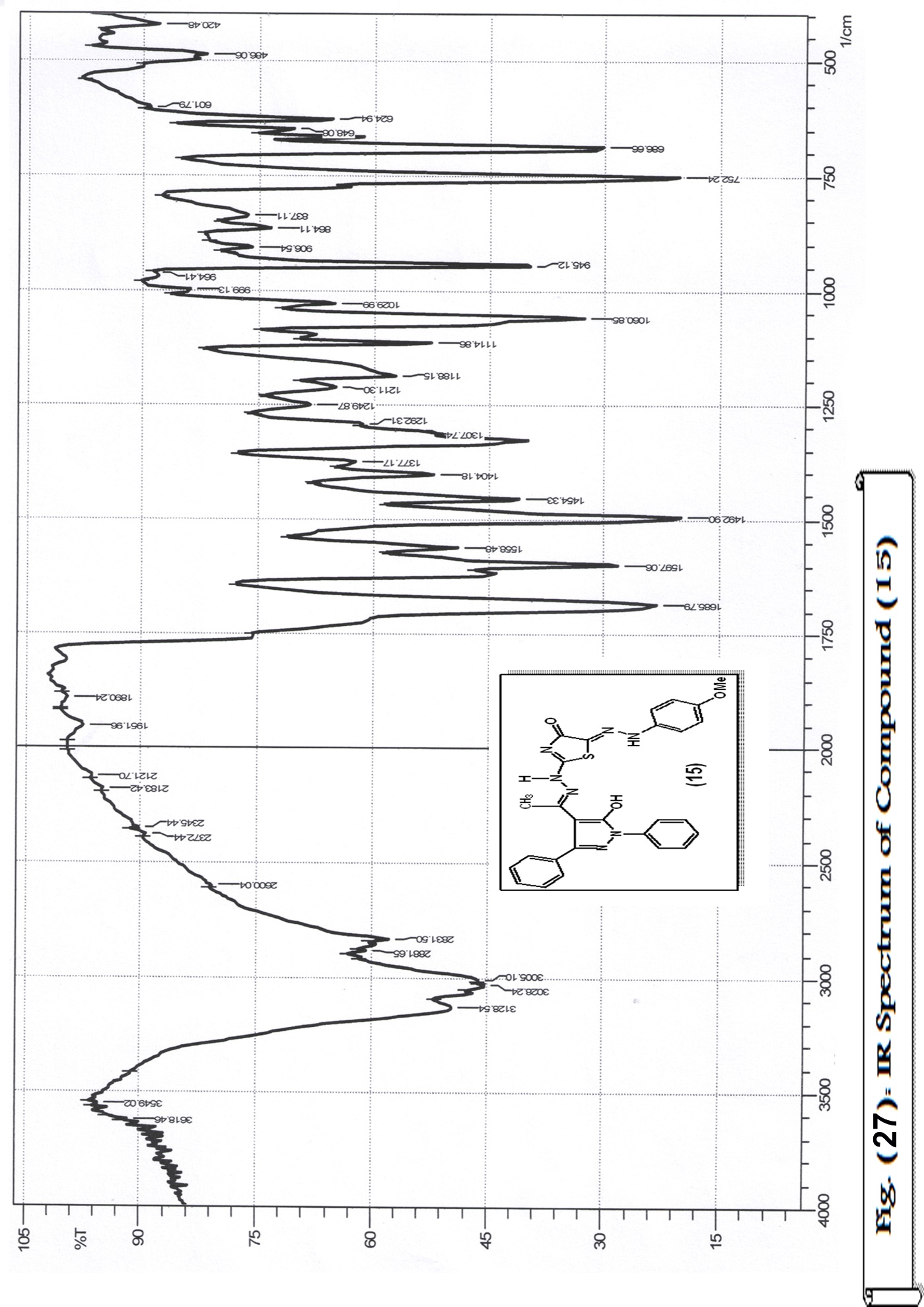


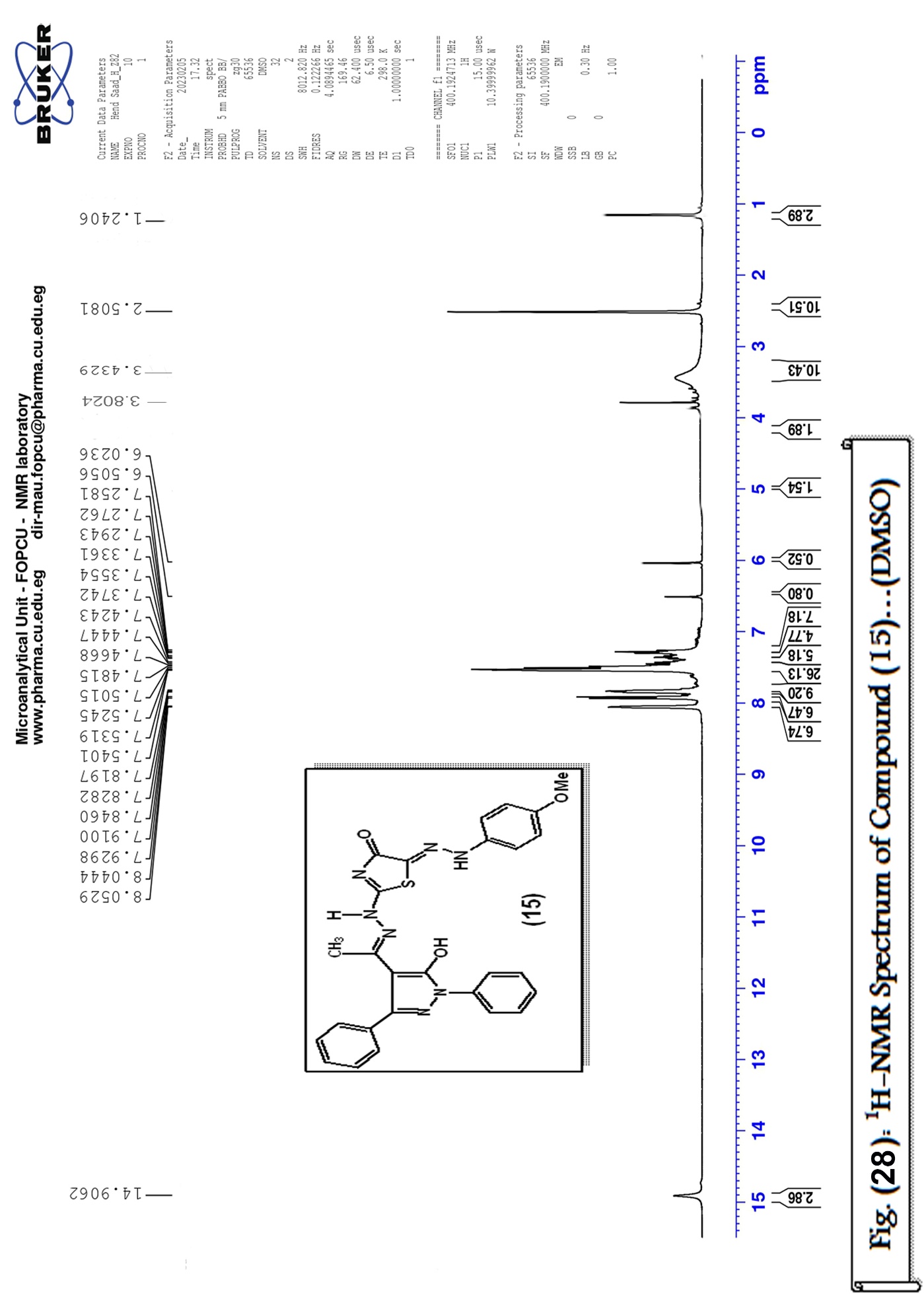


a
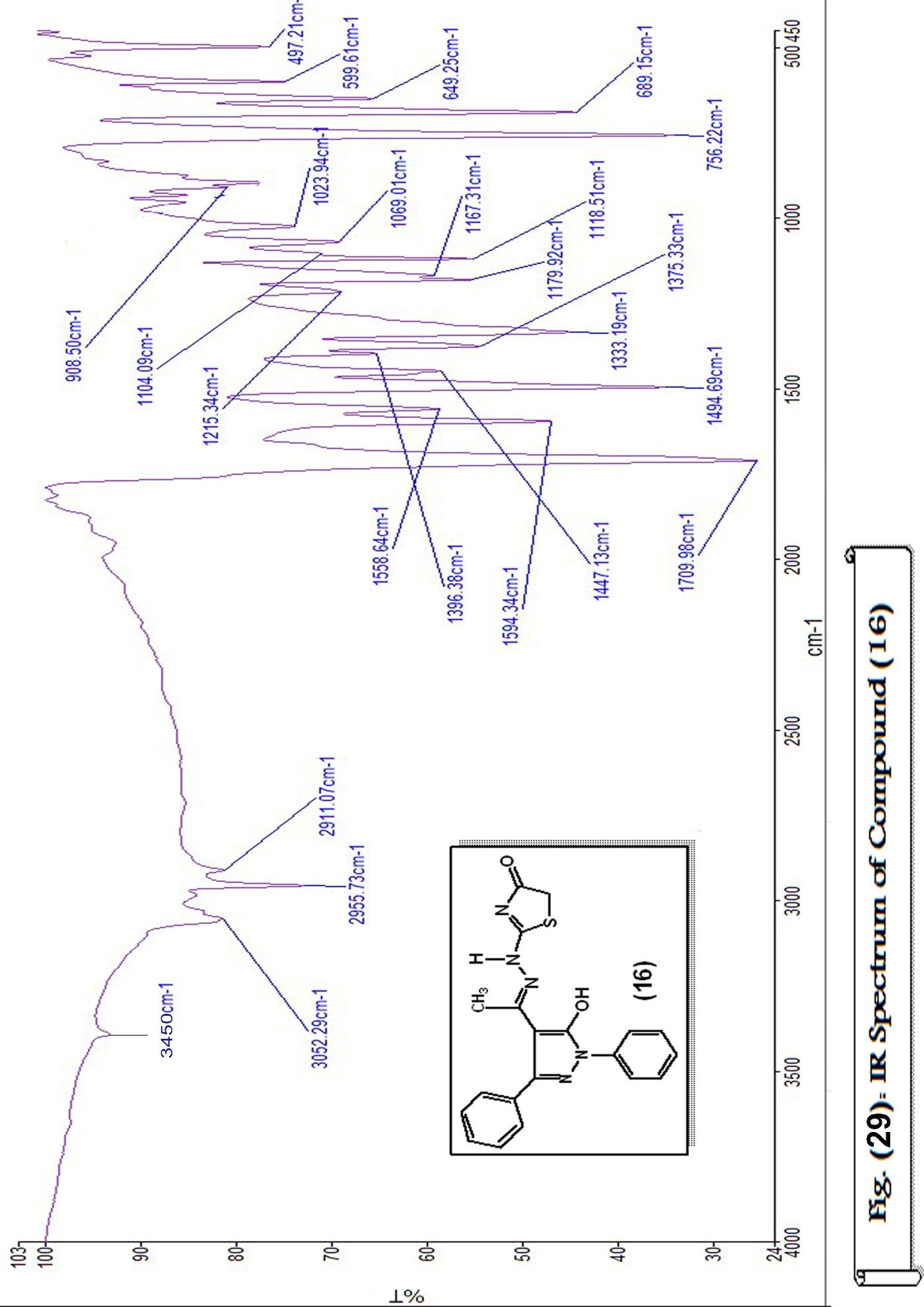


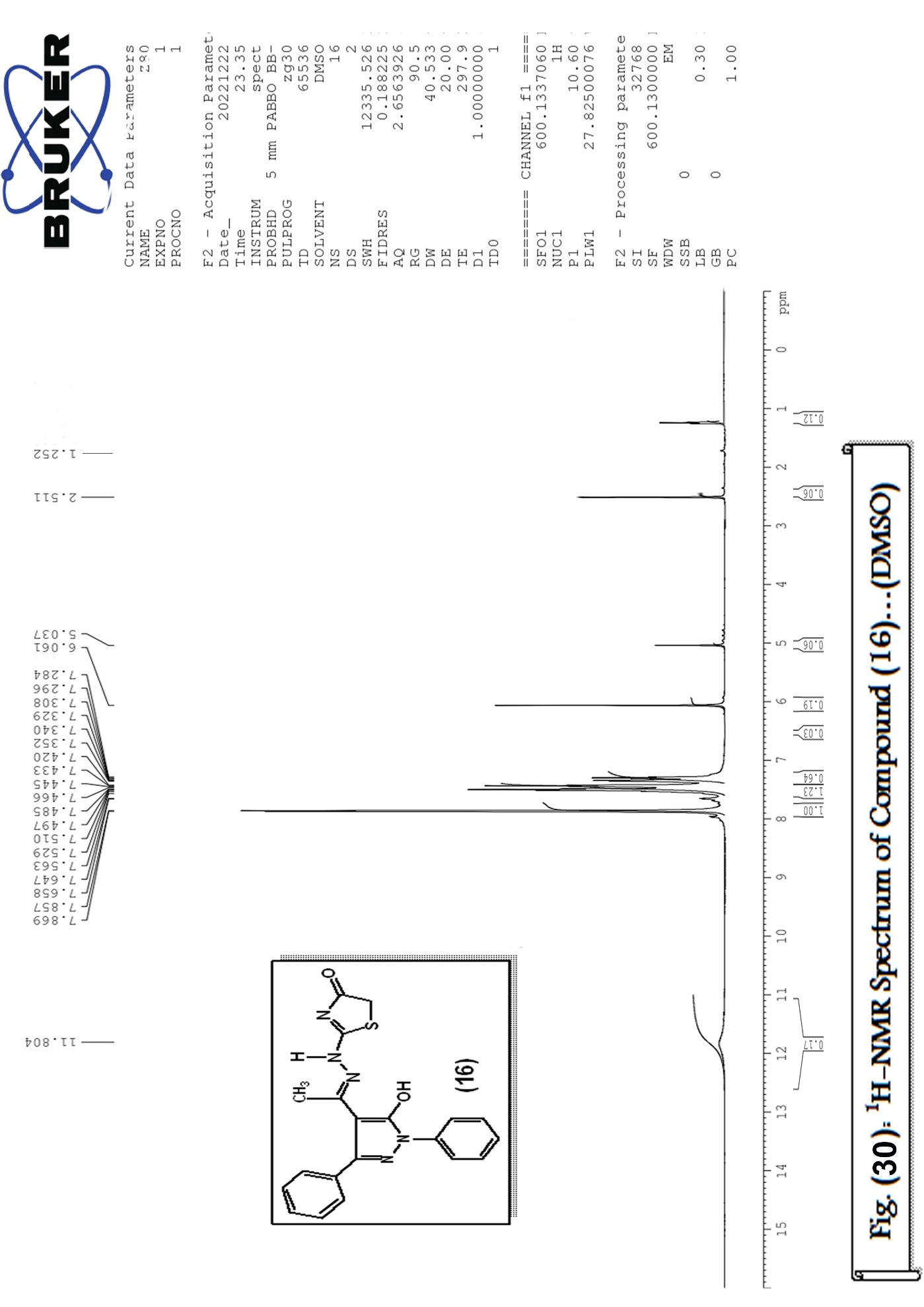


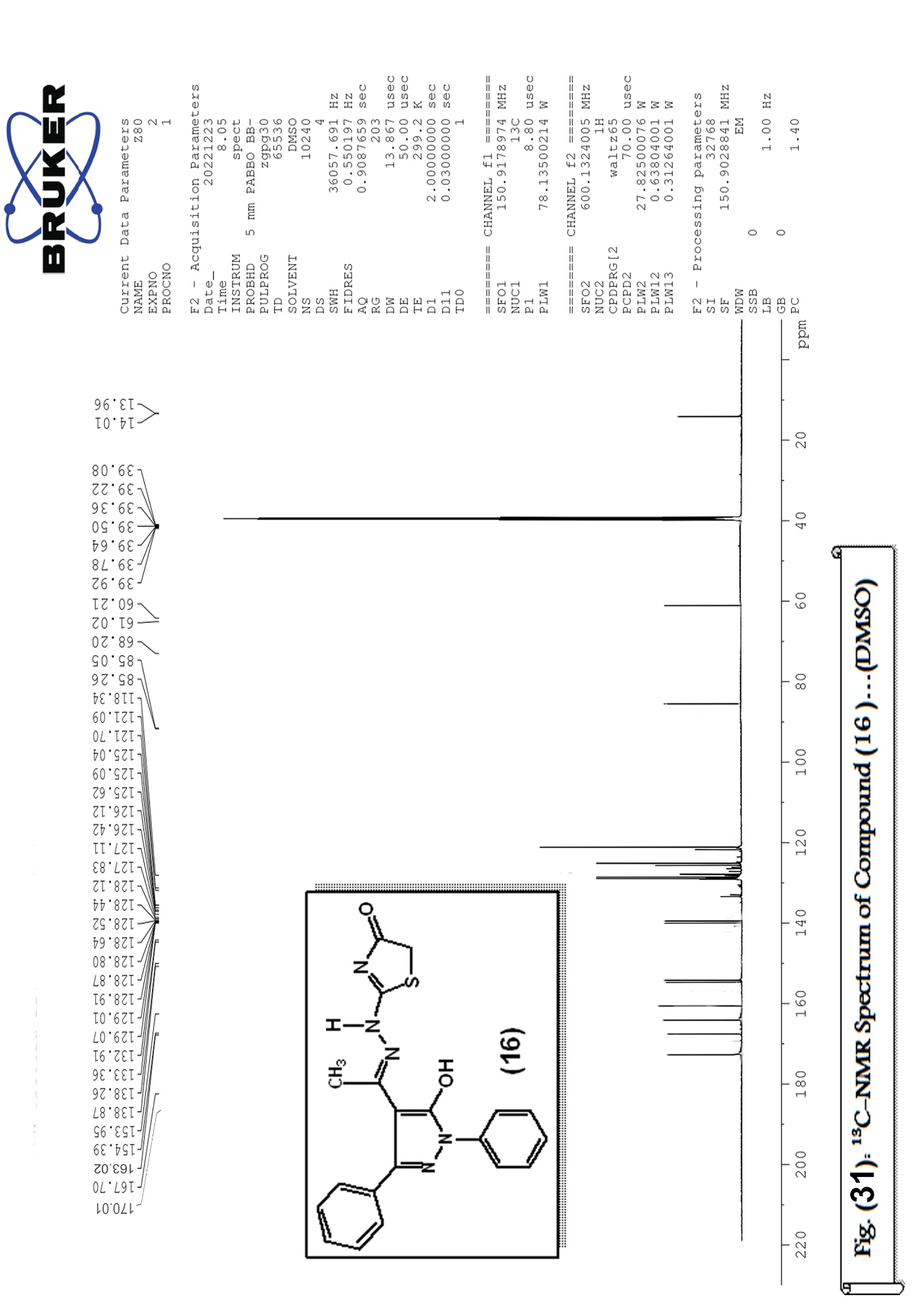


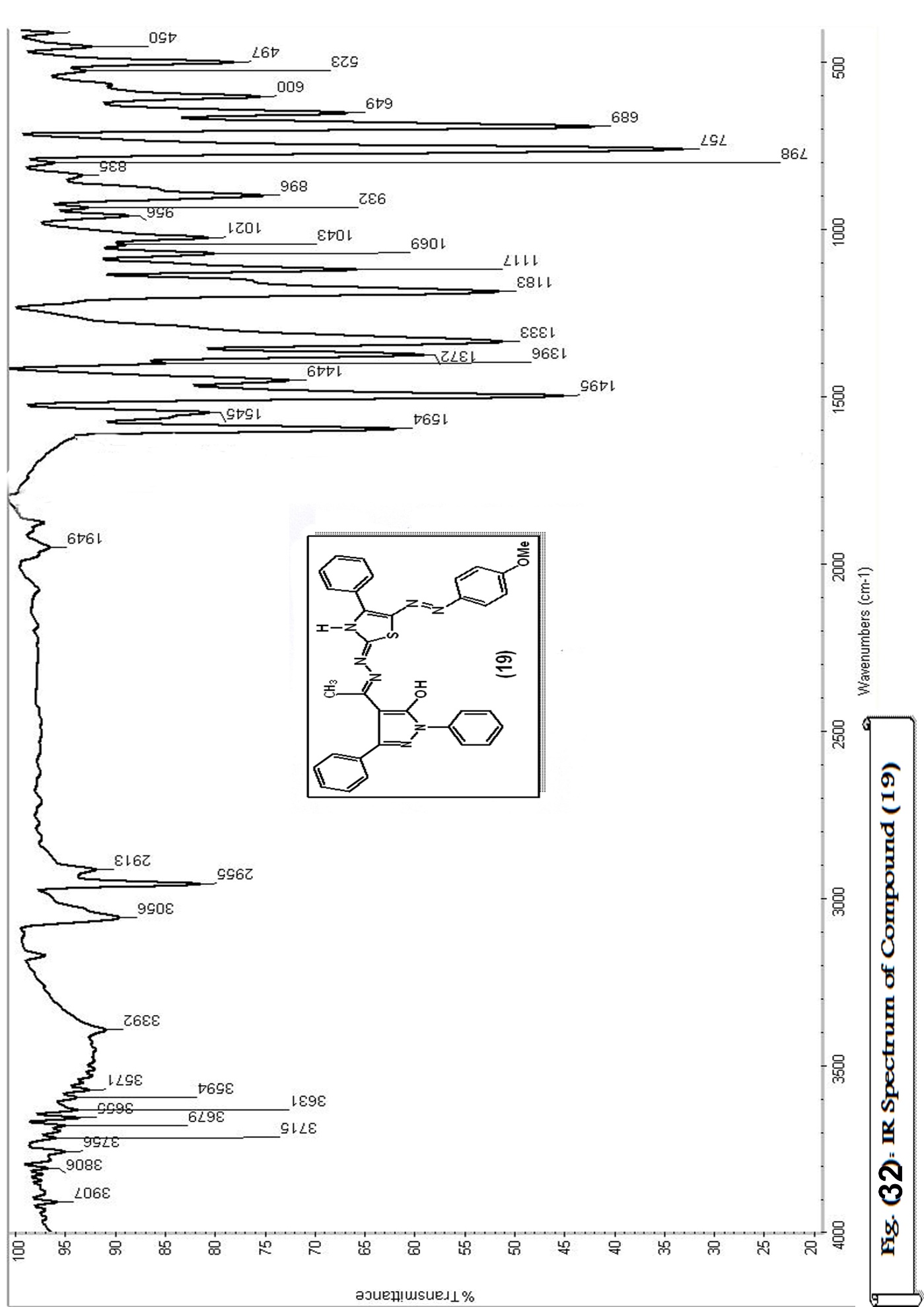


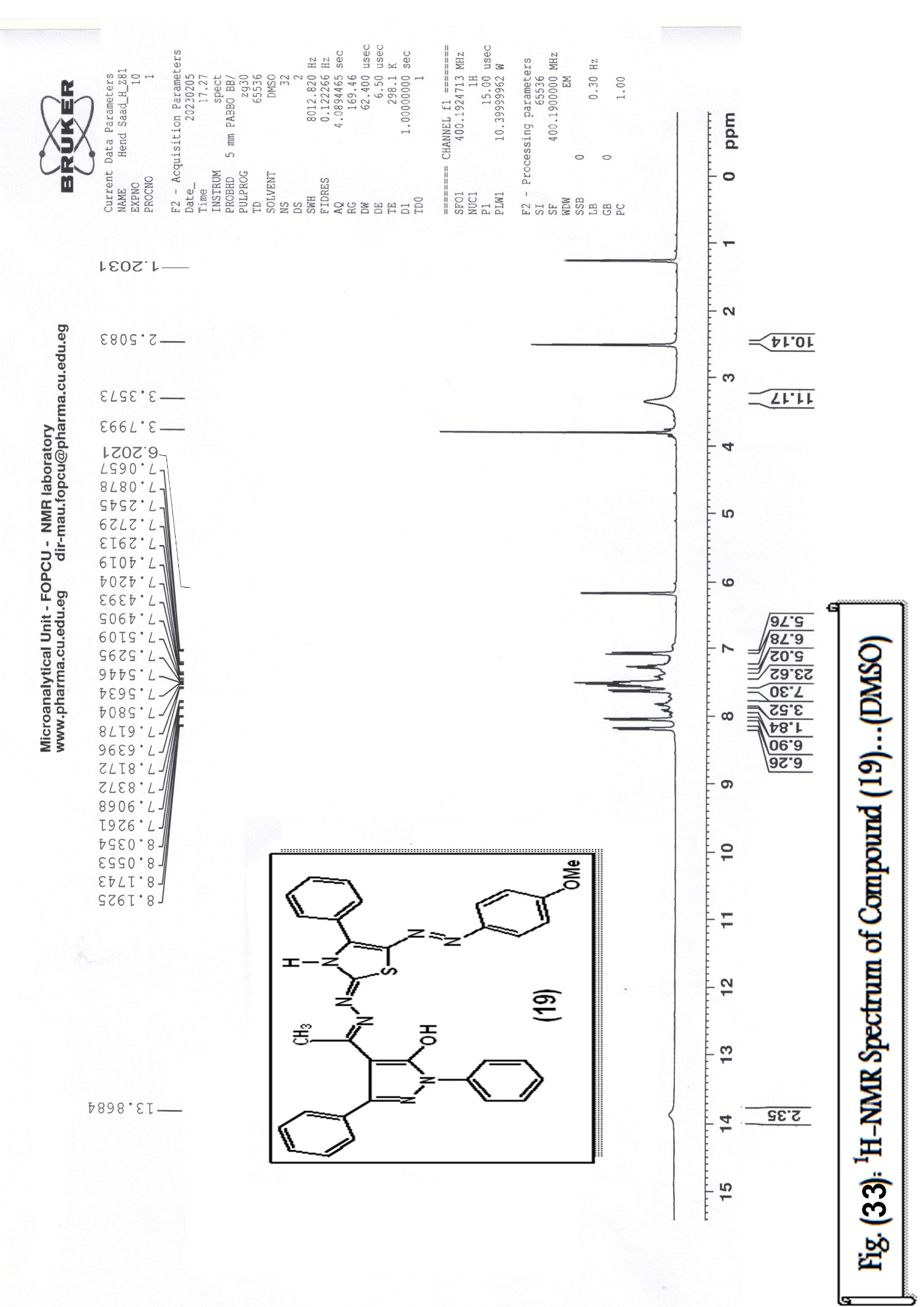


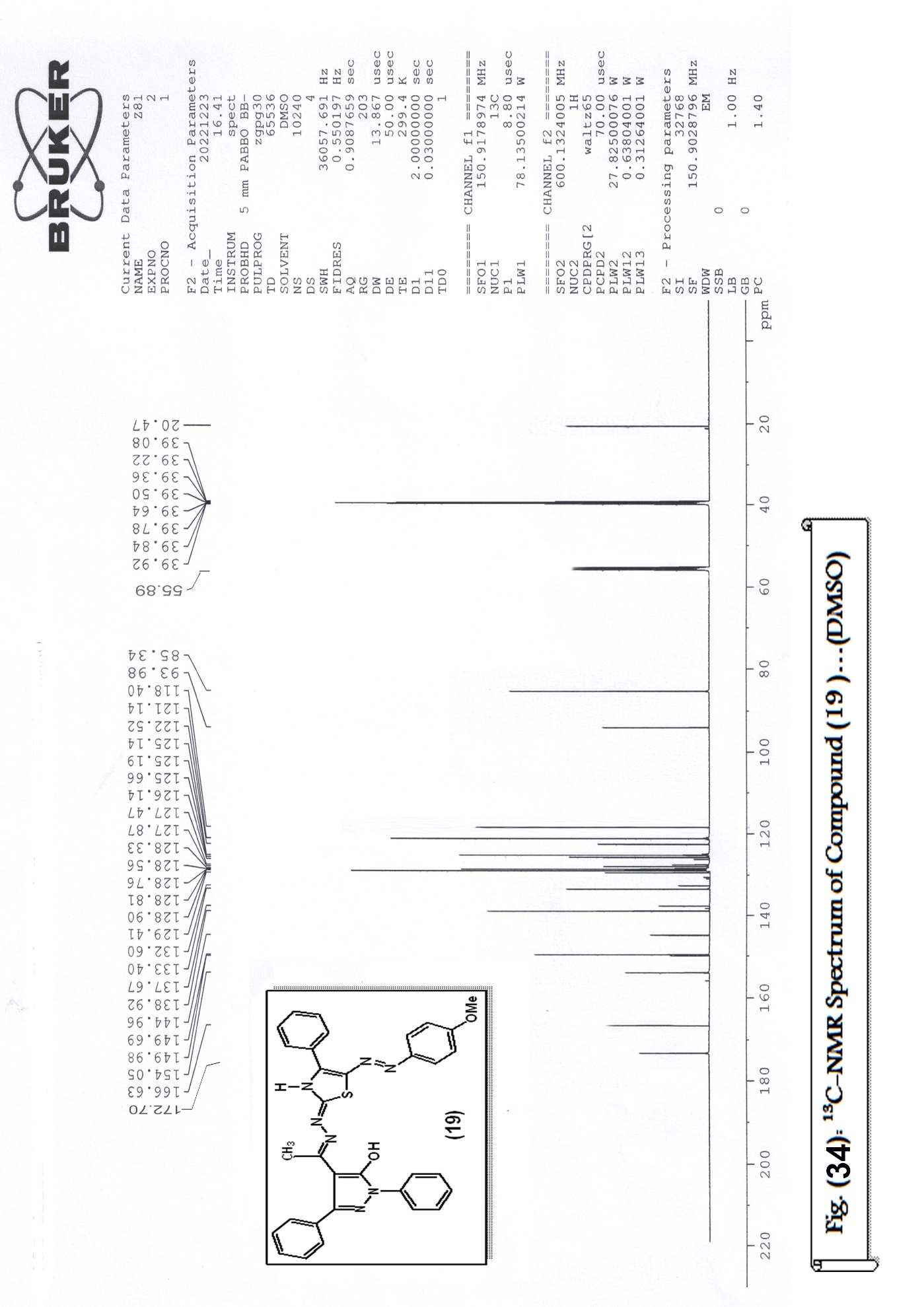


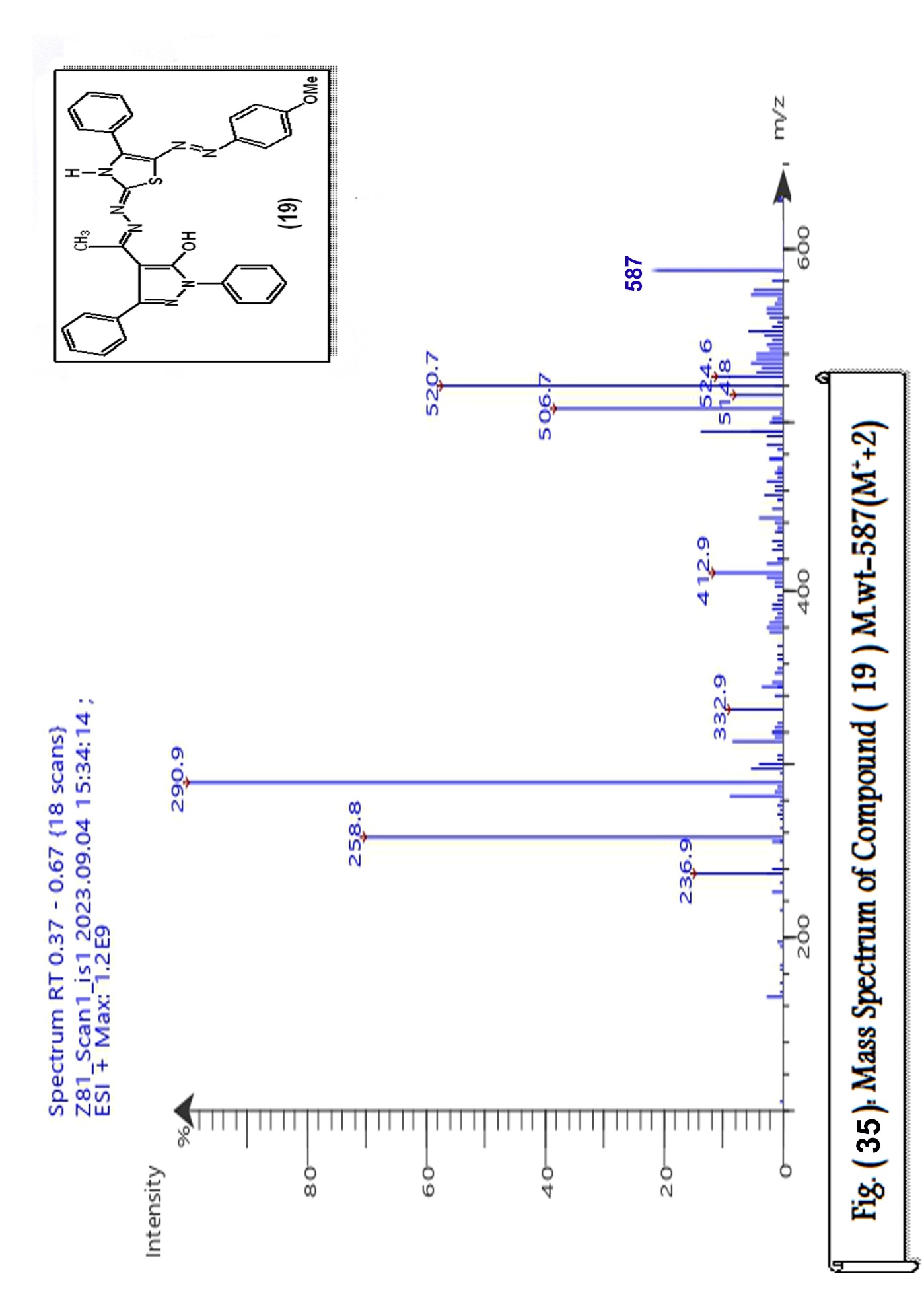


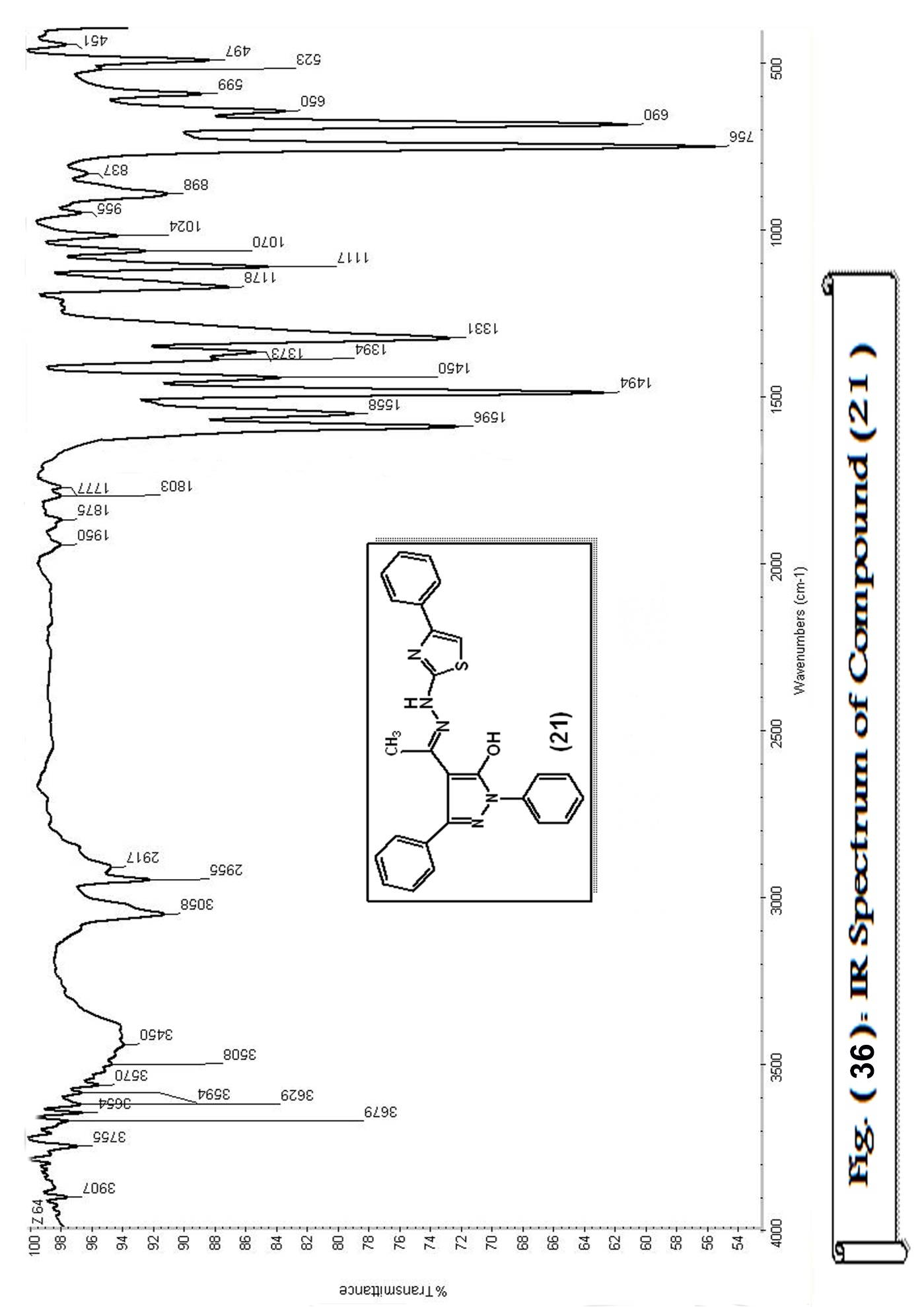


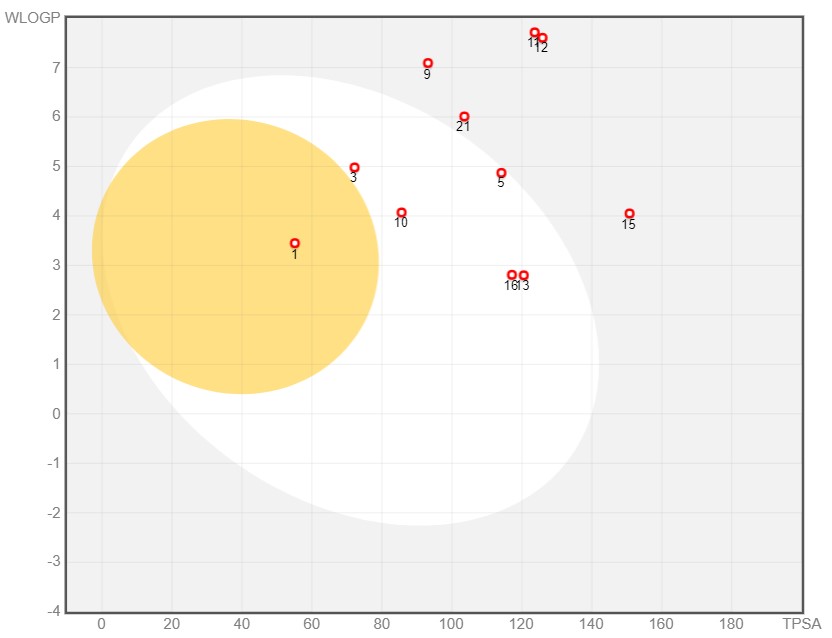


Fig. (37). BOILED-Egg model of synthesized compounds.

| 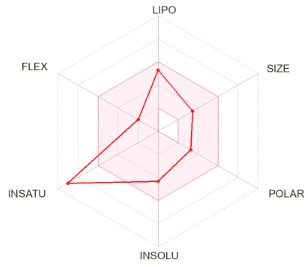  **1** | 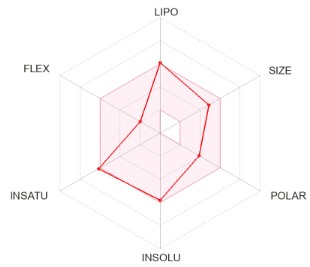  **3** | 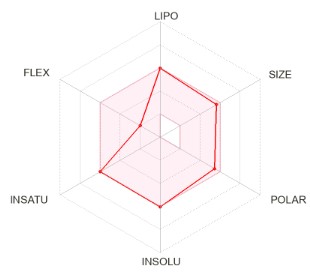  **4** |
| --- | --- | --- |
| 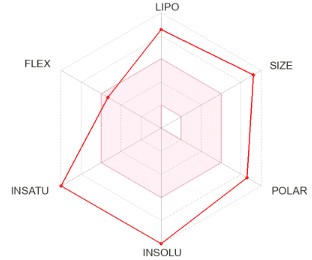  **7** | 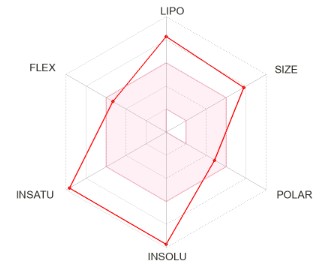  **8** | 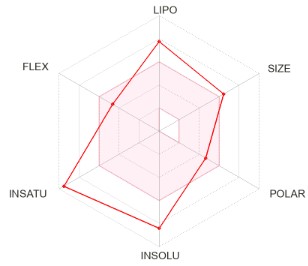  **9** |
| 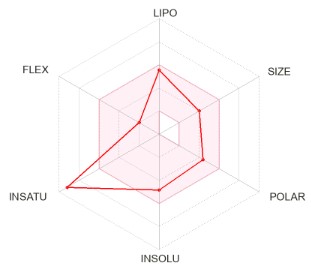  **10** | 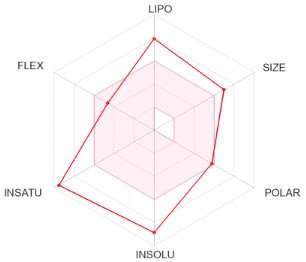  **11** | 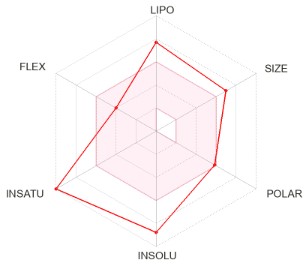  **12** |
| 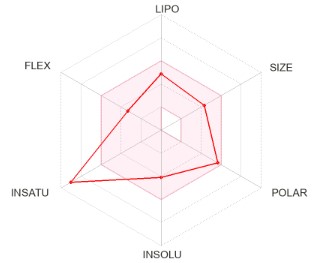  **13** | 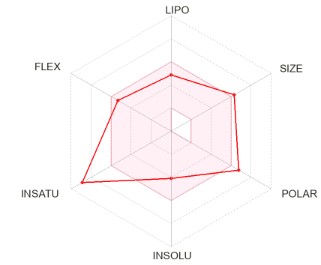  **15** | 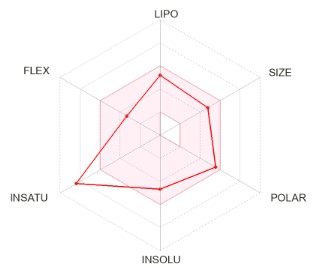  **16** |
| 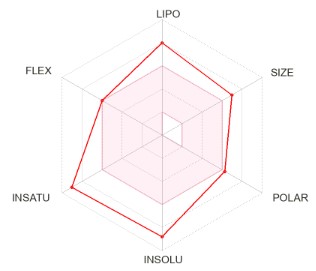  **19** | 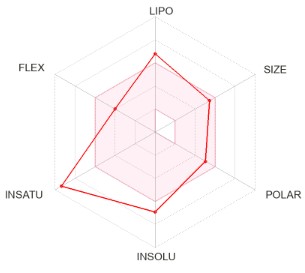  **21** |  |

**Fig. (38).** Bioavailability radar plot for target compounds

**Table 1.** Some clinically used pyrazole-containing drugs

| **Commercial Drug** | **Properties** |
| --- | --- |
| Ruxolitinib | JAK1 & JAK2 inhibitor |
| Crizotinib | ALK inhibitor |
| Ibrutinib | Bruton TK inhibitor |
| Pazopanib | VEGFR inhibitor |
| Axitinib | VEGFR inhibitor |
| Apixaban | Direct Factor Xa inhibitor |
| Sildenafil | PDE inhibitor |
| Zaleplon | GABAA Agonist |
| Allopurinol | Xanthine oxidase inhibitor |
| Celecoxib | COX-2 inhibitor |
| Metamizole | Antipyretic |

**Table 2.** Antimicrobial activity of the targeted molecules using agar-well diffusion (Inhibition zone mm)

| **Compound No.** | **Inhibition Zone (mm)** | | | | |
| --- | --- | --- | --- | --- | --- |
|  | ***Candida***  ***albicans*** | ***Bacillus***  ***subtilis*** | ***Staphylococcus aureus***  **MRSA** | ***Klebsiella***  ***pneumonia*** | ***Pseudomonas aeruginosa*** |
| **1** | 3 | 8 | 7 | 2 | ND |
| **13** | 8 | 10 | 6 | 2 | 2 |
| **7** | 4 | 4 | 3 | 3 | 3 |
| **8** | 3 | 4 | 2 | 2 | 3 |
| **21** | 5 | 6 | 6 | 3 | ND |
| **12** | 6 | 7 | 8 | 2 | 2 |
| **19** | 5 | 9 | 7 | 3 | 2 |
| **16** | 5 | 7 | 6 | 4 | ND |
| **3** | 4 | 8 | 7 | 2 | ND |
| Amphotericin B^a^ | 4 | - | | | |
| Ciprofloxacin ^b^ | - | 5 | 3 | 4 | 3 |
| ^a^ Amphotericin was used as standard antifungal agent at 20 µg/ mL  ^b^Ciprofloxacin was used as standard antibacterial agents at 20 µg/mL,  ^c^ND: not determined | | | | | |

**Table 3**. Physicochemical and pharmacokinetic properties of target compounds 1, 3, 5, and 7-10

| # | **1** | **3** | **5** | **7** | **8** | **9** | **10** |
| --- | --- | --- | --- | --- | --- | --- | --- |
| MW | 278.31 | 400.47 | 466.53 | 780.83 | 653.73 | 538.6 | 326.35 |
| #Heavy atoms | 21 | 30 | 35 | 60 | 50 | 41 | 25 |
| #Aromatic heavy atoms | 17 | 17 | 17 | 52 | 46 | 34 | 17 |
| Fraction Csp3 | 0.06 | 0.24 | 0.25 | 0 | 0.02 | 0.03 | 0.05 |
| #Rotatable bonds | 3 | 3 | 3 | 8 | 8 | 7 | 3 |
| #HBA | 3 | 4 | 5 | 8 | 6 | 5 | 4 |
| #HBD | 1 | 1 | 2 | 4 | 2 | 2 | 1 |
| MR | 81.22 | 117.6 | 131.86 | 231.18 | 196.2 | 160.22 | 95.03 |
| TPSA | 55.12 | 72.19 | 114.16 | 201.5 | 98.22 | 93.17 | 85.63 |
| iLOGP | 2.81 | 2.6 | 2.99 | 4.56 | 5.09 | 4.28 | 2.82 |
| XLOGP3 | 3.77 | 5.17 | 4.98 | 9.42 | 8.99 | 8.08 | 4.23 |
| WLOGP | 3.45 | 4.98 | 4.87 | 9.18 | 9.21 | 7.09 | 4.07 |
| MLOGP | 2.52 | 3.32 | 2.65 | 4.01 | 5.32 | 4.7 | 2.25 |
| Silicos-IT Log P | 2.92 | 4.84 | 4.06 | 6.59 | 7.04 | 5.31 | 3.06 |
| Consensus Log P | 3.09 | 4.18 | 3.91 | 6.75 | 7.13 | 5.89 | 3.28 |
| ESOL Log S | -4.34 | -5.8 | -6.03 | -10.73 | -9.71 | -8.42 | -4.83 |
| ESOL Solubility (mg/ml) | 1.27E-02 | 6.33E-04 | 4.34E-04 | 1.46E-08 | 1.28E-07 | 2.04E-06 | 4.79E-03 |
| ESOL Solubility (mol/l) | 4.55E-05 | 1.58E-06 | 9.30E-07 | 1.87E-11 | 1.95E-10 | 3.79E-09 | 1.47E-05 |
| ESOL Class | Moderately soluble | Moderately soluble | Poorly soluble | Insoluble | Poorly soluble | Poorly soluble | Moderately soluble |
| Ali Log S | -4.62 | -6.43 | -7.12 | -13.56 | -10.94 | -9.89 | -5.74 |
| Ali Solubility (mg/ml) | 6.66E-03 | 1.48E-04 | 3.57E-05 | 2.16E-11 | 7.46E-09 | 6.90E-08 | 5.95E-04 |
| Ali Solubility (mol/l) | 2.39E-05 | 3.70E-07 | 7.65E-08 | 2.77E-14 | 1.14E-11 | 1.28E-10 | 1.82E-06 |
| Ali Class | Moderately soluble | Poorly soluble | Poorly soluble | Insoluble | Insoluble | Poorly soluble | Moderately soluble |
| Silicos-IT LogSw | -5.2 | -7.42 | -7.66 | -15.55 | -14.27 | -10.06 | -5.49 |
| Silicos-IT Solubility (mg/ml) | 1.74E-03 | 1.54E-05 | 1.02E-05 | 2.20E-13 | 3.51E-12 | 4.69E-08 | 1.05E-03 |
| Silicos-IT Solubility (mol/l) | 6.27E-06 | 3.85E-08 | 2.19E-08 | 2.82E-16 | 5.37E-15 | 8.71E-11 | 3.21E-06 |
| Silicos-IT class | Moderately soluble | Poorly soluble | Poorly soluble | Insoluble | Insoluble | Insoluble | Moderately soluble |
| GI absorption | High | High | High | Low | Low | Low | High |
| BBB permeant | Yes | No | No | No | No | No | No |
| Pgp substrate | No | No | No | No | No | No | No |
| CYP1A2 inhibitor | Yes | No | No | No | No | No | Yes |
| CYP2C19 inhibitor | Yes | Yes | Yes | No | No | No | Yes |
| CYP2C9 inhibitor | Yes | Yes | Yes | No | No | No | Yes |
| CYP2D6 inhibitor | Yes | No | No | No | No | No | No |
| CYP3A4 inhibitor | No | No | Yes | No | No | No | No |
| log Kp (cm/s) | -5.32 | -5.07 | -5.61 | -4.37 | -3.9 | -3.85 | -5.29 |
| Lipinski #violations | 0 | 0 | 0 | 2 | 2 | 2 | 0 |
| Ghose #violations | 0 | 0 | 1 | 4 | 4 | 3 | 0 |
| Veber #violations | 0 | 0 | 0 | 1 | 0 | 0 | 0 |
| Egan #violations | 0 | 0 | 0 | 2 | 1 | 1 | 0 |
| Muegge #violations | 0 | 1 | 0 | 4 | 3 | 1 | 0 |
| Bioavailability Score | 0.55 | 0.55 | 0.56 | 0.17 | 0.17 | 0.17 | 0.55 |

**Table 4.** Physicochemical and pharmacokinetic properties of target compounds 11-13, 15-16, 19, and 21

| **#** | **11** | **12** | **13** | **15** | **16** | **19** | **21** |
| --- | --- | --- | --- | --- | --- | --- | --- |
| MW | 586.64 | 586.64 | 351.43 | 525.58 | 391.45 | 585.68 | 451.54 |
| #Heavy atoms | 45 | 45 | 25 | 38 | 28 | 43 | 33 |
| #Aromatic heavy atoms | 34 | 40 | 17 | 23 | 17 | 34 | 28 |
| Fraction Csp3 | 0.03 | 0 | 0.06 | 0.07 | 0.1 | 0.06 | 0.04 |
| #Rotatable bonds | 7 | 6 | 5 | 8 | 5 | 8 | 6 |
| #HBA | 6 | 5 | 3 | 7 | 5 | 7 | 4 |
| #HBD | 2 | 3 | 3 | 3 | 2 | 2 | 2 |
| MR | 174.03 | 175.6 | 102.59 | 153.4 | 114.39 | 170.2 | 134.42 |
| TPSA | 123.68 | 125.91 | 120.55 | 150.79 | 117.17 | 140.75 | 103.57 |
| iLOGP | 4.1 | 4.46 | 3.25 | 3.25 | 2.43 | 2.43 | 3.26 |
| XLOGP3 | 8.37 | 7.98 | 3.03 | 3.03 | 3.61 | 3.61 | 6.4 |
| WLOGP | 7.71 | 7.6 | 2.8 | 4.05 | 2.81 | 8.05 | 6.01 |
| MLOGP | 4.32 | 4.59 | 2.26 | 2.26 | 2.66 | 2.66 | 3.96 |
| Silicos-IT Log P | 5.54 | 5.35 | 2.95 | 2.95 | 3.46 | 3.46 | 5.47 |
| Consensus Log P | 6.01 | 6 | 2.86 | 2.86 | 2.99 | 2.99 | 5.02 |
| ESOL Log S | -8.85 | -8.77 | -4.1 | -4.1 | -4.66 | -4.66 | -6.9 |
| ESOL Solubility (mg/ml) | 8.34E-07 | 1.00E-06 | 2.79E-02 | 2.79E-02 | 8.55E-03 | 8.55E-03 | 5.64E-05 |
| ESOL Solubility (mol/l) | 1.42E-09 | 1.71E-09 | 7.93E-05 | 7.93E-05 | 2.18E-05 | 2.18E-05 | 1.25E-07 |
| ESOL Class | Poorly soluble | Poorly soluble | Moderately soluble | Moderately soluble | Moderately soluble | Moderately soluble | Poorly soluble |
| Ali Log S | -10.83 | -10.48 | -5.23 | -5.23 | -5.76 | -5.76 | -8.37 |
| Ali Solubility (mg/ml) | 8.60E-09 | 1.96E-08 | 2.08E-03 | 2.08E-03 | 6.84E-04 | 6.84E-04 | 1.94E-06 |
| Ali Solubility (mol/l) | 1.47E-11 | 3.34E-11 | 5.93E-06 | 5.93E-06 | 1.75E-06 | 1.75E-06 | 4.29E-09 |
| Ali Class | Insoluble | Insoluble | Moderately soluble | Moderately soluble | Moderately soluble | Moderately soluble | Poorly soluble |
| Silicos-IT LogSw | -10.31 | -11.82 | -5.15 | -5.15 | -6.19 | -6.19 | -9.21 |
| Silicos-IT Solubility (mg/ml) | 2.86E-08 | 8.94E-10 | 2.47E-03 | 2.47E-03 | 2.51E-04 | 2.51E-04 | 2.82E-07 |
| Silicos-IT Solubility (mol/l) | 4.88E-11 | 1.52E-12 | 7.01E-06 | 7.01E-06 | 6.41E-07 | 6.41E-07 | 6.24E-10 |
| Silicos-IT class | Insoluble | Insoluble | Moderately soluble | Moderately soluble | Poorly soluble | Poorly soluble | Poorly soluble |
| GI absorption | Low | Low | High | High | High | High | Low |
| BBB permeant | No | No | No | No | No | No | No |
| Pgp substrate | No | No | No | No | No | No | No |
| CYP1A2 inhibitor | No | No | No | No | No | No | No |
| CYP2C19 inhibitor | No | No | No | No | Yes | Yes | Yes |
| CYP2C9 inhibitor | No | No | Yes | Yes | Yes | Yes | Yes |
| CYP2D6 inhibitor | No | No | No | No | No | No | No |
| CYP3A4 inhibitor | No | No | No | No | No | No | No |
| log Kp (cm/s) | -3.94 | -4.21 | -6.29 | -6.29 | -6.12 | -6.12 | -4.51 |
| Lipinski #violations | 2 | 2 | 0 | 0 | 0 | 0 | 0 |
| Ghose #violations | 4 | 4 | 0 | 0 | 0 | 0 | 2 |
| Veber #violations | 0 | 0 | 0 | 0 | 0 | 0 | 0 |
| Egan #violations | 1 | 1 | 0 | 0 | 0 | 0 | 1 |
| Muegge #violations | 1 | 1 | 0 | 0 | 0 | 0 | 1 |
| Bioavailability Score | 0.17 | 0.17 | 0.55 | 0.55 | 0.55 | 0.55 | 0.55 |
